# Transcriptome and Metabolome Analysis Unveil Anthocyanin Metabolism in Pink and Red Testa of Peanut (*Arachis hypogaea* L.)

**DOI:** 10.1155/2021/5883901

**Published:** 2021-08-06

**Authors:** Qiqin Xue, Xiurong Zhang, Hui Yang, Huadong Li, Yuying Lv, Kun Zhang, Yongguang Liu, Fengzhen Liu, Yongshan Wan

**Affiliations:** ^1^State Key Laboratory of Crop Biology, Shandong Key Laboratory of Crop Biology, College of Agronomy, Shandong Agricultural University, Tai'an, 271018 Shandong, China; ^2^Weifang University of Science and Technology, Shouguang, 262700 Shandong, China

## Abstract

Peanut (*Arachis hypogaea* L.) is an important source of oil and food around the world, and the testa color affects its appearance and commercial value. However, few studies focused on the mechanism of pigment formation in peanut testa. In this study, cultivars Shanhua 15 with pink testa and Zhonghua 12 with red testa were used as materials to perform the combined analysis of transcriptome and metabolome. A total of 198 flavonoid metabolites were detected, among which petunidin 3-O-glucoside and cyanidin O-acetylhexoside in Zhonghua12 were 15.23 and 14.72 times higher than those of Shanhua 15 at the R7 stage, revealing the anthocyanins underlying the red testa. Transcriptome analysis showed that there were 6059 and 3153 differentially expressed genes between Shanhua 15 and Zhonghua 12 in different growth periods, respectively. These differentially expressed genes were significantly enriched in the flavonoid biosynthesis, biosynthesis of secondary metabolites, and metabolic pathways. Integrated analysis of transcriptome and metabolome indicated CHS gene (*arahy.CM90T6*), F3′H genes (*arahy. 8F7PE4* and *arahy. K8H9R8*), and DFR genes (*arahy. LDV9QN* and *arahy. X8EVF3*) may be the key functional genes controlling the formation of pink and red testa in peanut. Transcription factors MYB (*arahy.A2IWKV*, *arahy.US2SKM*, *arahy.SJGE27*, *arahy.H8DJRL*, and *arahy.PR7AYB*), bHLH (*arahy.26781*N, *arahy.HM1IVV*, and *arahy.MP3D3D*), and WD40 (*arahy.L6JJW9*) in the biosynthetic pathway of anthocyanin were significantly upregulated in Zhonghua 12 which may be the key regulatory genes in testa pigment formation. This is a comprehensive analysis on flavonoid metabolites and related genes expression in peanut testa, providing reference for revealing the regulatory mechanism of pigment accumulation in peanut testa.

## 1. Introduction

Anthocyanin is a natural hydrosoluble pigment widely found in plant leaves, stems, flowers, fruits, and seeds. It is mainly synthesized on the surface of the endoplasmic reticulum and accumulated in vacuoles. Anthocyanins have a wide range of biological functions, making plant organs blue, pink, red, or purple [1]. In many plants, anthocyanins can protect them from drought stress, cold, ultraviolet radiation, and microorganisms. Besides, they make flowers and fruits colorful and attract animals and insects, thus promoting pollination and seed transmission [2–5]. In addition to the natural character, anthocyanins also have high nutritional value and antioxidant capacity. They can scavenge pathogenic free radicals in the human body [6], inhibit the oxidation of low-density lipids, and prevent cardiovascular and cerebrovascular diseases, cancer, and other chronic diseases [1, 7].

The biosynthesis of anthocyanins and other flavonoids consists of many enzymatic reactions [8], which can be divided into three steps. First, 4-coumaryl-CoA is synthesized from phenylalanine, which is catalyzed by phenylalanine ammonia-lyase (PAL), cinnamate 4-hydroxylase (C4H), and 4-coumaryl-CoA ligase (4CL). These reactions are very common in the secondary metabolism of most plants. Then, 4-coumaryl-CoA and malonyl-CoA are successively catalyzed by chalcone synthase (CHS), chalcone isomerase (CHI), flavanone 3-hydroxylase (F3H), flavanone 3′-hydroxylase (F3′H), and flavonoid 3′5′-hydroxylase (F3′5 ′H) to synthesize dihydrokaempferol, which is a key step in the synthesis of flavonoids. Finally, dihydrokaempferol are synthesized by dihydroflavonol 4-reductase (DFR), anthocyanidin synthase (ANS), and UDP-glucoside: flavonoid glucosyltransferase (UFGT) [9–12]. In addition, anthocyanin biosynthesis is regulated by a variety of transcription factors (TFs), including basic helix-loop-helix (bHLH), R2R3-MYB, WD40-repeat protein, NAC, WRKY, bZIP, and MADS-box [13–15]. Among them, transcription factors R2R3-MYB, bHLH, and WD40 can form MYB-bHLH-WD40 (MBW) complex and regulate the expression level of anthocyanin biosynthesis genes in most plants [16–18].

Peanut (*Arachis hypogaea* L.) is an important oil and economic crop in the world, and the testa color affects its commercial and nutritional value. There are many colors of peanut testa, including pink, red, purple, black, white, and multicolor, the most common of which are pink and red. However, there are only a few reports on the genetic mechanism of testa color in peanut. For example, the AhTc1 gene, encoding a R2R3-MYB transcription factor, which is located in the 4.7Mb region on chromosome A10, is responsible for the purple testa [19], while a single dominant gene located in the 0.905cM region on chromosome 3 controls the red seed coat, and the upregulation of anthocyanin synthesis genes may lead to red testa [20]. The genes PAL, C4H, CHS, CHI, and homologs of AtMYB111 in the flavonoid biosynthesis pathway may be the main regulators for pink pigment accumulation in peanut seed testa [21]. The difference of testa color is closely related to the expression of anthocyanin synthesis-related genes and the accumulation of metabolites. However, there is few research on the mechanism of different color formation in peanut seed by combining transcriptome and metabolome methods. At present, the gene regulation network of testa color formation in peanut is still unclear.

In the present study, seed pigment accumulation differences were investigated between two varieties, Shanhua 15 (pink testa) and Zhonghua 12 (red testa), during the growth period of R4 and R7, based on a previous classification of peanut pod developmental stages [22]. Meanwhile, the expression of flavonoid-related metabolites and corresponding genes was also analyzed by combined transcriptome and metabolome methods. The results will not only provide a new strategy for the study of gene function and the relationship between genes and metabolites in peanut but also provide reference information for revealing the formation and regulation mechanism of different testa colors in peanut.

## 2. Materials and Methods

### 2.1. Plant Materials

Cultivars Shanhua 15 with pink testa and Zhonghua 12 with red testa were used as materials, and they were planted in the test field of the agricultural experimental station of Shandong Agricultural University (36.15°N, 117.15°E), Tai'an, China. According to a previous study, peanut pod development can be divided into nine stages, R1 to R9 [22]. In this study, seeds with identical size were selected from Shanhua 15 and Zhonghua 12 from R2 and R7 stages, respectively, and the testa was peeled off the seeds, frozen immediately in liquid nitrogen, and stored in a low-temperature refrigerator at -80°C for follow-up analysis. The peanut seeds begin to pigment deposition in testa within the R4 stage, and they are nearly mature with the almost completed coloration in the R7 stage; thus, testa samples of Shanhua 15 and Zhonghua 12 at R4 and R7 stage, named S4 and S7 and Z4 and Z7, respectively, were used for transcriptome and metabolome analysis.

### 2.2. Total Anthocyanin Content Determination

For extraction of anthocyanin, the method described by Rabino and Mancinelli [23] with some optimization was as followed. Testa samples were powdered in a mortar by adding liquid nitrogen; then, 0.1 g powder was mixed with 5 ml ethanol mixture (85 : 15 95% ethanol : 1.5 M HCl, *v*/*v*) and stored overnight at 4°C. The mixture was centrifuged at 5000 × g for 6 mins, and the supernatant containing anthocyanin was used to absorbance measurement at 530, 620, and 650 nm. Total anthocyanin content was calculated by the following formula: anthocyanin content (mmol^.^g^−1^ FW) = [(*A*1–*A*2) − 0.1 × (*A*3–*A*2)]/4.62 × 10^4^ × *V*/*m* × 106. *A*1, *A*2, and *A*3 represent the absorbance at 530, 620, and 650 nm, respectively, and *V* represents the extract volume, and *m* represents sample weight. The value 4.62 × 10^4^ represents the extinction coefficient of anthocyanin at 530 nm. Three replicates were analyzed for each sample.

### 2.3. Flavonoids-Metabolites Detection and Multiple Reaction Monitoring (MRM)

Flavonoids-metabolites identification and quantification was carried out by Metware Biotechnology Co., Ltd. (Wuhan, China) using LC-ESI-MS/MS system (HPLC, Shim-pack UFLC Shimadzu CBM30A system, http://www.shimadzu.com.cn/; MS, Applied Biosystems 6500Q TRAP, http://www.appliedbiosystems.com.cn/). Detailed operation procedures were performed according to the methods described by Dong et al. [1]. Flavonoids-metabolites identification was adopted by Partial least square discriminant analysis (PLS-DA). The variable importance in projection (VIP) of the first principal component represents the contribution of the differential metabolites between two groups, and the differential metabolites were screened using thresholds of fold change (FC) ≥2 or ≤0.5 and VIP >1.

### 2.4. Transcriptome Analysis

Total RNA extraction, concentration, and cDNA library construction were performed according to methods described by Cao et al. [24]. With three biological replicates for each sample, a total of twelve libraries were constructed for samples S4, S7, Z4, and Z7. Then, the library products were sequenced on the HiSeq 4000 platform (Illumina, San Diego, CA, USA). After removing reads containing the adapter and low-quality sequences, the resulting high-quality clean data was mapped to the peanut reference genome (https://www.peanutbase.org/data/public/Arachis_hypogaea/).

The fragments per kb per million reads (FPKM) method was used to calculate gene expression [25]. Differential expression analysis of testa samples was performed by the DEGseq R package, and the *P* values were adjusted using *q* values [26]. Differentially expressed genes (DEGs) were screened by the thresholds of log2 (FC) ≥1 and False Discovery Rate (FDR) ≤0.05. Multiple databases were used for gene function annotation, including Kyoto Encyclopedia of Genes and Genomes (KEGG), Gene Ontology (GO), Clusters of Orthologous Groups (COG) of proteins database, NCBI non-redundant protein sequences (Nr), a manually annotated and reviewed protein sequence database Swiss-Prot, and database of TrEMBL that contains all translations of EMBL nucleotide sequence entries.

### 2.5. Validation of Transcriptome Profile

To validate gene expression results, nine flavonoid-related genes were randomly selected for quantitative real-time PCR (qRT-PCR) reactions, and the specific primers (Table S1) were designed by Primer Premier 5 software. The reaction was carried out according to the instructions of the SuperReal PreMix Plus kit (TIANGEN, Beijing, China), and the reaction system included 10 *μ*L SYBR PreMix Plus, 1 *μ*L each primer (10 *μ*M), 2 *μ*L cDNA template, and 6 *μ*L RNase-free water. It was performed on the ABI 7500 PCR instrument (Applied Biosystems), with the following protocol: 95°C for 30 s, followed by 40 cycles at 95°C for 10 s, 55°C for 20 s, 72.0°C for 20 s, and 75.0°C for 5 s. Peanut *Actin* gene was selected as the housekeeping gene [27], and relative gene expression was analyzed by the 2^-*△△*Ct^ method. Significant differences between samples were performed by standard deviation.

## 3. Results

### 3.1. Testa Anthocyanin Content Changes during Seed Development

**Figure 1:**
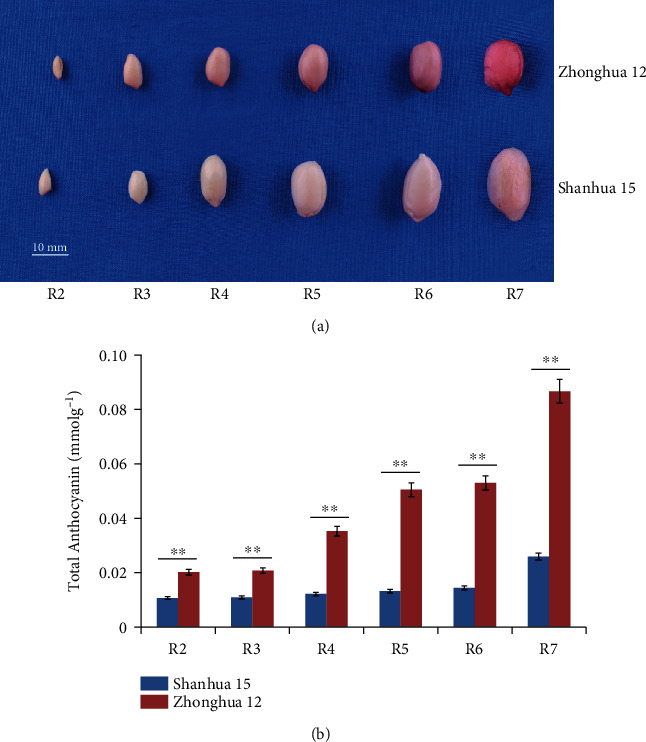
Phenotypic characterization of peanut testa at different developmental stages. (a) The phenotype of *Arachis hypogaea* cv.Zhonghua12 and Shanhua15 seed kernel from R2 to R7 stages. (b) Changes in anthocyanins during Shanhua15 and Zhonghua12 testa development.

As shown in Figure 1(a), the testa color of Shanhua 15 and Zhonghua 12 got deeper gradually from R2 to R7, especially in R7, when the seeds were colored completely and entered the mature stage. Testa of Shanhua 15 is pink, while it is red in Zhonghua 12. Anthocyanin content determination also showed that the total testa anthocyanin content increased gradually with the seed development and reached the highest level at the R7 stage, which were 0.026 mmol.g^−1^ and 0.087 mmol.g^−1^ in Shanhua 15 and Zhonghua 12, respectively (Figure 1(b)). The total testa anthocyanin content of Zhonghua 12 was significantly higher than that of Shanhua 15 at each stage (*p* < 0.01), with approximately three times higher on average (Figure 1(b)). Therefore, it is suggested that anthocyanin accumulation difference is cultivar specific and eating peanuts with red testa is more beneficial to health.

### 3.2. Testa Metabolites Differences between Shanhua15 and Zhonghua12

**Figure 2:**
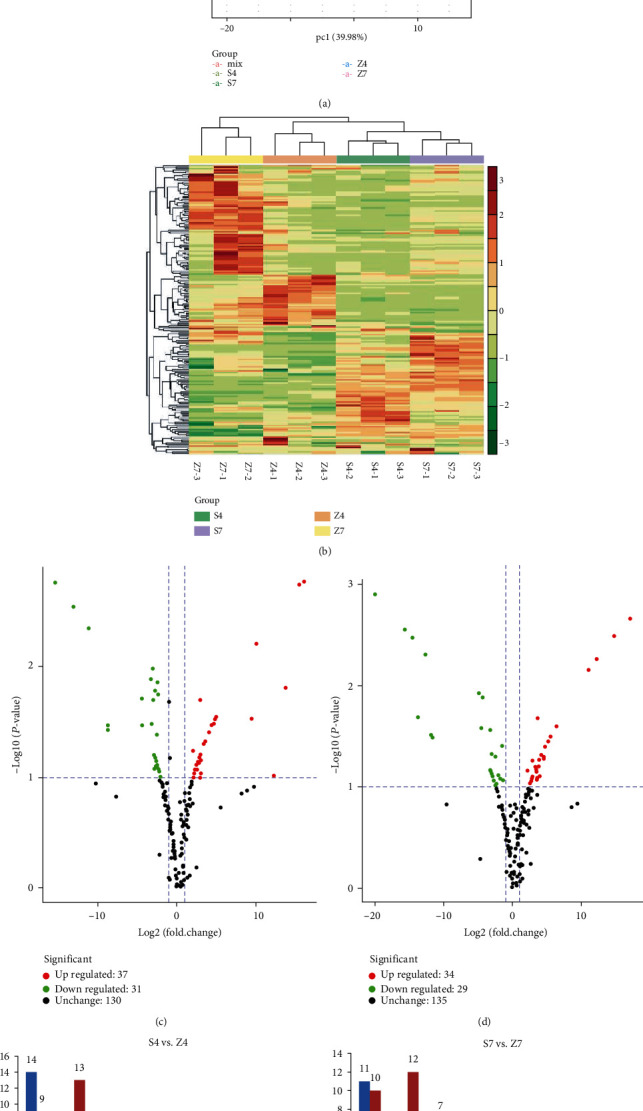
Significantly changed metabolites (SCMs) in Shanhua15 testa compared to those in Zhonghua12. (a) PCA score plot of metabolites profiles from the Shanhua15 and Zhonghua12 testa. Every point represents an independent biological replicate. (b) Expression patterns of secondary metabolites revealed by metabolomic analysis. (c, d) Volcano plot of the metabolites between Shanhua15 and Zhonghua12. (e, f) Number of SCMs in different categories. S4, Shanhua15 R4 pod period; Z4, Zhonghua12 R4 pod period; S7, Shanhua15 R7 pod period; Z7, Zhonghua12 R7 pod period. -1, -2, and -3 represent the three biological replicates.

In order to further study the composition of peanut testa, samples of Shanhua 15 (S4 and S7) and Zhonghua 12 (Z4 and Z7) testa at the R4 and R7 stage were used for flavonoid-related metabolite profiles analysis. A total of 198 flavonoid-related metabolites were detected in all samples (Table S2). Principal component analysis (PCA) showed that S4, S7, Z4, and Z7 were obviously separated in the PC1 × PC2 score chart (Figure 2(a)), and obvious differences were showed between both cultivars and growth periods (Figure 2(b)). The volcano plot also indicated that there were significant differences in metabolites content between the two varieties at R4 (Figure 2(c)) and R7 (Figure 2(d)) stages. The significantly changed metabolites (SCMs) could be divided into eight categories, including anthocyanins, proanthocyanidins, flavanone, flavone, flavonoid, flavonol, isoflavone, and polyphenol. Compared with S4, 68 SCMs were detected in Z4, 37 of which were upregulated and 31 were downregulated (Figure 2(e)), respectively, while 63 SCMs were identified in Z7 when comparing to those in S7, among which 34 SCMs were upregulated and 29 were downregulated, respectively (Figure 2(f)). The category with the most SCMs was flavone, followed by flavonol.

Anthocyanin plays an important role in the coloration of seed coat and fruit skin [12, 21]. Eleven anthocyanins were identified from all samples. The content of cyanidin O-diacetyl-hexoside-O-glyceric acid in Z4 was 7.87 times higher than that in S4, while pelargonin content decreased significantly (Table S2). In Z7 testa, the contents of petunidin 3-O-glucoside and cyanidin O-acetylhexoside increased by 15.23 times and 14.72 times compared to S7, respectively (Table S2). The results suggested that the content of cyanidin O-diacetyl-hexoside-O-glyceric acid possibly leads to the color difference of peanut testa in the R4 stage, but petunidin 3-O-glucoside and cyanidin O-acetylhexoside possibly play an important role in the testa color formation in the R7 stage.

### 3.3. Transcriptome Analysis of Shanhua15 and Zhonghua12

In order to further analyze the regulation mechanism of flavonoids and anthocyanin biosynthesis in the process of testa color formation, transcriptome sequencing was performed at the R4 and R7 stages using testa samples from Shanhua 15 and Zhonghua 12. With three biological replicates for each sample, twelve cDNA libraries were constructed and sequenced on the HiSeq4000 platform. The original sequencing data were stored in the NCBI Short Read Archive database (SRA, http://www.ncbi.nlm.nih.gov/Traces/sra_sub/sub.cgi) with the BioSample accession number SRP271546 (BioProject ID: PRJNA638812). After removing the adaptors and low-quality reads, 57140101, 59405717, 56610333, and 48707327 clean reads were obtained from S4, S7, Z4, and Z7 libraries, respectively. Q20 value, proportion of nucleotides with quality value >20, was above 97% (Table S3), indicating that the RNA-Seq results were of high quality and suitable for follow-up analysis. Finally, 51299 genes were subsequently assembled from the four libraries.

**Figure 3:**
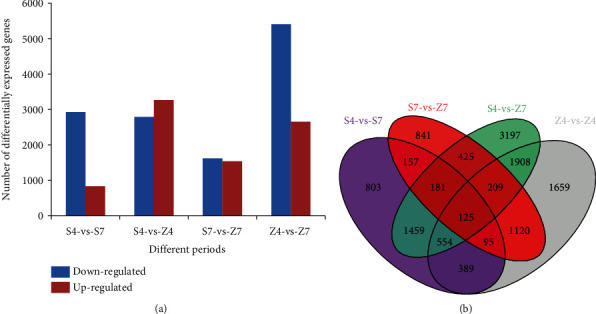
Functional annotation and classification of differentially expressed genes between R4 and R7 pod stages of Shanhua15 and Zhonghua12. (a) Number of differentially expressed genes. (b) Venn diagram. S4, Shanhua15 R4 pod period; Z4, Zhonghua12 R4 pod period; S7, Shanhua15 R7 pod period; Z7, Zhonghua12 R7 pod period.

Taking log_2_ (FC) ≥1 and FDR ≤0.05 as the thresholds, differentially expressed genes (DEGs) were screened out. A total of 3,266 and 1,536 upregulated genes and 2,793 and 1,617 downregulated genes were identified in S4-vs-Z4 and S7-vs-Z7, respectively. For one cultivar, 836 upregulated and 2,927 downregulated genes were detected in S4-vs-S7, while 2,652 upregulated and 5,406 downregulated genes were detected in Z4-vs-Z7, respectively (Figure 3(a)). Venn diagram of all DEGs detected in four compared groups, S4-vs-Z4, S7-vs-Z7, S4-vs-S7, and Z4-vs-Z7, displayed that 125 common DEGs could be detected in each group (Figure 3(b)).

In the S7-vs-Z7 group, ontology analysis showed that 7198, 3104, and 7284 genes were assigned to cell component, molecular functional, and biological process class, respectively (Figure S1). Based on enrichment results, 58 DEGs were enriched in the secondary metabolite biosynthetic process (GO:0044550), including 32 upregulated and 26 downregulated genes, and it was the largest group (Table S4). From COG annotation, 1781 DEGs in the S7-vs-Z7 group were classified into 25 categories, and the largest class was the general functional cluster prediction only (318 genes, 17.85%), followed by signaling mechanism (219 genes, 12.30%), secondary metabolites biosynthesis, transport, and catabolism (156 genes, 8.76%) (Figure S2).

The enriched metabolic pathways analysis showed 1349 DEGs from the S4-vs-Z4 group were enriched into 280 KEGG pathways, while 679 DEGs from S7-vs-Z7 were enriched into 268 pathways. Five categories, cellular processes, environmental information processing, genetic information processing, metabolism, and organismal systems, were revealed from KEGG classification analysis. Pathways of biosynthesis of secondary metabolites, flavonoid, and cell cycle-yeast were significantly changed at the R4 stage, while circadian rhythm-plant, biosynthesis of flavonoid, secondary metabolites, and phenylpropanoid were the significantly changed pathways at the R7 stage (Table 1).

### 3.4. Differential Expression Genes Related to Flavonoid and Anthocyanin Biosynthesis

As shown in Table S2, a large number of flavonoids were detected in the testa of Shanhua15 and Zhonghua12. We finally rearranged 8 major compounds of flavonoids (Figures 2(e) and 2(f)). A total of 34 DEGs detected in S4-vs-Z4 were associated with flavonoid and anthocyanin biosynthesis, and 16 related DEGs were detected in S7-vs-Z7 (Table S5).

**Figure 4:**
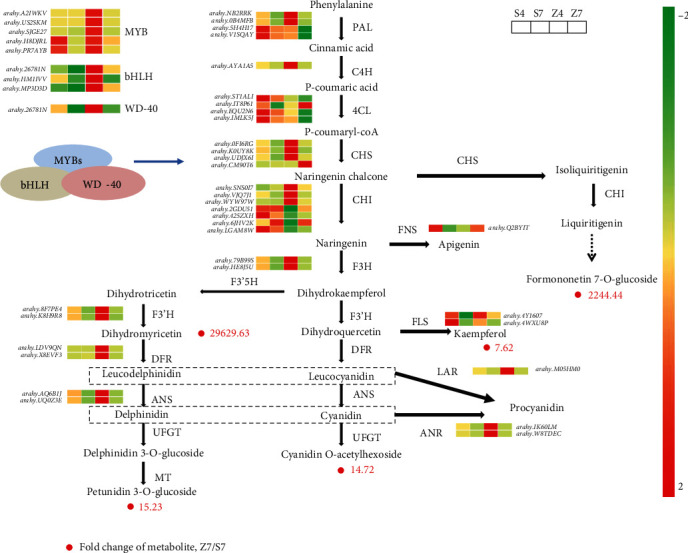
Transcript profiling of genes in the flavonoid and anthocyanin biosynthetic pathways in Shanghua15 and Zhonghua12 at R7 stages. Heat map represents changes in transcripts in flavonoid and anthocyanin biosynthetic. Dots marked with red background represent increased abundances of metabolites. PAL: phenylalanine ammonia-lyase; C4H: cinnamic acid 4-hydroxylase; 4CL: 4-coumarate CoA ligase; CHS: chalcone synthase; CHI: chalcone isomerase; F3′H: flavanoid 3′-hydroxylase; DFR: dihydroflavonol 4-reductase; ANS: anthocyanidin synthase; FLS: flavonol synthesis; FNS: flavone synthase; LAR: leucocyanidin reductase; ANR: anthocyanin reductase; UFGT: UDP glucose-flavonoid 3-O-glcosyl-transferase; MT: methyltransferase.

Compared to Shanhua15, 24 upregulated and 10 downregulated genes were identified in Zhonghua12 at the R4 stage, indicating that flavonoids and anthocyanidins biosynthesis pathways were enhanced by upregulated gene expression in Zhonghua12 testa. Genes PAL, C4H, CHS, F3H, F3′H, DFR, ANS, ANR, LAR, and FLS were all significantly upregulated in Zhonghua12. Only five 4CL genes (*arahy.1KSV8R*, *arahy.DK6AU1*, *arahy.ST1ALI*, *arahy.WI4YKF*, and *arahy. X2F5F9*), four CHI genes (*arahy.2GDU51*, *arahy.425ZXH*, *arahy.6JHV2K*, and *arahy.LGAM8W*), and FNS gene (*arahy.Q2BYIT*) were downregulated. Interestingly, many related genes had no significant difference in expression at the R7 stage, including genes C4H, CHI, F3H, ANS, ANR, and LAR. Nine genes were detected to be upregulated at the R7 stage, including CHS, F3′H, DFR, FLS, FNS, and four 4CL genes (Table S5). Among these upregulated genes, CHS (*arahy. CM90T6*) was upregulated by 4.97-fold, and it catalyzes the transformation of p-coumaryl-coA to both naringenin chalcone and isoliquiritigenin. Then, isoliquiritigenin is finally catalyzed to formononetin 7-O-glucoside, and this may be the reason for its high accumulation in Z7 testa of Zhonghua12. The F3′H genes (*arahy. 8F7PE4* and *arahy. K8H9R8*) catalyze the conversion of dihydrotricetin to dihydromyricetin and finally to petunidin 3-O-glucoside, and they have high expression in Z4 testa. The DFR enzyme catalyzes different types of substrates to synthesize leucodelphinidin and leucocyanidin, and the corresponding genes (*arahy. LDV9QN* and *arahy. X8EVF3*) showed 7.48- and 1.71-fold increments in Z4 compared to S4 (Figure 4, Table S5).

### 3.5. Transcription Factors Involved in Flavonoids and Anthocyanin Biosynthesis

Except for functional genes, transcription factors (TFs) play an important role in regulating most metabolic pathways. Studies have shown that the biosynthesis of flavonoid and anthocyanin is regulated by three types of TFs, including R2R3-MYB factor, basic helix-loop-helix (bHLH) proteins, and WD40 proteins [18]. Compared with Shanhua15, 425 and 237 TFs genes were found to be significantly changed in Zhonghua12 testa at R4 and R7 stages, respectively. Meanwhile, compared with R4, 354 differentially expressed TFs genes were detected in Shanhua15 at the R7 stage and 671 for Zhonghua12 (Table 2, Table S6).

MYB and bHLH have been confirmed to play important roles in regulating the expression of genes involved in flavonoid and anthocyanin biosynthesis in many plants [28–30]. In the S4-vs-Z4 group, 51 MYB TF genes were significantly changed, among which 35 genes were upregulated and 16 were downregulated in Zhonghua12. In the S7-vs-Z7 group, 30 MYB TF genes were differentially expressed in Zhonghua12, including 18 upregulated and 12 downregulated genes. As the seed developing from R4 to R7, 46 (10-up and 36-down) and 70 (15-up and 55-down) MYB TF genes were significantly changed in Shanhua15 and Zhonghua12 testa, respectively (Table 2). Moreover, 41 bHLH genes showed expression differences at the R4 stage, including 24 highly upregulated and 17 downregulated genes in Zhonghua12, while 29 bHLH DEGs were identified at the R7 stage, including 10 upregulated and 19 downregulated genes. With the development of peanut testa in one cultivar, 37 bHLH DEGs were identified in Shanhua 15, of which 8 were highly upregulated at the R7 stage, while 59 bHLH DEGs were detected in Zhonghua12, including 15 upregulated and 44 downregulated genes at the R7 stage (Table 2).

**Figure 5:**
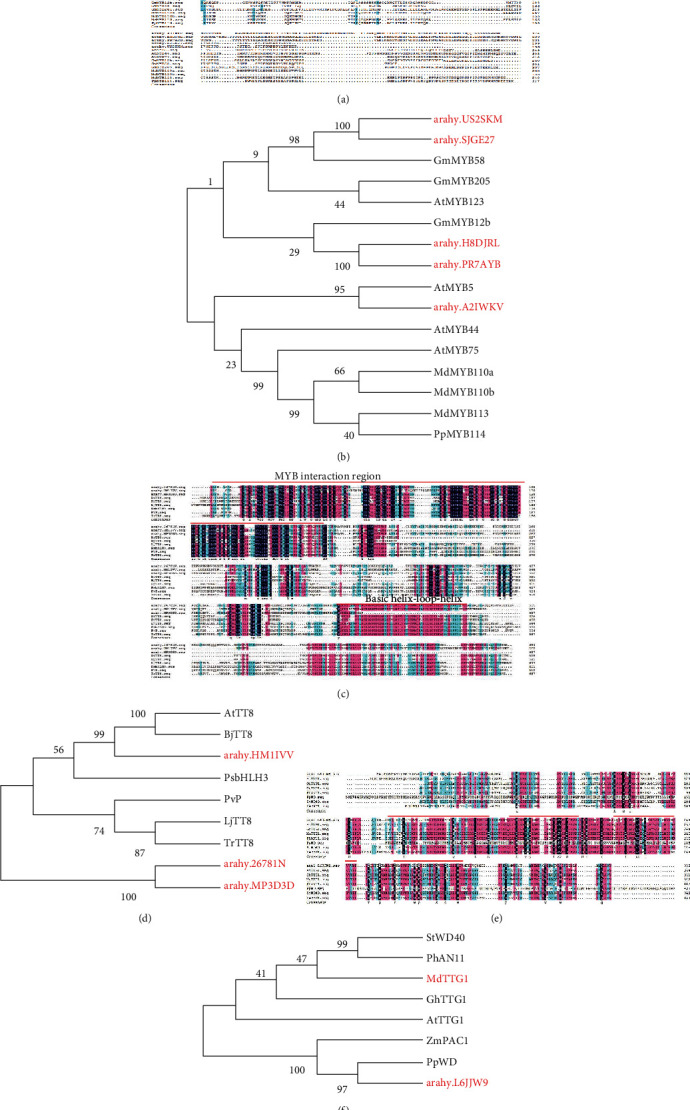
Phylogenetic analysis of MYBs, bHLHs, WD40, and their homologous proteins. (a, c, e) Phylogenetic analyses of peanut MYBs bHLHs and WD40 in other plants. (b, d, f) Multiple alignments of deduced amino acid sequences of peanut MYBs bHLHs and WD40 proteins with other functionally characterized MYBs bHLHs and WD40. R2R3 motif, C-terminal basic-helix1-loop-helix2, and WD-repeat domains were indicated at the top.

We further recruited five R2R3-MYBs, three bHLH, and one WD40 DEGs (Figure 5). The five MYBs-genes *arahy.A2IWKV*, *arahy.US2SKM*, *arahy.SJGE27*, *arahy.H8DJRL*, and *arahy.PR7AYB* were annotated in seed coat development, anthocyanin regulatory, and flavonol biosynthetic process. The expression of all five genes was highly upregulated at the R4 stage in Zhonghua12. Protein sequence comparison revealed that *arahy.US2SKM* and *arahy.SJGE27* was homologous (99%) to GmMYB58 from *Glycine max*, *arahy*.*H8DJRL*, and *arahy.PR7AYB* clustered together with *Glycine max* GmMYB12b, while *arahy.A2IWKV* was closely related to *Arabidopsis thaliana* AtMYB5 (Figure 5(b)). Protein sequence alignment showed five genes were identified as typical R2R3 MYB proteins (Figure 5(a)), which might play an important role in peanut testa pigmentation regulation. The bHLH gene *arahy.HM1IVV* was clustered with AtTT8 and BjTT8 (Figure 5(d)). Three bHLH genes contain N-terminal MYB interaction region, while *arahy.26781* N and *arahy.HM1IVV* contain a basic helix1-loop-helix (Figure 5(c)). The protein sequence of WD40 gene-*arahy.L6JJW9* had a higher sequence similarity to *Physcomitrella patens* PpWD (Figure 5(f)), which contained four highly conserved WD-repeat motifs (Figure 5(e)). The transcript showed that three types of TF genes have high expression levels at the R4 stage of seed development, resembling the expression pattern of most structural genes. That indicated MYB forms a ternary complex with bHLH and WD40 to activate genes related to anthocyanin biosynthesis.

### 3.6. qRT-PCR Validation of Gene Expression

**Figure 6:**
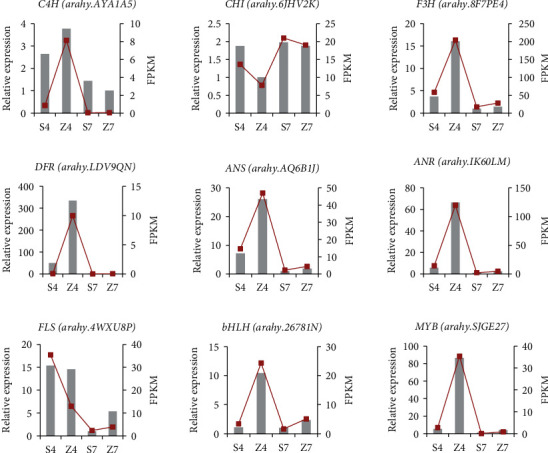
qRT-PCR verification of DEGs between S4, Z4, S7, and Z7. Transcript levels of 9 genes, which were involved in flavonoid and anthocyanin biosynthesis. The *y*-axis shows the relative gene expression levels analyzed by qRT-PCR and RNA-Seq. The *x*-axis shows the testa sample of developmental stages. In this figure, gray columns correspond with expression data of qRT-PCR, while red lines denote data of RNA-Seq. S4, Shanhua15 R4 pod period; Z4, Zhonghua12 R4 pod period; S7, Shanhua15 R7 pod period; Z7, Zhonghua12 R7 pod period.

A total of nine DEGs (Table S1) involved in flavonoid biosynthesis, seven functional genes, and two TF genes were randomly selected for qRT-PCR validation. As shown in Figure 6, the relative gene expression of nine test genes was all consistent with the RNA-seq results; thus, we believe that the transcriptome data is of high accuracy and reliability.

## 4. Discussion

Peanut contains many important nutrients, and the testa color affects its nutritional and commercial value. The antioxidant capacity of peanut testa is much higher than that of cotyledons [31]. For example, proanthocyanidin B is widely distributed in peanut testa, and it is a novel allosteric AKT inhibitor, which not only has antioxidant and anti-inflammatory properties but also has a strong antitumor efficacy [32]. In recent years, consumers prefer peanuts with dark testa due to their health benefits from the higher content of anthocyanins and other flavonoids [33]. In addition, aflatoxin resistance is also related to the biosynthesis pathway of flavonoids and phenylpropyl [34]. At present, the testa of most peanut varieties on the market is pink, and some varieties are red. Previous studies on peanut testa pigmentation were limited to genome and transcriptome level, for example, the differences of testa transcriptome in three growth stages of pink peanut varieties were analyzed by RNA-Seq [21]. In this paper, the combined analysis of transcriptome and metabolome provided a new approach to reveal the molecular mechanism of pigment formation in peanut testa.

The anthocyanin content and composition are important factors for different colors of plant tissues or organs. We detected the total anthocyanin content of testa of two peanut varieties during seed development, and the results showed that the total anthocyanin content of two varieties both increased from R2 to R7 stage, suggesting that testa pigments gradually accumulated with seed development. Moreover, the total anthocyanin content of red testa variety Zhonghua 12 was significantly higher than that of pink testa variety Shanhua 15 (Figure 1). Anthocyanins are considered to be the most important pigments in most plants [35]. However, previous studies have shown that different plants have different anthocyanins for pigmentation. During the process of red pigment formation in jujube peel, delphinidin, malvidin 3-O-glucoside, and delphinidin 3-O-glucoside were considered to be the key anthocyanins [36], but cyanidin O-malonylhexoside was the first anthocyanin identified in purple mutant figure [12]. When comparing different asparagus varieties, peonidin, cyanidin, and their glycoside derivatives were the major anthocyanins [1]. The strong red color of longan was due to the abundant contents of cyanidin 3-O-glucoside, cyanidin 3-O-6^″^-malonyl-glucoside, and cyanidin O-syringic acid found in its pericarp [37]. In order to clarify the pigmentation mechanism of peanut testa and analyze the metabolite basis of the difference between pink and red testa, the composition and content of testa metabolites at the R4 and R7 stage of Shanhua 15 and Zhonghua 12 were determined using the UPLC-MS-/MS-based approach. The metabolome analysis showed that a total of 198 flavonoid-related SCMs were identified, and the main pigment components of peanut red testa were petunidin 3-O-glucoside and cyanidin O-acetylhexoside. This study further illustrates the primary anthocyanins that affect seed color in plant are petunidin derivatives and cyanidin derivatives.

Flavone plays an important role in the yellow pigment formation of many plants [38, 39]. Interestingly, the contents of tricin O-malonylhexoside, naringenin C-hexoside, and apigenin 7-O-glucoside were extremely higher in the Shanhua15 S7 sample, and they were all over 1.00 + E04 times higher than that of Zhonghua12 Z7, especially for tricin O-malonylhexoside, which was more than 1.00 + E06 times higher. Then, we noticed that the endotesta of Shanhua15 was yellow, while it was white in Zhonghua12, suggesting that high content of Tricin O-malonylhexoside, naringenin C-hexoside, and apigenin 7-O-glucoside maybe give a yellow coloration in peanut endotesta. In this study, changes of many other metabolites in peanut testa were also investigated, many of which were not directly related to testa pigmentation, but correlated with human health. For example, formononetin 7-O-glucoside (Ononin) can inhibit the proliferation of tumor cells and promote apoptosis and reduce cell invasion and migration [40], and its content was significantly higher in Zhonghua12 testa with more than 2000-fold than Shanhua15. Besides, persicoside content was very high at both the R4 and R7 stages of Zhonghua12 testa, and it has potential radical scavenging activity [41]. To sum up, peanut with red testa contains more ingredients that are beneficial to human health.

**Figure 7:**
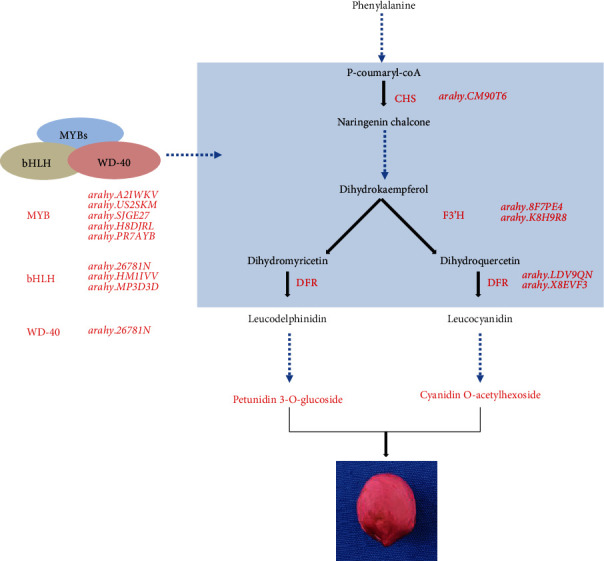
Prediction new model of red peanut testa formation. The red label indicated upregulated genes or metabolites. CHS: chalcone synthase; F3′H: flavanoid 3′-hydroxylase; DFR: dihydroflavonol 4-reductase.

Based on the identification of SCMs, the gene expression profile of peanut testa was performed between two varieties at R4 and R7 stages. Compared with Shanhua 15 S4 and S7, thousands of significant DEGs were identified in Zhonghua 12, and many of them were enriched in the secondary metabolite biosynthetic process and flavonoid biosynthesis pathway through GO and KEGG analysis, suggesting that these genes are likely to be related to pigmentation in pink and red testa. The pigment of peanut testa accumulated gradually with seed development, and the coloring was basically completed at the R7 stage. It was found that the genes involved in flavonoid and anthocyanidin biosynthesis, PAL, C4H, CHS, F3H, F3′H, DFR, ANS, ANR, LAR, and FLS, were significantly upregulated in Zhonghua12 testa at the R4 stage, but most of them were not significantly changed at the R7 stage. These results suggested that changes in gene expression occurred much earlier than phenotypic changes. In the early stage of peanut seed development, although the seed coat has not been completely stained, the genes related to flavonoid biosynthesis have been actively expressed, similar to studies on blueberry and pear that genes involved in anthocyanin and flavonoid synthesis were also highly expressed in the early stages of fruit development [42, 43]. In addition, affected by the active expression of related genes, a large number of flavonoid molecules have been accumulated in the white-period of jujube peel, and with the fruit ripening, the early actively expressed genes gradually become silenced [36]. Therefore, genes PAL, C4H, F3H, F3′H, DFR, ANS, ANR, LAR, and FLS are likely to play important roles in the early anthocyanin accumulation. Besides, CHS has been verified to be related to the red coloration in crabapple varieties [44], and it was significantly inhibited in white-flowered mustards, while other genes in the anthocyanin biosynthesis pathway were not significantly different from colored individuals [45]. The high expression of CHS was also related to higher anthocyanin content in peach, and the results were also verified in 30 peach varieties [24]. In our study, CHS (*arahy. CM90T6*) had higher expression in Zhonghua12 and was upregulated by 4.97-fold in Z7, indicating that the CHS gene may be related to testa pigmentation. The expression of F3′H genes determined which component of anthocyanin will be synthesized in grape and rose [46, 47]. In the present study, the F3′H genes (*arahy. 8F7PE4* and *arahy. K8H9R8*) have a high expression level in Z4 testa, which catalyze the conversion of dihydrotricetin to dihydromyricetin or dihydrokaempferol to dihydroquercetin. The DFR enzyme can catalyze different types of substrates to synthesize leucodelphinidin and leucocyanidin. DFR genes (*arahy. LDV9QN* and *arahy. X8EVF3*) showed 7.48- and 1.71-fold increments in Zhonghua12 compared to Shanhua15 in the R4 period. These DEGs may be the critical factor for the high accumulation of dihydromyricetin, cyanidin O-acetylhexoside, and petunidin 3-O-glucoside in Zhonghua12 (Figure 7).

In addition to those functional genes, TFs have also been confirmed to play a critical role in the regulation of anthocyanin biosynthesis, including MYB, bHLH, and WD40 [44, 48]. For example, MdMYB1 and MdMYB3 were the main regulators for anthocyanin biosynthesis and fruit coloring in apple [49, 50], and PpMYB10 and PpMYB114 regulate anthocyanin biosynthesis in pear [38]. In peanut, an R2R3-MYB gene was identified previously, regulating purple testa formation [19]. In the present study, many MYBs were detected to be differentially expressed between Shanhua 15 and Zhonghua 12 (Table S5). Among them, five MYB genes were annotated in seed coat development, anthocyanin regulatory, and flavonol biosynthetic process. The expression of all five genes was highly upregulated at the R4 stage in Zhonghua12. Protein sequence comparison revealed that the genes clustered together with GmMYB58, AtMYB5, and GmMYB12b, which have a major role in regulating testa developmental process in *Glycine max* and *Arabidopsis thaliana* [51–52]. The MYBs had similar expression patterns to structural genes, and their expression trends were closely related to the contents of petunidin 3-O-glucoside and cyanidin O-acetylhexoside. We also identified three differentially expressed bHLH (*arahy.26781*N, *arahy.HM1IVV*, and *arahy.MP3D3D*) and WD40 (*arahy.L6JJW9*) transcription factor genes that may be involved in anthocyanin biosynthesis in peanut testa. The expression patterns of MYB, bHLH, and WD40 was consistent with flavonoid synthesis DEGs (Figure 7). This implies that control of the biosynthesis of the pigments in peanut testa may occur through transcriptional regulation by the MBW complex. The detailed functional analysis needs further verification.

## 5. Conclusions

In summary, flavonoid metabolites and related genes in pink and red testa of peanut were identified through combined transcriptome and metabolome analysis. The accumulation of petunidin 3-O-glucoside and cyanidin O-acetylhexoside in the late stage of seed development is the main reason for the red testa appearance. In addition, the genes PAL, C4H, CHS, F3H, F3′H, DFR, ANS, LAR, and ANR in the flavonoid biosynthesis pathway were highly expressed in the early stage, playing an important role in anthocyanin accumulation. CHS gene *arahy.CM90T6*, F3′H genes (*arahy. 8F7PE4* and *arahy. K8H9R8*), and DFR genes (*arahy. LDV9QN* and *arahy. X8EVF3*) may be the key functional genes controlling the formation of pink and red testa in peanut. Transcriptomic analysis indicated MYB, bHLH, and WD40 in the biosynthetic pathway of anthocyanin may be the key regulatory genes in pink and red pigment formation. This is a comprehensive analysis on flavonoid metabolites and related genes expression in peanut testa, providing a reference for revealing the regulatory mechanism of pigment accumulation in peanut testa.

## Figures and Tables

**Table 1:** Significantly enriched KEGG pathways between Shanhua15 and Zhonghua12 peanuts testa.

No.	Pathway	DEGs with pathway annotation	All genes with pathway annotation	*P* value	Corrected *P* value	Pathway ID
S4 vs. Z4						
1	Biosynthesis of secondary metabolites	377	2759	2.7E-09	7.5 E-07	ko01110
2	Flavonoid biosynthesis	43	162	6.8E-09	1.9E-06	ko00941
3	Cell cycle–yeast	38	152	2.8E-07	7.8E-05	ko04111

S7 vs. Z7						
1	Flavonoid biosynthesis	36	162	4.7E-13	1.3E-10	ko00941
2	Circadian rhythm–plant	36	162	4.7E-13	1.3E-10	ko04712
3	Biosynthesis of secondary metabolites	213	2759	6.1E-10	1.6E-07	ko01110
4	Phenylpropanoid biosynthesis	50	452	6.1E-07	0.00016	ko00940

S4 vs. S7						
1	Metabolic pathways	503	4863	5.9E-17	1.6E-14	ko01100
2	Biosynthesis of secondary metabolites	314	2759	1.4E-11	3.8E-09	ko01110
3	Flavonoid biosynthesis	38	162	4.9E-10	1.3E-07	ko00941

Z4 vs. Z7						
1	Biosynthesis of secondary metabolites	551	2759	0	0	ko01110
2	Metabolic pathways	837	4863	9.6E-10	2.8E-07	ko01100
3	Photosynthesis–antenna proteins	18	28	3.1E-09	9.1E-07	ko00196

S4, Shanhua15 R4 pod period; Z4, Zhonghua12 R4 pod period; S7, Shanhua15 R7 pod period; Z7, Zhonghua12 R7 pod period. Significant pathways were identified by corrected *P* ≤ 0.01.

**Table 2:** Differentially expressed transcription factors in the testa of R4 and R7 stages of Shanhua15 and Zhonghua12 peanuts.

Comparison group	Gene name	Number of DEGs	Upregulated DEGs	Downregulated DEGs	Biological functions
S4 vs. S7	MYB	46	10	36	Cell development and anthocyanin pathway
	bHLH	37	8	29	Plant development and substance metabolism
	Other TFs	262	67	195	
	In total	345	85	260	

S7 vs. Z7	MYB	30	18	12	Cell development and anthocyanin pathway
	bHLH	29	10	19	Plant development and substance metabolism
	Other TFs	178	85	93	
	In total	237	113	124	

S4 vs. Z4	MYB	51	35	16	Cell development and anthocyanin pathway
	bHLH	41	24	17	Plant development and substance metabolism
	Other TFs	333	157	176	
	In total	425	216	209	

Z4 vs. Z7	MYB	70	15	55	Cell development and anthocyanin pathway
	bHLH	59	15	44	Plant development and substance metabolism
	Other TFs	542	207	335	
	In total	671	237	434	

S4, Shanhua15 R4 pod period; Z4, Zhonghua12 R4 pod period; S7, Shanhua15 R7 pod period; Z7, Zhonghua12 R7 pod period. Differentially expressed genes were identified by log2 (fold change) ≥1 and FDR ≤0.05.

## Data Availability

The data used to support the findings of this study are included within the article. The data used to support the findings of this study are included within the supplementary information files.

## Supplementary Materials

Supplementary Materials Supplementary Figure 1: GO classification of differentially expressed genes of R7 stage of Shanhua15 and Zhonghua12 testa. Supplementary Figure 2: COG functional classification of differentially expressed genes of R4 and R7 stages of Shanhua15 and Zhonghua12 testa. S4, Shanhua15 R4 pod period; Z4, Zhonghua12 R4 pod period; S7, Shanhua15 R7 pod period; Z7, Zhonghua12 R7 pod period. Supplementary Table 1: primers for qRT-PCR verification. Supplementary Table 2: significantly changed metabolites between Shanhua15 and Zhonghua12 testa. Supplementary Table 3: summary of the sequencing and de novo assembly. Supplementary Table 4: statistics of GO Enrichment in the testa of R4 and R7 stages of Shanhua15 and Zhonghua12 peanut. Supplementary Table 5: DEGs of flavonoid and anthocyanin biosynthesis in the testa of R4 and R7 stages of Shanhua15 and Zhonghua12 peanut. Supplementary Table 6: differentially expressed transcription factors in the testa of R4 and R7 stages of Shanhua15 and Zhonghua12 peanut.
