# Supplementary material for: Transcriptome and Metabolome Analysis Unveil Anthocyanin Metabolism in Pink and Red Testa of Peanut (Arachis hypogaea L.)
Source: Int J Genomics. 2021 Aug 6;2021:5883901. doi: 10.1155/2021/5883901 (PMC8363441; doi:10.1155/2021/5883901)
Supplement: Supplementary Materials — Supplementary Figure 1: GO classification of differentially expressed genes of R7 stage of Shanhua15 and Zhonghua12 testa. Supplementary Figure 2: COG functional classification of differentially expressed genes of R4 and R7 stages of Shanhua15 and Zhonghua12 testa. S4, Shanhua15 R4 pod period; Z4, Zhonghua12 R4 pod period; S7, Shanhua15 R7 pod period; Z7, Zhonghua12 R7 pod period. Supplementary Table 1: primers for qRT-PCR verification. Supplementary Table 2: significantly changed metabolites between Shanhua15 and Zhonghua12 testa. Supplementary Table 3: summary of the sequencing and de novo assembly. Supplementary Table 4: statistics of GO Enrichment in the testa of R4 and R7 stages of Shanhua15 and Zhonghua12 peanut. Supplementary Table 5: DEGs of flavonoid and anthocyanin biosynthesis in the testa of R4 and R7 stages of Shanhua15 and Zhonghua12 peanut. Supplementary Table 6: differentially expressed transcription factors in the testa of R4 and R7 stages of Shanhua15 and Zhonghua12 peanut. [file 5883901.f1.zip › 5883901.f1/Table S2.pdf]

Table S2 Significantly changed metabolites between Shanhua15 and Zhonghua12

Index	Compounds	Class	Shanhua15	Zhonghua12
pmb2959	Cyanidin O-acetylhexoside	Anthocyanins	6.89E+03	1.01E+05
pme1773	Cyanidin 3-O-rutinoside (Keracyanin)	Anthocyanins	4.63E+05	9.00E+00
pme1793	Pelargonin	Anthocyanins	4.38E+04	1.07E+04
pme3391	Petunidin 3-O-glucoside	Anthocyanins	5.19E+05	7.91E+06
pmf0263	Apigeninidin chloride	Anthocyanins	4.08E+03	2.87E+04
pmb2975	Hesperetin O-Glucuronic acid	Flavanone	9.00E+00	1.87E+04
pme0371	Naringenin 7-O-glucoside (Prunin)	Flavanone	5.95E+05	9.09E+04
pme2979	Pinocembrin (Dihydrochrysin)	Flavanone	9.32E+03	6.36E+04
pme3464	Isosakuranetin (4'-Methylnaringenin)	Flavanone	6.57E+03	6.81E+04
pma0724	Naringenin C-hexoside	Flavone	2.15E+05	9.00E+00
pma6199	Chrysin O-hexoside	Flavone	1.59E+06	3.05E+05
pma6558	Velutin	Flavone	2.35E+04	2.54E+05
pmb0566	Luteolin O-hexosyl-O-pentoside	Flavone	1.91E+05	9.27E+03
pmb0580	Chrysin 5-O-glucoside (Toringin)	Flavone	1.66E+06	2.93E+05
pmb0605	Apigenin 7-O-glucoside (Cosmosiin)	Flavone	1.22E+05	9.00E+00
pmb0607	Chrysoeriol 7-O-hexoside	Flavone	1.10E+07	1.37E+06
pmb0615	esperetin C-hexosyl-O-hexosyl-O-hexosi	Flavone	3.96E+04	4.50E+05
pmb0618	8-C-hexosyl-hesperetin O-hexoside	Flavone	9.00E+00	1.28E+06
pmb0645	6-C-hexosyl-hesperetin O-hexoside	Flavone	9.54E+04	2.54E+06
pmb0663	8-C-hexosyl-luteolin O-hexoside	Flavone	4.37E+05	9.81E+06
pmb0689	Chrysoeriol C-hexoside	Flavone	2.99E+06	1.24E+05
pmb0712	Tricin 5-O-hexosyl-O-hexoside	Flavone	5.23E+05	6.06E+06
pmb0713	Tricin 7-O-hexosyl-O-hexoside	Flavone	6.46E+05	7.80E+06
pmb0720	Tricin O-malonylhexoside	Flavone	9.57E+06	9.00E+00
pmb0724	Tricin O-rhamnoside	Flavone	2.09E+05	6.97E+03
pmb2954	uteolin O-hexosyl-O-hexosyl-O-hexosic	Flavone	1.87E+04	2.62E+05
pmb3000	Chrysoeriol O-acetylhexoside	Flavone	3.07E+05	1.99E+06
pmb3046	Tricin 7-O-hexoside	Flavone	2.71E+05	4.88E+06
pmb3052	Tricin 4'-O-β-guaiacylglycerol	Flavone	9.06E+03	3.16E+03
pme2457	Luteolin 7-O-glucoside (Cynaroside)	Flavone	1.20E+06	1.48E+05
pme1667	Isovitexin 7-O-glucoside (Saponarin)	Flavonoid	5.88E+04	9.00E+00
pmf0246	Orientin	Flavonoid	5.65E+04	1.63E+04
pmf0369	Persicoside	Flavonoid	9.00E+00	2.37E+05
pmf0371	Pedalitin	Flavonoid	4.28E+05	3.22E+06
pmf0382	5,7-Dihydroxy-3',4',5'-trimethoxyflavone	Flavonoid	1.09E+04	7.58E+04
pma0214	methylQuercetin O-hexoside	Flavonol	5.71E+05	1.30E+07
pmb0565	Syringetin 3-O-hexoside	Flavonol	1.68E+05	7.62E+05
pmb0706	Quercetin 5-O-malonylhexosyl-hexoside	Flavonol	8.37E+04	6.71E+06
pmb0709	Quercetin 7-O-malonylhexosyl-hexoside	Flavonol	1.64E+05	7.66E+06
pmb3026	Quercetin O-acetylhexoside	Flavonol	5.48E+05	2.01E+07
pme0196	Kaempferol	Flavonol	1.11E+04	8.44E+04
pme0197	Quercetin 3-O-rutinoside (Rutin)	Flavonol	1.85E+05	1.09E+06
pme1500	Kumatakenin	Flavonol	9.12E+03	1.01E+05
pme1622	Kaempferol 3-O-glucoside (Astragalin)	Flavonol	1.44E+06	2.57E+05
pme2895	Dihydromyricetin	Flavonol	9.00E+00	2.67E+05
pme3369	Rhamnetin (7-O-methxyl quercetin)	Flavonol	8.34E+03	5.83E+04
pme3442	Quercetin 7-O-β-D-Glucuronide	Flavonol	4.74E+05	2.92E+06
pme3484	Myricetin 3-O-galactoside	Flavonol	2.14E+03	2.69E+04
pme3230	Calycosin	Isoflavone	7.79E+05	8.20E+04

pme3251	Glycitein	Isoflavone	7.97E+05	8.31E+04
pme3276	2'-Hydroxygenistein	Isoflavone	2.46E+05	9.47E+04
pme3502	Formononetin 7-O-glucoside (Ononin)	Isoflavone	9.00E+00	2.02E+04
pmb2947	Catechin-catechin-catechin	Polyphenol	5.50E+06	5.94E+05
pmb3114	Epicatechin-epiafzelechin	Polyphenol	5.05E+05	7.70E+04
pme1486	Epigallate catechin gallate (EGCG)	Polyphenol	2.88E+04	9.00E+00
pme1516	Epigallocatechin (EGC)	Polyphenol	6.91E+05	5.38E+06
pme1535	(+)-Gallocatechin (GC)	Polyphenol	1.35E+05	1.72E+04
pme1562	Epicatechin gallate (ECG)	Polyphenol	1.43E+04	1.53E+03
pme2246	Ellagic acid	Polyphenol	9.00E+00	4.27E+04
pmf0345	(-)-Epiafzelechin	Polyphenol	3.39E+05	4.05E+04
pmf0379	Gallocatechin gallate	Polyphenol	3.28E+04	9.00E+00
pme0434	Procyanidin B2	Proanthocyanidins	1.07E+06	1.22E+05
pme0436	Procyanidin B3	Proanthocyanidins	1.10E+08	1.48E+07

testa

VIP	Fold_Change	LogFC	Regulate
1.27E+00	1.47E+01	3.88E+00	up
2.56E+00	1.95E-05	-1.56E+01	down
1.12E+00	2.44E-01	-2.03E+00	down
1.11E+00	1.52E+01	3.93E+00	up
1.07E+00	7.03E+00	2.81E+00	up
2.15E+00	2.07E+03	1.10E+01	up
1.07E+00	1.53E-01	-2.71E+00	down
1.05E+00	6.83E+00	2.77E+00	up
1.20E+00	1.04E+01	3.37E+00	up
2.47E+00	4.19E-05	-1.45E+01	down
1.03E+00	1.92E-01	-2.38E+00	down
1.17E+00	1.08E+01	3.43E+00	up
1.88E+00	4.85E-02	-4.37E+00	down
1.30E+00	1.77E-01	-2.50E+00	down
1.69E+00	7.40E-05	-1.37E+01	down
1.13E+00	1.24E-01	-3.01E+00	down
1.15E+00	1.14E+01	3.51E+00	up
2.66E+00	1.42E+05	1.71E+01	up
1.40E+00	2.67E+01	4.74E+00	up
1.30E+00	2.25E+01	4.49E+00	up
1.58E+00	4.16E-02	-4.59E+00	down
1.07E+00	1.16E+01	3.53E+00	up
1.09E+00	1.21E+01	3.59E+00	up
2.90E+00	9.40E-07	-2.00E+01	down
1.93E+00	3.34E-02	-4.91E+00	down
1.20E+00	1.40E+01	3.81E+00	up
1.06E+00	6.48E+00	2.70E+00	up
1.31E+00	1.80E+01	4.17E+00	up
1.40E+00	3.49E-01	-1.52E+00	down
1.12E+00	1.23E-01	-3.02E+00	down
2.31E+00	1.53E-04	-1.27E+01	down
1.08E+00	2.88E-01	-1.80E+00	down
2.48E+00	2.63E+04	1.47E+01	up
1.10E+00	7.52E+00	2.91E+00	up
1.08E+00	6.93E+00	2.79E+00	up
1.28E+00	2.27E+01	4.50E+00	up
1.16E+00	4.53E+00	2.18E+00	up
1.60E+00	8.02E+01	6.33E+00	up
1.50E+00	4.66E+01	5.54E+00	up
1.45E+00	3.66E+01	5.19E+00	up
1.26E+00	7.62E+00	2.93E+00	up
1.04E+00	5.89E+00	2.56E+00	up
1.17E+00	1.11E+01	3.47E+00	up
1.02E+00	1.78E-01	-2.49E+00	down
2.49E+00	2.96E+04	1.49E+01	up
1.07E+00	6.99E+00	2.81E+00	up
1.04E+00	6.16E+00	2.62E+00	up
1.68E+00	1.26E+01	3.65E+00	up
1.16E+00	1.05E-01	-3.25E+00	down

1.17E+00	1.04E-01	-3.26E+00	down
1.07E+00	3.84E-01	-1.38E+00	down
2.16E+00	2.24E+03	1.11E+01	up
1.16E+00	1.08E-01	-3.21E+00	down
1.07E+00	1.52E-01	-2.71E+00	down
1.49E+00	3.12E-04	-1.16E+01	down
1.09E+00	7.78E+00	2.96E+00	up
1.12E+00	1.27E-01	-2.98E+00	down
1.56E+00	1.07E-01	-3.23E+00	down
2.26E+00	4.75E+03	1.22E+01	up
1.32E+00	1.19E-01	-3.07E+00	down
1.51E+00	2.74E-04	-1.18E+01	down
1.15E+00	1.14E-01	-3.14E+00	down
1.10E+00	1.34E-01	-2.90E+00	down
