# Supplementary material for: Transcriptome and Metabolome Analysis Unveil Anthocyanin Metabolism in Pink and Red Testa of Peanut (Arachis hypogaea L.)
Source: Int J Genomics. 2021 Aug 6;2021:5883901. doi: 10.1155/2021/5883901 (PMC8363441; doi:10.1155/2021/5883901)
Supplement: Supplementary Materials — Supplementary Figure 1: GO classification of differentially expressed genes of R7 stage of Shanhua15 and Zhonghua12 testa. Supplementary Figure 2: COG functional classification of differentially expressed genes of R4 and R7 stages of Shanhua15 and Zhonghua12 testa. S4, Shanhua15 R4 pod period; Z4, Zhonghua12 R4 pod period; S7, Shanhua15 R7 pod period; Z7, Zhonghua12 R7 pod period. Supplementary Table 1: primers for qRT-PCR verification. Supplementary Table 2: significantly changed metabolites between Shanhua15 and Zhonghua12 testa. Supplementary Table 3: summary of the sequencing and de novo assembly. Supplementary Table 4: statistics of GO Enrichment in the testa of R4 and R7 stages of Shanhua15 and Zhonghua12 peanut. Supplementary Table 5: DEGs of flavonoid and anthocyanin biosynthesis in the testa of R4 and R7 stages of Shanhua15 and Zhonghua12 peanut. Supplementary Table 6: differentially expressed transcription factors in the testa of R4 and R7 stages of Shanhua15 and Zhonghua12 peanut. [file 5883901.f1.zip › 5883901.f1/Table S3.pdf]

Table S3 Summary of the sequencing and de novo assembl

Samples	raw_reads	clean_reads	Total mapped	clean_bases	error_rate	Q20
S4-1	55799260	55216442	46977288	8282466300	0.365	97
S4-2	52581942	51933798	43959566	7790069700	0.345	97.195
S4-3	64718080	64270062	54187442	9640509300	0.345	97.195
S7-1	59889128	59443644	51038882	8916546600	0.355	97.1
S7-2	61166972	60444520	51740942	9066678000	0.335	97.285
S7-3	58975466	58328988	49660617	8749348200	0.345	97.18
Z4-1	58275958	57771286	48838904	8665692900	0.365	96.975
Z4-2	58295876	57658526	48628565	8648778900	0.36	97.03
Z4-3	55297900	54401188	45260740	8160178200	0.33	97.415
Z7-1	51008880	50406664	43170287	7560999600	0.43	96.24
Z7-2	48025548	47570326	40555926	7135548900	0.365	96.97
Z7-3	49785996	48144990	40494280	7221748500	0.35	97.12

y

Q30	GC (%)
91.955	44.215
92.375	44.21
92.425	44.55
92.195	44.61
92.57	44.285
92.39	44.1
91.9	44.215
92.03	44.16
92.85	44.14
90.12	44.08
91.95	44.185
92.28	43.905
