# Supplementary material for: Transcriptome and Metabolome Analysis Unveil Anthocyanin Metabolism in Pink and Red Testa of Peanut (Arachis hypogaea L.)
Source: Int J Genomics. 2021 Aug 6;2021:5883901. doi: 10.1155/2021/5883901 (PMC8363441; doi:10.1155/2021/5883901)
Supplement: Supplementary Materials — Supplementary Figure 1: GO classification of differentially expressed genes of R7 stage of Shanhua15 and Zhonghua12 testa. Supplementary Figure 2: COG functional classification of differentially expressed genes of R4 and R7 stages of Shanhua15 and Zhonghua12 testa. S4, Shanhua15 R4 pod period; Z4, Zhonghua12 R4 pod period; S7, Shanhua15 R7 pod period; Z7, Zhonghua12 R7 pod period. Supplementary Table 1: primers for qRT-PCR verification. Supplementary Table 2: significantly changed metabolites between Shanhua15 and Zhonghua12 testa. Supplementary Table 3: summary of the sequencing and de novo assembly. Supplementary Table 4: statistics of GO Enrichment in the testa of R4 and R7 stages of Shanhua15 and Zhonghua12 peanut. Supplementary Table 5: DEGs of flavonoid and anthocyanin biosynthesis in the testa of R4 and R7 stages of Shanhua15 and Zhonghua12 peanut. Supplementary Table 6: differentially expressed transcription factors in the testa of R4 and R7 stages of Shanhua15 and Zhonghua12 peanut. [file 5883901.f1.zip › 5883901.f1/Table S4.pdf]

Table S4 Statistics of GO Enrichment in the testa of R4 and R7 stages of Shanhu

Ontology	ID	Descriptior	GeneRatio	BgRatio	pvalue	p.adjust	qvalue	geneID
Cellular co	GO:00425	MCM com	10/1904	16/28537	9.43E-09	3.05E-06	3.05E-06	arahy.Tifru
Cellular co	GO:00003	THO comp	9/1904	29/28537	7.57E-05	0.01223	0.01223	arahy.Tifru
Molecular	GO:00503	trihydroxy	26/1903	51/27490	2.71E-17	2.27E-14	2.14E-14	arahy.Tifru
Molecular	GO:00167	oxidoredu	51/1903	324/2749C	3.17E-08	1.32E-05	1.25E-05	arahy.Tifru
Molecular	GO:01024	scopolin b	12/1903	54/27490	0.000262	0.073035	0.068796	arahy.Tifru
Molecular	GO:00084	beta-gluco	24/1903	170/2749C	0.000684	0.115688	0.108973	arahy.Tifru
Molecular	GO:00506	flavin aden	32/1903	255/2749C	0.000819	0.115688	0.108973	arahy.Tifru
Molecular	GO:00101	IAA-amino	5/1903	12/27490	0.000829	0.115688	0.108973	arahy.Tifru
Molecular	GO:00044	N,N-dimetl	9/1903	42/27490	0.001981	0.221691	0.208824	arahy.Tifru
Molecular	GO:00159	glucosidas	27/1903	216/2749C	0.002119	0.221691	0.208824	arahy.Tifru
Molecular	GO:00151	amino acid	24/1903	186/2749C	0.002389	0.222141	0.209248	arahy.Tifru
Molecular	GO:00191	cytokinin d	6/1903	22/27490	0.003104	0.259779	0.244702	arahy.Tifru
Molecular	GO:00152	antiporter	37/1903	337/2749C	0.003767	0.265151	0.249762	arahy.Tifru
Molecular	GO:00166	oxidoredu	12/1903	73/27490	0.004159	0.265151	0.249762	arahy.Tifru
Molecular	GO:00511	sugar tran	19/1903	142/2749C	0.00432	0.265151	0.249762	arahy.Tifru
Molecular	GO:00037	transcripti	9/1903	47/27490	0.004435	0.265151	0.249762	arahy.Tifru
Molecular	GO:00048	aspartate-	4/1903	11/27490	0.00509	0.284046	0.26756	arahy.Tifru
Molecular	GO:00082	exopeptida	22/1903	180/2749C	0.006747	0.302199	0.284659	arahy.Tifru
Molecular	GO:00800	quercetin	18/1903	138/2749C	0.007007	0.302199	0.284659	arahy.Tifru
Molecular	GO:00162	4-coumara	4/1903	12/27490	0.007221	0.302199	0.284659	arahy.Tifru
Molecular	GO:00503	L-tryptoph	4/1903	12/27490	0.007221	0.302199	0.284659	arahy.Tifru
Molecular	GO:00705	L-tryptoph	4/1903	12/27490	0.007221	0.302199	0.284659	arahy.Tifru
Molecular	GO:00800	quercetin	18/1903	140/2749C	0.008111	0.32329	0.304527	arahy.Tifru
Molecular	GO:00046	transmem	42/1903	415/2749C	0.008857	0.335552	0.316077	arahy.Tifru
Molecular	GO:00102	(+)-abscisi	4/1903	13/27490	0.009865	0.335552	0.316077	arahy.Tifru
Molecular	GO:00464	allene-oxid	4/1903	13/27490	0.009865	0.335552	0.316077	arahy.Tifru
Molecular	GO:00166	cinnamoyl-	5/1903	20/27490	0.010199	0.335552	0.316077	arahy.Tifru
Molecular	GO:00152	symporter	36/1903	349/2749C	0.011018	0.335552	0.316077	arahy.Tifru
Molecular	GO:00040	alcohol del	8/1903	45/27490	0.011225	0.335552	0.316077	arahy.Tifru
Molecular	GO:00166	oxidoredu	8/1903	45/27490	0.011225	0.335552	0.316077	arahy.Tifru
Molecular	GO:00168	aldehyde-l	7/1903	37/27490	0.012364	0.341747	0.321912	arahy.Tifru
Molecular	GO:00054	monovaler	6/1903	29/27490	0.013048	0.341747	0.321912	arahy.Tifru
Molecular	GO:00153	potassium	6/1903	29/27490	0.013048	0.341747	0.321912	arahy.Tifru
Molecular	GO:01200	jasmonic a	4/1903	14/27490	0.013066	0.341747	0.321912	arahy.Tifru
Molecular	GO:00166	oxidoredu	16/1903	126/2749C	0.013518	0.342855	0.322956	arahy.Tifru
Molecular	GO:00152	drug trans	29/1903	275/2749C	0.015873	0.38735	0.364869	arahy.Tifru
Molecular	GO:00154	cation:cati	8/1903	48/27490	0.016371	0.38735	0.364869	arahy.Tifru
Molecular	GO:00166	oxidoredu	46/1903	481/2749C	0.01666	0.38735	0.364869	arahy.Tifru
Molecular	GO:00053	organic aci	33/1903	326/2749C	0.018547	0.39988	0.376671	arahy.Tifru
Molecular	GO:00469	carboxylic	33/1903	326/2749C	0.018547	0.39988	0.376671	arahy.Tifru
Molecular	GO:00081	primary an	5/1903	23/27490	0.018632	0.39988	0.376671	arahy.Tifru
Molecular	GO:00191	transmem	43/1903	449/2749C	0.019645	0.411074	0.387215	arahy.Tifru
Molecular	GO:00506	NADP bind	16/1903	132/2749C	0.020294	0.414296	0.390251	arahy.Tifru
Molecular	GO:00151	carbohydr	22/1903	200/2749C	0.021269	0.423853	0.399253	arahy.Tifru
Molecular	GO:00166	oxidoredu	10/1903	70/27490	0.021843	0.425168	0.400491	arahy.Tifru
Molecular	GO:00046	calmoduli	9/1903	62/27490	0.026195	0.453219	0.426914	arahy.Tifru
Molecular	GO:00053	galactose t	4/1903	17/27490	0.026323	0.453219	0.426914	arahy.Tifru
Molecular	GO:00166	oxidoredu	4/1903	17/27490	0.026323	0.453219	0.426914	arahy.Tifru

Molecular	GO:00150	proton tra	37/1903	384/27490	0.026459	0.453219	0.426914	arahy.Tifru
Molecular	GO:00051	structural	3/1903	10/27490	0.0275	0.453219	0.426914	arahy.Tifru
Molecular	GO:00228	potassium	6/1903	34/27490	0.027552	0.453219	0.426914	arahy.Tifru
Molecular	GO:00041	aminopept	9/1903	63/27490	0.02875	0.453219	0.426914	arahy.Tifru
Molecular	GO:00011	transcripti	20/1903	184/27490	0.030291	0.453219	0.426914	arahy.Tifru
Molecular	GO:00011	transcripti	20/1903	184/27490	0.030291	0.453219	0.426914	arahy.Tifru
Molecular	GO:00164	CoA-ligase	8/1903	54/27490	0.031398	0.453219	0.426914	arahy.Tifru
Molecular	GO:00052	voltage-ga	9/1903	64/27490	0.031475	0.453219	0.426914	arahy.Tifru
Molecular	GO:00038	AMP deam	3/1903	11/27490	0.035904	0.453219	0.426914	arahy.Tifru
Molecular	GO:00040	adenylylsu	3/1903	11/27490	0.035904	0.453219	0.426914	arahy.Tifru
Molecular	GO:00082	tripeptidyl	3/1903	11/27490	0.035904	0.453219	0.426914	arahy.Tifru
Molecular	GO:00476	adenosine-	3/1903	11/27490	0.035904	0.453219	0.426914	arahy.Tifru
Molecular	GO:19901	plant seed	3/1903	11/27490	0.035904	0.453219	0.426914	arahy.Tifru
Molecular	GO:00150	potassium	17/1903	153/27490	0.036187	0.453219	0.426914	arahy.Tifru
Molecular	GO:00099	calcium-de	9/1903	66/27490	0.037451	0.453219	0.426914	arahy.Tifru
Molecular	GO:00108	calcium-de	9/1903	66/27490	0.037451	0.453219	0.426914	arahy.Tifru
Molecular	GO:00305	cyclic nucl	7/1903	46/27490	0.037578	0.453219	0.426914	arahy.Tifru
Molecular	GO:00305	cAMP bind	7/1903	46/27490	0.037578	0.453219	0.426914	arahy.Tifru
Molecular	GO:00305	cGMP bind	7/1903	46/27490	0.037578	0.453219	0.426914	arahy.Tifru
Molecular	GO:00039	acetyl-CoA	4/1903	19/27490	0.038419	0.453219	0.426914	arahy.Tifru
Molecular	GO:01030	indole-3-p	4/1903	19/27490	0.038419	0.453219	0.426914	arahy.Tifru
Molecular	GO:00151	monosacch	13/1903	110/27490	0.040661	0.453219	0.426914	arahy.Tifru
Molecular	GO:00055	macrolide	5/1903	28/27490	0.040942	0.453219	0.426914	arahy.Tifru
Molecular	GO:00055	FK506 binc	5/1903	28/27490	0.040942	0.453219	0.426914	arahy.Tifru
Molecular	GO:00053	carbohydra	15/1903	133/27490	0.042073	0.453219	0.426914	arahy.Tifru
Molecular	GO:00054	carbohydra	15/1903	133/27490	0.042073	0.453219	0.426914	arahy.Tifru
Molecular	GO:00512	dioxygenas	30/1903	312/27490	0.043076	0.453219	0.426914	arahy.Tifru
Molecular	GO:00995	ion antipor	11/1903	89/27490	0.043166	0.453219	0.426914	arahy.Tifru
Molecular	GO:00228	voltage-ga	9/1903	68/27490	0.044157	0.453219	0.426914	arahy.Tifru
Molecular	GO:00085	organic ani	38/1903	413/27490	0.045216	0.453219	0.426914	arahy.Tifru
Molecular	GO:00151	pentose tr	3/1903	12/27490	0.045465	0.453219	0.426914	arahy.Tifru
Molecular	GO:00151	D-xylose tr	3/1903	12/27490	0.045465	0.453219	0.426914	arahy.Tifru
Molecular	GO:00155	mannitol tı	3/1903	12/27490	0.045465	0.453219	0.426914	arahy.Tifru
Molecular	GO:00155	sorbitol tra	3/1903	12/27490	0.045465	0.453219	0.426914	arahy.Tifru
Molecular	GO:00155	D-ribose tr	3/1903	12/27490	0.045465	0.453219	0.426914	arahy.Tifru
Molecular	GO:00168	1-aminocy	3/1903	12/27490	0.045465	0.453219	0.426914	arahy.Tifru
Molecular	GO:00505	desacetoxy	4/1903	20/27490	0.045474	0.453219	0.426914	arahy.Tifru
Molecular	GO:00168	acid-thiol l	8/1903	58/27490	0.045484	0.453219	0.426914	arahy.Tifru
Molecular	GO:00198	isoprenoid	5/1903	29/27490	0.046753	0.46038	0.43366	arahy.Tifru
Molecular	GO:00426	ATPase act	40/1903	440/27490	0.047653	0.463785	0.436867	arahy.Tifru
Molecular	GO:00168	carbon-sul	8/1903	59/27490	0.049561	0.475417	0.447825	arahy.Tifru
Molecular	GO:00151	polyol tran	6/1903	39/27490	0.049984	0.475417	0.447825	arahy.Tifru
Molecular	GO:00154	P-P-bond-h	43/1903	481/27490	0.051659	0.475627	0.448022	arahy.Tifru
Molecular	GO:00041	aspartic-ty	13/1903	114/27490	0.051737	0.475627	0.448022	arahy.Tifru
Molecular	GO:00700	aspartic-ty	13/1903	114/27490	0.051737	0.475627	0.448022	arahy.Tifru
Molecular	GO:00169	oxidoreduc	18/1903	172/27490	0.052279	0.475627	0.448022	arahy.Tifru
Molecular	GO:01028	geraniol 1(	5/1903	30/27490	0.053028	0.477253	0.449553	arahy.Tifru
Molecular	GO:00152	solute:pro	17/1903	161/27490	0.054185	0.479523	0.451692	arahy.Tifru
Molecular	GO:00152	solute:cati	12/1903	104/27490	0.055734	0.479523	0.451692	arahy.Tifru
Molecular	GO:00153	primary ac	43/1903	484/27490	0.055972	0.479523	0.451692	arahy.Tifru

Molecular	GO:00338	ribonuclea	3/1903	13/27490	0.056145	0.479523	0.451692	arahy.Tifru
Molecular	GO:00465	mandeloni	3/1903	13/27490	0.056145	0.479523	0.451692	arahy.Tifru
Biological	GO:00062	DNA unwir	8/1795	11/26646	5.74E-08	0.000131	0.000126	arahy.Tifru
Biological	GO:00096	phenylpro	42/1795	297/2664	4.27E-06	0.003886	0.003742	arahy.Tifru
Biological	GO:00445	secondary	58/1795	467/2664	5.09E-06	0.003886	0.003742	arahy.Tifru
Biological	GO:00068	drug transı	22/1795	123/2664	2.31E-05	0.013221	0.012732	arahy.Tifru
Biological	GO:20000	regulation	13/1795	52/26646	2.99E-05	0.013691	0.013185	arahy.Tifru
Biological	GO:00098	auxin bios	18/1795	94/26646	4.92E-05	0.016743	0.016123	arahy.Tifru
Biological	GO:00550	monovaler	25/1795	157/2664	5.12E-05	0.016743	0.016123	arahy.Tifru
Biological	GO:00098	auxin meta	21/1795	129/2664	0.000144	0.04112	0.039598	arahy.Tifru
Biological	GO:00083	isoprenoid	9/1795	32/26646	0.00019	0.048328	0.04654	arahy.Tifru
Biological	GO:00325	DNA duple	8/1795	26/26646	0.000218	0.049829	0.047985	arahy.Tifru
Biological	GO:00436	regulation	5/1795	10/26646	0.000261	0.049911	0.048064	arahy.Tifru
Biological	GO:00033	amino acid	19/1795	116/2664	0.000269	0.049911	0.048064	arahy.Tifru
Biological	GO:00096	response t	39/1795	322/2664	0.000283	0.049911	0.048064	arahy.Tifru
Biological	GO:00096	phenylpro	31/1795	237/2664	0.000306	0.050023	0.048172	arahy.Tifru
Biological	GO:00485	rhythmic p	38/1795	314/2664	0.000342	0.051668	0.049756	arahy.Tifru
Biological	GO:00161	terpenoid	8/1795	28/26646	0.000384	0.051668	0.049756	arahy.Tifru
Biological	GO:00323	DNA geom	8/1795	28/26646	0.000384	0.051668	0.049756	arahy.Tifru
Biological	GO:00423	indole gluc	7/1795	22/26646	0.000431	0.053605	0.051621	arahy.Tifru
Biological	GO:00300	cellular mc	15/1795	84/26646	0.00045	0.053605	0.051621	arahy.Tifru
Biological	GO:00424	hormone b	46/1795	412/2664	0.000542	0.053605	0.051621	arahy.Tifru
Biological	GO:00069	chemotaxi	10/1795	44/26646	0.000562	0.053605	0.051621	arahy.Tifru
Biological	GO:00101	pollen tub	10/1795	44/26646	0.000562	0.053605	0.051621	arahy.Tifru
Biological	GO:00423	taxis	10/1795	44/26646	0.000562	0.053605	0.051621	arahy.Tifru
Biological	GO:00509	positive ch	10/1795	44/26646	0.000562	0.053605	0.051621	arahy.Tifru
Biological	GO:00310	nuclear mi	5/1795	12/26646	0.000732	0.062107	0.059809	arahy.Tifru
Biological	GO:00995	actin filam	5/1795	12/26646	0.000732	0.062107	0.059809	arahy.Tifru
Biological	GO:00062	DNA replic	9/1795	38/26646	0.000765	0.062107	0.059809	arahy.Tifru
Biological	GO:00096	indoleacet	7/1795	24/26646	0.000777	0.062107	0.059809	arahy.Tifru
Biological	GO:00068	amino acid	25/1795	187/2664	0.000812	0.062107	0.059809	arahy.Tifru
Biological	GO:00550	potassium	11/1795	54/26646	0.000814	0.062107	0.059809	arahy.Tifru
Biological	GO:00158	drug trans	23/1795	167/2664	0.000855	0.063134	0.060798	arahy.Tifru
Biological	GO:00550	cation hor	46/1795	423/2664	0.000947	0.066418	0.063961	arahy.Tifru
Biological	GO:00096	response t	40/1795	354/2664	0.000957	0.066418	0.063961	arahy.Tifru
Biological	GO:00001	sulfate ass	6/1795	19/26646	0.001171	0.078888	0.075969	arahy.Tifru
Biological	GO:00102	response t	23/1795	172/2664	0.001281	0.081154	0.078151	arahy.Tifru
Biological	GO:00424	hormone c	11/1795	57/26646	0.001303	0.081154	0.078151	arahy.Tifru
Biological	GO:00096	indoleacet	7/1795	26/26646	0.001311	0.081154	0.078151	arahy.Tifru
Biological	GO:00347	cellular ho	15/1795	94/26646	0.0015	0.090415	0.08707	arahy.Tifru
Biological	GO:00098	fruit ripen	12/1795	67/26646	0.001579	0.092742	0.08931	arahy.Tifru
Biological	GO:19054	regulation	6/1795	21/26646	0.002085	0.11431	0.11008	arahy.Tifru
Biological	GO:00100	root morpl	45/1795	428/2664	0.00209	0.11431	0.11008	arahy.Tifru
Biological	GO:00069	syncytium	7/1795	28/26646	0.002097	0.11431	0.11008	arahy.Tifru
Biological	GO:00097	indole gluc	5/1795	15/26646	0.00234	0.121784	0.117278	arahy.Tifru
Biological	GO:00307	cytoskelet	5/1795	15/26646	0.00234	0.121784	0.117278	arahy.Tifru
Biological	GO:00485	embryonic	13/1795	80/26646	0.002557	0.129196	0.124416	arahy.Tifru
Biological	GO:00400	locomotio	11/1795	62/26646	0.002636	0.129196	0.124416	arahy.Tifru
Biological	GO:00102	response t	37/1795	339/2664	0.002652	0.129196	0.124416	arahy.Tifru
Biological	GO:00076	circadian r	32/1795	283/2664	0.002885	0.137632	0.132539	arahy.Tifru

Biological p	GO:00709	nuclear mi	5/1795	16/26646	0.003216	0.145239	0.139865	arahy.Tifru
Biological p	GO:00516	nucleus loc	5/1795	16/26646	0.003216	0.145239	0.139865	arahy.Tifru
Biological p	GO:00442	sulfur com	29/1795	251/26646	0.003235	0.145239	0.139865	arahy.Tifru
Biological p	GO:00306	regulation	11/1795	65/26646	0.00386	0.166768	0.160597	arahy.Tifru
Biological p	GO:00514	regulation	11/1795	65/26646	0.00386	0.166768	0.160597	arahy.Tifru
Biological p	GO:00098	lignin bios	17/1795	124/26646	0.004004	0.1698	0.163516	arahy.Tifru
Biological p	GO:00714	cellular res	6/1795	24/26646	0.004347	0.180977	0.17428	arahy.Tifru
Biological p	GO:00300	cellular cat	28/1795	246/26646	0.004635	0.188865	0.181876	arahy.Tifru
Biological p	GO:00987	inorganic i	46/1795	460/26646	0.004876	0.188865	0.181876	arahy.Tifru
Biological p	GO:00424	indole-con	14/1795	96/26646	0.004935	0.188865	0.181876	arahy.Tifru
Biological p	GO:00508	response t	9/1795	49/26646	0.004948	0.188865	0.181876	arahy.Tifru
Biological p	GO:00904	embryonic	9/1795	49/26646	0.004948	0.188865	0.181876	arahy.Tifru
Biological p	GO:19038	organic aci	20/1795	159/26646	0.005186	0.191556	0.184468	arahy.Tifru
Biological p	GO:19050	carboxylic	20/1795	159/26646	0.005186	0.191556	0.184468	arahy.Tifru
Biological p	GO:00068	regulation	15/1795	107/26646	0.005378	0.195468	0.188235	arahy.Tifru
Biological p	GO:00095	fertilizatio	9/1795	50/26646	0.005682	0.203291	0.195769	arahy.Tifru
Biological p	GO:00524	defense re	9/1795	51/26646	0.006496	0.22308	0.214825	arahy.Tifru
Biological p	GO:00525	defense re	9/1795	51/26646	0.006496	0.22308	0.214825	arahy.Tifru
Biological p	GO:00362	cellular res	6/1795	26/26646	0.006624	0.22308	0.214825	arahy.Tifru
Biological p	GO:00714	cellular res	6/1795	26/26646	0.006624	0.22308	0.214825	arahy.Tifru
Biological p	GO:00510	regulation	5/1795	19/26646	0.00723	0.237251	0.228472	arahy.Tifru
Biological p	GO:00098	lignin meta	19/1795	153/26646	0.007252	0.237251	0.228472	arahy.Tifru
Biological p	GO:00313	negative re	20/1795	165/26646	0.007789	0.249215	0.239993	arahy.Tifru
Biological p	GO:00525	defense re	12/1795	81/26646	0.007836	0.249215	0.239993	arahy.Tifru
Biological p	GO:00096	response t	4/1795	13/26646	0.008969	0.270252	0.260252	arahy.Tifru
Biological p	GO:00161	sesquiterp	4/1795	13/26646	0.008969	0.270252	0.260252	arahy.Tifru
Biological p	GO:00432	apocarote	4/1795	13/26646	0.008969	0.270252	0.260252	arahy.Tifru
Biological p	GO:00463	abscisic aci	4/1795	13/26646	0.008969	0.270252	0.260252	arahy.Tifru
Biological p	GO:00098	cytokinin c	5/1795	20/26646	0.009114	0.271062	0.261032	arahy.Tifru
Biological p	GO:19038	regulation	9/1795	55/26646	0.010675	0.313408	0.301811	arahy.Tifru
Biological p	GO:00550	metal ion h	34/1795	334/26646	0.010882	0.315435	0.303763	arahy.Tifru
Biological p	GO:00158	organic aci	32/1795	311/26646	0.011402	0.322351	0.310423	arahy.Tifru
Biological p	GO:00469	carboxylic	32/1795	311/26646	0.011402	0.322351	0.310423	arahy.Tifru
Biological p	GO:00018	selenium c	4/1795	14/26646	0.011896	0.324764	0.312746	arahy.Tifru
Biological p	GO:00300	cellular po	4/1795	14/26646	0.011896	0.324764	0.312746	arahy.Tifru
Biological p	GO:00550	cellular che	31/1795	300/26646	0.011913	0.324764	0.312746	arahy.Tifru
Biological p	GO:00098	jasmonic a	22/1795	195/26646	0.012277	0.328149	0.316007	arahy.Tifru
Biological p	GO:00068	cellular ion	28/1795	265/26646	0.012324	0.328149	0.316007	arahy.Tifru
Biological p	GO:00028	negative re	9/1795	57/26646	0.013399	0.344748	0.331992	arahy.Tifru
Biological p	GO:00321	negative re	9/1795	57/26646	0.013399	0.344748	0.331992	arahy.Tifru
Biological p	GO:00439	negative re	9/1795	57/26646	0.013399	0.344748	0.331992	arahy.Tifru
Biological p	GO:00483	mucilage b	5/1795	22/26646	0.013842	0.348329	0.33544	arahy.Tifru
Biological p	GO:20000	regulation	5/1795	22/26646	0.013842	0.348329	0.33544	arahy.Tifru
Biological p	GO:00096	induced sy	11/1795	78/26646	0.01515	0.36667	0.353103	arahy.Tifru
Biological p	GO:00603	regulation	11/1795	78/26646	0.01515	0.36667	0.353103	arahy.Tifru
Biological p	GO:00550	lipid home	10/1795	68/26646	0.015237	0.36667	0.353103	arahy.Tifru
Biological p	GO:00801	response t	20/1795	176/26646	0.015251	0.36667	0.353103	arahy.Tifru
Biological p	GO:00022	defense re	4/1795	15/26646	0.015371	0.36667	0.353103	arahy.Tifru
Biological p	GO:00716	anatomica	33/1795	331/26646	0.015881	0.374922	0.361049	arahy.Tifru
Biological p	GO:00323	regulation	8/1795	50/26646	0.017798	0.408909	0.393779	arahy.Tifru

Biological p	GO:19026	negative re	39/1795	409/26646	0.018345	0.408909	0.393779	arahy.Tifru
Biological p	GO:19035	negative re	39/1795	409/26646	0.018345	0.408909	0.393779	arahy.Tifru
Biological p	GO:00523	cell wall th	10/1795	70/26646	0.018429	0.408909	0.393779	arahy.Tifru
Biological p	GO:00525	callose dep	10/1795	70/26646	0.018429	0.408909	0.393779	arahy.Tifru
Biological p	GO:00100	primary sh	7/1795	41/26646	0.018571	0.408909	0.393779	arahy.Tifru
Biological p	GO:00102	brassinoste	7/1795	41/26646	0.018571	0.408909	0.393779	arahy.Tifru
Biological p	GO:00090	aromatic a	19/1795	169/26646	0.019601	0.427483	0.411665	arahy.Tifru
Biological p	GO:00488	adventitiou	5/1795	24/26646	0.019986	0.427727	0.4119	arahy.Tifru
Biological p	GO:00703	cellular hea	5/1795	24/26646	0.019986	0.427727	0.4119	arahy.Tifru
Biological p	GO:00100	meristem i	9/1795	61/26646	0.020339	0.431272	0.415313	arahy.Tifru
Biological p	GO:00328	regulation	9/1795	62/26646	0.022418	0.466696	0.449427	arahy.Tifru
Biological p	GO:00423	cuticle dev	9/1795	62/26646	0.022418	0.466696	0.449427	arahy.Tifru
Biological p	GO:19016	glycosyl co	29/1795	291/26646	0.02273	0.468155	0.450832	arahy.Tifru
Biological p	GO:00094	response t	4/1795	17/26646	0.024074	0.468155	0.450832	arahy.Tifru
Biological p	GO:00331	regulation	4/1795	17/26646	0.024074	0.468155	0.450832	arahy.Tifru
Biological p	GO:00331	positive re	4/1795	17/26646	0.024074	0.468155	0.450832	arahy.Tifru
Biological p	GO:00421	negative re	4/1795	17/26646	0.024074	0.468155	0.450832	arahy.Tifru
Biological p	GO:00423	positive re	4/1795	17/26646	0.024074	0.468155	0.450832	arahy.Tifru
Biological p	GO:19001	positive re	4/1795	17/26646	0.024074	0.468155	0.450832	arahy.Tifru
Biological p	GO:00512	negative re	39/1795	417/26646	0.024123	0.468155	0.450832	arahy.Tifru
Biological p	GO:00073	single ferti	6/1795	34/26646	0.024476	0.471014	0.453585	arahy.Tifru
Biological p	GO:00071	cell surface	43/1795	469/26646	0.025095	0.476549	0.458915	arahy.Tifru
Biological p	GO:00217	developme	35/1795	368/26646	0.025217	0.476549	0.458915	arahy.Tifru
Biological p	GO:00424	indole-con	3/1795	10/26646	0.025596	0.476549	0.458915	arahy.Tifru
Biological p	GO:00723	modified a	3/1795	10/26646	0.025596	0.476549	0.458915	arahy.Tifru
Biological p	GO:00096	shade avoi	7/1795	44/26646	0.026599	0.486421	0.468422	arahy.Tifru
Biological p	GO:00102	cellulose m	7/1795	44/26646	0.026599	0.486421	0.468422	arahy.Tifru
Biological p	GO:00467	protein aut	31/1795	320/26646	0.026967	0.486421	0.468422	arahy.Tifru
Biological p	GO:00086	carbohydra	28/1795	283/26646	0.026976	0.486421	0.468422	arahy.Tifru
Biological p	GO:20007	regulation	5/1795	26/26646	0.027682	0.493358	0.475103	arahy.Tifru
Biological p	GO:00713	cellular res	22/1795	211/26646	0.027792	0.493358	0.475103	arahy.Tifru

a15 and Zhonghua12 peanut

Count	up	down
10	0	10
9	0	9
26	0	26
51	32	19
12	5	7
24	12	12
32	21	11
5	1	4
9	7	2
27	12	15
24	17	7
6	3	3
37	30	7
12	5	7
19	8	11
9	0	9
4	3	1
22	14	8
18	8	10
4	1	3
4	3	1
4	3	1
18	8	10
42	24	18
4	1	3
4	0	4
5	1	4
36	15	21
8	8	0
8	3	5
7	6	1
6	4	2
6	4	2
4	2	2
16	8	8
29	19	10
8	6	2
46	29	17
33	21	12
33	21	12
5	3	2
43	24	19
16	11	5
22	8	14
10	3	7
9	2	7
4	0	4
4	1	3

37	20	17
3	2	1
6	4	2
9	6	3
20	12	8
20	12	8
8	3	5
9	3	6
3	1	2
3	1	2
3	1	2
3	1	2
3	2	1
17	7	10
9	2	7
9	2	7
7	3	4
7	3	4
7	3	4
4	1	3
4	3	1
13	6	7
5	3	2
5	3	2
15	5	10
15	5	10
30	9	21
11	8	3
9	3	6
38	24	14
3	0	3
3	0	3
3	0	3
3	0	3
3	0	3
3	2	1
4	3	1
8	3	5
5	0	5
40	23	17
8	5	3
6	1	5
43	24	19
13	9	4
13	9	4
18	9	9
5	3	2
17	6	11
12	10	2
43	24	19

3	2	1
3	3	0
8	0	8
42	19	23
58	32	26
22	15	7
13	2	11
18	12	6
25	12	13
21	15	6
9	4	5
8	0	8
5	0	5
19	14	5
39	20	19
31	14	17
38	10	28
8	4	4
8	0	8
7	4	3
15	9	6
46	25	21
10	2	8
10	2	8
10	2	8
10	2	8
5	1	4
5	1	4
9	0	9
7	5	2
25	18	7
11	4	7
23	15	8
46	24	22
40	7	33
6	2	4
23	12	11
11	6	5
7	5	2
15	9	6
12	5	7
6	4	2
45	22	23
7	6	1
5	4	1
5	1	4
13	8	5
11	3	8
37	14	23
32	8	24

5	1	4
5	1	4
29	11	18
11	7	4
11	7	4
17	8	9
6	6	0
28	14	14
46	24	22
14	10	4
9	4	5
9	5	4
20	15	5
20	15	5
15	11	4
9	2	7
9	6	3
9	6	3
6	6	0
6	6	0
5	0	5
19	8	11
20	8	12
12	7	5
4	1	3
4	1	3
4	1	3
4	1	3
5	3	2
9	5	4
34	16	18
32	20	12
32	20	12
4	1	3
4	2	2
31	14	17
22	7	15
28	14	14
9	4	5
9	4	5
9	4	5
5	4	1
5	0	5
11	5	6
11	5	6
10	7	3
20	7	13
4	2	2
33	17	16
8	5	3

39	15	24
39	15	24
10	7	3
10	7	3
7	4	3
7	5	2
19	10	9
5	4	1
5	3	2
9	5	4
9	5	4
9	7	2
29	17	12
4	1	3
4	4	0
4	4	0
4	1	3
4	4	0
4	4	0
39	15	24
6	2	4
43	26	17
35	18	17
3	0	3
3	2	1
7	5	2
7	3	4
31	12	19
28	13	15
5	4	1
22	7	15
