# Supplementary material for: Transcriptome and Metabolome Analysis Unveil Anthocyanin Metabolism in Pink and Red Testa of Peanut (Arachis hypogaea L.)
Source: Int J Genomics. 2021 Aug 6;2021:5883901. doi: 10.1155/2021/5883901 (PMC8363441; doi:10.1155/2021/5883901)
Supplement: Supplementary Materials — Supplementary Figure 1: GO classification of differentially expressed genes of R7 stage of Shanhua15 and Zhonghua12 testa. Supplementary Figure 2: COG functional classification of differentially expressed genes of R4 and R7 stages of Shanhua15 and Zhonghua12 testa. S4, Shanhua15 R4 pod period; Z4, Zhonghua12 R4 pod period; S7, Shanhua15 R7 pod period; Z7, Zhonghua12 R7 pod period. Supplementary Table 1: primers for qRT-PCR verification. Supplementary Table 2: significantly changed metabolites between Shanhua15 and Zhonghua12 testa. Supplementary Table 3: summary of the sequencing and de novo assembly. Supplementary Table 4: statistics of GO Enrichment in the testa of R4 and R7 stages of Shanhua15 and Zhonghua12 peanut. Supplementary Table 5: DEGs of flavonoid and anthocyanin biosynthesis in the testa of R4 and R7 stages of Shanhua15 and Zhonghua12 peanut. Supplementary Table 6: differentially expressed transcription factors in the testa of R4 and R7 stages of Shanhua15 and Zhonghua12 peanut. [file 5883901.f1.zip › 5883901.f1/Table S5.pdf]

Table S4 DEGs of flavonoid and anthocyanin biosynthesis in the testa of

Gene name	ID	Shanhua15R	Shanhua15R	log2FoldChange
PAL	arahy.Tifrunner.gnm1.ann1.NB2RRK	413	147	-1.492
	arahy.Tifrunner.gnm1.ann1.PUMP6L	4810	1025	-2.231
C4H	arahy.Tifrunner.gnm1.ann1.AYA1A5	62	4	-4.14
	arahy.Tifrunner.gnm1.ann1.K1LFJJ	53	5	-3.359
	arahy.Tifrunner.gnm1.ann1.SGZ2CH	7874	3613	-1.124
4CL	arahy.Tifrunner.gnm1.ann1.35X8AA	530	73	-2.853
	arahy.Tifrunner.gnm1.ann1.7DZV4F	17	0	-6.641
	arahy.Tifrunner.gnm1.ann1.BDR360	137	40	-1.771
	arahy.Tifrunner.gnm1.ann1.IT8P61	742	340	-1.127
	arahy.Tifrunner.gnm1.ann1.L700WL	79	282	1.841
	arahy.Tifrunner.gnm1.ann1.W43CKL	643	1785	1.474
CHS	arahy.Tifrunner.gnm1.ann1.6J3HHE	110	38	-1.549
	arahy.Tifrunner.gnm1.ann1.ALW2B1	22	1095	5.634
	arahy.Tifrunner.gnm1.ann1.JH8TMD	67	15	-2.198
	arahy.Tifrunner.gnm1.ann1.K0UY8K	117	19	-2.618
	arahy.Tifrunner.gnm1.ann1.QZN0LQ	111	10	-3.551
	arahy.Tifrunner.gnm1.ann1.UDJX6I	3893	1897	-1.037
F3H	arahy.Tifrunner.gnm1.ann1.79B99S	2518	525	-2.261
	arahy.Tifrunner.gnm1.ann1.HE8J5U	2687	685	-1.973
F3'H	arahy.Tifrunner.gnm1.ann1.8F7PE4	3121	876	-1.834
	arahy.Tifrunner.gnm1.ann1.K8H9R8	1769	453	-1.966
DFR	arahy.Tifrunner.gnm1.ann1.B0KX4P	68	20	-1.786
ANS	arahy.Tifrunner.gnm1.ann1.AQ6B1J	909	128	-2.826
	arahy.Tifrunner.gnm1.ann1.UQ0Z3E	799	138	-2.53
ANR	arahy.Tifrunner.gnm1.ann1.IK60LM	753	106	-2.824
	arahy.Tifrunner.gnm1.ann1.W8TDEC	100	36	-1.48
LAR	arahy.Tifrunner.gnm1.ann1.T1J2UZ	643	6	-6.76
	arahy.Tifrunner.gnm1.ann1.M05HM0	580	7	-6.381
FLS	arahy.Tifrunner.gnm1.ann1.4WXU8P	1840	113	-4.018
	arahy.Tifrunner.gnm1.ann1.4Y1607	2828	174	-4.018

R4 and R7 stages of Shanhua15 and Zhonghua12 peanut

pvalue	FDR	regulated	Name	Chrom	Start	End	Strand	Length
0.00151	0.018568	down	ahy.NB2RI	unner.gnm1	1.46E+08	1.46E+08	-	7414
7.2E-06	0.000252	down	ahy.PUMP	unner.gnm1	42030770	42036927	+	6158
5.38E-08	3.99E-06	down	ahy.AYA1	unner.gnm1	2642047	2650511	+	8465
8.01E-05	0.001857	down	rahy.K1LF	unner.gnm1	8101429	8107794	-	6366
0.002131	0.024098	down	ahy.SGZ2C	unner.gnm1	21279765	21284834	+	5070
6.61E-06	0.000235	down	ahy.35X8A	unner.gnm1	1.48E+08	1.48E+08	-	6568
0.000202	0.003955	down	ahy.7DZV	unner.gnm1	8358130	8363858	-	5729
7.47E-08	5.25E-06	down	ahy.BDR3	unner.gnm1	29365019	29368436	-	3418
0.00022	0.004231	down	rahy.IT8P6	unner.gnm1	96248067	96251891	+	3825
1.28E-08	1.16E-06	up	ahy.L700W	unner.gnm1	50725449	50731693	+	6245
0.001335	0.016966	up	ahy.W43CI	unner.gnm1	5092509	5096167	-	3659
0.000204	0.00399	down	ahy.6J3HF	unner.gnm1	18572663	18574554	-	1892
3.79E-34	2.18E-30	up	ahy.ALW2	unner.gnm1	1.29E+08	1.29E+08	+	4179
0.001883	0.021999	down	ahy.JH8TM	unner.gnm1	38986848	38988503	+	1656
1.09E-09	1.29E-07	down	ahy.K0UY	unner.gnm1	38961309	38963310	-	2002
5.45E-13	1.5E-10	down	ahy.QZN0I	unner.gnm1	18595150	18597004	+	1855
0.000722	0.010674	down	ahy.UDJX	unner.gnm1	8632413	8635267	-	2855
2.11E-12	4.88E-10	down	rahy.79B9	unner.gnm1	11007083	11012015	-	4933
4.86E-11	8.2E-09	down	ahy.HE8J5	unner.gnm1	673566	679235	+	5670
1.39E-11	2.6E-09	down	rahy.8F7PE	unner.gnm1	1.3E+08	1.3E+08	-	5438
5.81E-12	1.22E-09	down	ahy.K8H9I	unner.gnm1	1.06E+08	1.06E+08	-	5230
0.004007	0.038214	down	ahy.B0KX	unner.gnm1	44757364	44760433	-	3070
2.19E-27	6.79E-24	down	ahy.AQ6B	unner.gnm1	12997611	12999986	+	2376
4.91E-15	2.13E-12	down	ahy.UQ0Z	unner.gnm1	13644667	13647176	+	2510
2.65E-10	3.71E-08	down	ahy.IK60L	unner.gnm1	1.17E+08	1.17E+08	-	2631
5.3E-05	0.00132	down	ahy.W8TD	unner.gnm1	1.26E+08	1.26E+08	+	2680
5.81E-16	3.21E-13	down	rahy.T1J2U	unner.gnm1	1.1E+08	1.1E+08	+	6039
6.42E-46	1.29E-41	down	ahy.M05HM	unner.gnm1	1.19E+08	1.19E+08	-	6084
6.09E-12	1.26E-09	down	ahy.4WXU	unner.gnm1	19357631	19364660	-	7030
2.57E-19	2.59E-16	down	rahy.4Y16C	unner.gnm1	24219840	24228275	-	8436

Type	GO	KEGG	KOG	NR	SwissProt	TrEMBL
otein_codi	0009800,c	anine amm	At3g53260	onia-lyase	henylalanin	000256|RuleBase:RU003955}; E
otein_codi	0009800,c	anine amm	At2g37040	onia-lyase	henylalanin	000256|RuleBase:RU003955}; E
otein_codi	e 4-monoo	amate 4-mo	At2g30490	nooxygena	acid 4-hydr	racterized protein {ECO:000031
otein_codi	e 4-monoo	amate 4-mo	At2g30490	ooxygena	acid 4-hydr	racterized protein {ECO:000031
otein_codi	e 4-monoo	amate 4-mo	At2g30490	nooxygena	acid 4-hydr	0313|EMBL:KRH17200.1, ECO:
otein_codi	524,ATP b	narate--Co	At3g21240	e 2 isofor	hort=4CL	0313|EMBL:KRH02884.1, ECO:
otein_codi	########	narate--Co	At1g62940	igase-like	C=6.2.1.-; A	--CoA ligase-like 1 {ECO:00003
otein_codi	0016874,li	narate--Co	At4g05160	gase-like 7	C=6.2.1.-;	--CoA ligase-like 7 {ECO:00003
otein_codi	524,ATP b	narate--Co	At3g21240	gase 2-like	hort=4CL	0313|EMBL:KRH02884.1, ECO:
otein_codi	0016874,li	--	At5g63380	gase-like 9	C=6.2.1.-;	oA ligase-like protein {ECO:000
otein_codi	0016874,li	narate--Co	At4g05160	gase-like 7	C=6.2.1.-;	--CoA ligase-like 7 {ECO:00003
otein_codi	e activity; C	lcone synt	At5g13930	se 6 [Arac	; EC=2.3.1	one synthase {ECO:0000313|EM
otein_codi	e activity; C	lcone synt	At5g13930	hase [Arac	; EC=2.3.1	halcone synthase {ECO:0000313
otein_codi	e activity; C	lcone synt	At5g13930	se 6 [Arac	; EC=2.3.1	one synthase {ECO:0000313|EM
otein_codi	e activity; C	lcone synt	At5g13930	se 6 [Arac	; EC=2.3.1	one synthase {ECO:0000313|EM
otein_codi	e activity; C	lcone synt	At5g13930	se 6 [Arac	; EC=2.3.1	one synthase {ECO:0000313|EM
otein_codi	e activity; C	lcone synt	At5g13930	ase [Arach	; EC=2.3.1	one synthase {ECO:0000313|EM
otein_codi	045486,na	genin 3-di	At3g51240	3-dioxyge	UniProtKB	none-3-hydroxylase {ECO:0000
otein_codi	045486,na	genin 3-di	At3g51240	e 3-dioxyg	UniProtKB	none-3-hydroxylase {ECO:0000
otein_codi	3'-monoox	oid 3'-mon	At5g07990	oxygenase	21; AltNam	=F3'H {ECO:0000313|EMBL:AE
otein_codi	3'-monoox	oid 3'-mon	At5g07990	xygenase [	21; AltNam	=F3'H {ECO:0000313|EMBL:AE
otein_codi	ty; GO:000	tone reduct	At2g45400	ctase [Ara	Full=Vestit	flavonol-4-reductase {ECO:0000
otein_codi	O:0046872	cyanidin di	At4g22880	dioxygena	4.11.19 {E	ocyanidin synthase {ECO:00003
otein_codi	O:0046872	cyanidin di	At4g22880	dioxygena	4.11.19 {E	ocyanidin synthase {ECO:00003
otein_codi	reductase	cyanidin re	At1g61720	-flavan-3-o	KB:Q5FB34	ocyanidin reductase {ECO:00003
otein_codi	reductase	cyanidin re	At1g61720	-flavan-3-o	KB:Q5FB34	ocyanidin reductase {ECO:00003
otein_codi	ivity; GO:0	hocyanidin	At1g75290	reductase |	lin reducta	idin reductase LAR1-2 {ECO:00
otein_codi	ivity; GO:0	hocyanidin	At1g75290	eductase-li	lin reducta	idin reductase LAR1-2 {ECO:00
otein_codi	bic acid bi	onol synth	At5g08640	e 3-hydrox	.14.11.23	nol synthase {ECO:0000313|EMI
otein_codi	bic acid bi	onol synth	At5g08640	ne 3-hydro	.14.11.23	onol synthase {ECO:0000313|EM

C=4.3.1.24 {ECO:0000256|RuleBase:RU003955};
C=4.3.1.24 {ECO:0000256|RuleBase:RU003955};
3|EMBL:KDP45711.1};
3|EMBL:KDP45711.1};
:0000313|EnsemblPlants:GLYMA14G38580.1};
:0000313|EnsemblPlants:GLYMA17G07170.1};
13|RefSeq:XP_004516098.1};
13|RefSeq:XP_014498368.1};
:0000313|EnsemblPlants:GLYMA17G07170.1};
0313|EMBL:AES72328.1};
13|RefSeq:XP_014498368.1};
IBL:AAO32821.1};
|EMBL:AID59203.1};
IBL:AAO32821.1};
IBL:AAO32821.1};
IBL:AAO32821.1};
IBL:ACB78187.1};
313|EMBL:AID59205.1};
313|EMBL:AID59205.1};
X07282.1};
X07282.1};
313|EMBL:KYP52299.1};
13|EMBL:AID59207.1};
13|EMBL:AID59207.1};
13|EMBL:AJF94633.1};
13|EMBL:AJF94633.1};
00313|EMBL:ABC71325.1};
00313|EMBL:ABC71325.1};
3L:BAF49296.1};
IBL:BAF31231.1};
