# Supplementary material for: Transcriptome and Metabolome Analysis Unveil Anthocyanin Metabolism in Pink and Red Testa of Peanut (Arachis hypogaea L.)
Source: Int J Genomics. 2021 Aug 6;2021:5883901. doi: 10.1155/2021/5883901 (PMC8363441; doi:10.1155/2021/5883901)
Supplement: Supplementary Materials — Supplementary Figure 1: GO classification of differentially expressed genes of R7 stage of Shanhua15 and Zhonghua12 testa. Supplementary Figure 2: COG functional classification of differentially expressed genes of R4 and R7 stages of Shanhua15 and Zhonghua12 testa. S4, Shanhua15 R4 pod period; Z4, Zhonghua12 R4 pod period; S7, Shanhua15 R7 pod period; Z7, Zhonghua12 R7 pod period. Supplementary Table 1: primers for qRT-PCR verification. Supplementary Table 2: significantly changed metabolites between Shanhua15 and Zhonghua12 testa. Supplementary Table 3: summary of the sequencing and de novo assembly. Supplementary Table 4: statistics of GO Enrichment in the testa of R4 and R7 stages of Shanhua15 and Zhonghua12 peanut. Supplementary Table 5: DEGs of flavonoid and anthocyanin biosynthesis in the testa of R4 and R7 stages of Shanhua15 and Zhonghua12 peanut. Supplementary Table 6: differentially expressed transcription factors in the testa of R4 and R7 stages of Shanhua15 and Zhonghua12 peanut. [file 5883901.f1.zip › 5883901.f1/Table S6.pdf]

Table S5 Differentially expressed transcript

ID	Family	Category	lassificatio	Name	Chrom	hanhua15R	ionghua12	log2FoldChan
ner.gnm1.a	Alfin-like	TF	Alfin-like	ahy.TFSP1	inner.gnm1	1093	533	-1.037
ner.gnm1.a	P2/ERF-AI	TF	F->AP2/E	ahy.K6QU	inner.gnm1	92	24	-1.911
ner.gnm1.a	P2/ERF-AI	TF	F->AP2/E	ahy.QEK7	inner.gnm1	167	11	-3.858
ner.gnm1.a	P2/ERF-AI	TF	F->AP2/E	ahy.UU942	inner.gnm1	41	89	1.141
ner.gnm1.a	P2/ERF-EI	TF	F->AP2/E	ahy.15RN7	inner.gnm1	7	44	2.634
ner.gnm1.a	P2/ERF-EI	TF	F->AP2/E	ahy.45NR7	inner.gnm1	4196	1266	-1.728
ner.gnm1.a	P2/ERF-EI	TF	F->AP2/E	ahy.47NS3	inner.gnm1	352	747	1.088
ner.gnm1.a	P2/ERF-EI	TF	F->AP2/E	ahy.4G06N	inner.gnm1	934	1892	1.02
ner.gnm1.a	P2/ERF-EI	TF	F->AP2/E	ahy.5KI8F	inner.gnm1	743	332	-1.165
ner.gnm1.a	P2/ERF-EI	TF	F->AP2/E	ahy.73DHZ	inner.gnm1	442	148	-1.578
ner.gnm1.a	P2/ERF-EI	TF	F->AP2/E	ahy.87AL6	inner.gnm1	457	48	-3.247
ner.gnm1.a	P2/ERF-EI	TF	F->AP2/E	ahy.9V08H	inner.gnm1	48	5	-3.279
ner.gnm1.a	P2/ERF-EI	TF	F->AP2/E	ahy.E9BL7	inner.gnm1	0	6	5.262
ner.gnm1.a	P2/ERF-EI	TF	F->AP2/E	ahy.ER44I	inner.gnm1	6651	2732	-1.283
ner.gnm1.a	P2/ERF-EI	TF	F->AP2/E	ahy.HGAZ	inner.gnm1	2596	6641	1.355
ner.gnm1.a	P2/ERF-EI	TF	F->AP2/E	ahy.JE3RI	inner.gnm1	2	21	3.526
ner.gnm1.a	P2/ERF-EI	TF	F->AP2/E	ahy.KF0N	inner.gnm1	543	94	-2.528
er.gnm1.a	P2/ERF-EI	TF	F->AP2/E	ahy.L5HMI	inner.gnm1	714	324	-1.142
ner.gnm1.a	P2/ERF-EI	TF	F->AP2/E	ahy.L5Q3A	inner.gnm1	2	30	4.064
ner.gnm1.a	P2/ERF-EI	TF	F->AP2/E	ahy.L65DI	inner.gnm1	3	29	3.499
ner.gnm1.a	P2/ERF-EI	TF	F->AP2/E	rahy.LF78J	inner.gnm1	20	0	-6.649
er.gnm1.a	P2/ERF-EI	TF	F->AP2/E	ahy.M71N	inner.gnm1	89	4	-4.353
ner.gnm1.a	P2/ERF-EI	TF	F->AP2/E	ahy.P2THI	inner.gnm1	6	62	3.534
ner.gnm1.a	P2/ERF-EI	TF	F->AP2/E	ahy.RN6K	inner.gnm1	71	27	-1.418
ner.gnm1.a	P2/ERF-EI	TF	F->AP2/E	ahy.ULX5	inner.gnm1	255	27	-3.248
ner.gnm1.a	P2/ERF-EI	TF	F->AP2/E	ahy.VJ8B3	inner.gnm1	183	386	1.077
ner.gnm1.a	P2/ERF-EI	TF	F->AP2/E	ahy.XJX19	inner.gnm1	0	11	6.029
er.gnm1.a	P2/ERF-RA	TF	F->AP2/EI	ahy.PDF2M	inner.gnm1	770	77	-3.327
ner.gnm1.a	ARID	TR	ARID	ahy.J9QP8	inner.gnm1	84	8	-3.415
ner.gnm1.a	ARID	TR	ARID	ahy.K5DII	inner.gnm1	49	13	-1.929
ner.gnm1.a	AUX/IAA	TR	AUX/IAA	rahy.045PL	inner.gnm1	81	24	-1.73
ner.gnm1.a	AUX/IAA	TR	AUX/IAA	ahy.32M24	inner.gnm1	642	115	-2.479
ner.gnm1.a	AUX/IAA	TR	AUX/IAA	ahy.5P5C9	inner.gnm1	348	1455	2.063
ner.gnm1.a	AUX/IAA	TR	AUX/IAA	rahy.5PTI6	inner.gnm1	75	11	-2.727
ner.gnm1.a	AUX/IAA	TR	AUX/IAA	ahy.85LRI	inner.gnm1	158	76	-1.058
ner.gnm1.a	AUX/IAA	TR	AUX/IAA	ahy.944YY	inner.gnm1	139	298	1.105
ner.gnm1.a	AUX/IAA	TR	AUX/IAA	ahy.F330X	inner.gnm1	550	167	-1.721
ner.gnm1.a	AUX/IAA	TR	AUX/IAA	ahy.F753K	inner.gnm1	43	3	-3.903
ner.gnm1.a	AUX/IAA	TR	AUX/IAA	ahy.N2VZ4	inner.gnm1	117	7	-4.005
ner.gnm1.a	AUX/IAA	TR	AUX/IAA	ahy.P8YR	inner.gnm1	6240	2044	-1.61
ner.gnm1.a	AUX/IAA	TR	AUX/IAA	ahy.P8YR	inner.gnm1	6240	2044	-1.61
er.gnm1.a	AUX/IAA	TR	AUX/IAA	ahy.QUY0	inner.gnm1	79	11	-2.798
er.gnm1.a	AUX/IAA	TR	AUX/IAA	ahy.VW0T	inner.gnm1	213	77	-1.475

ıer.gnm1.aı	B3	TF	B3->B3	ahy.00BQA	ınner.gnm1	7	34	2.366
ıer.gnm1.aı	B3	TF	B3->B3	ahy.28CQX	ınner.gnm1	38	178	2.216
ıer.gnm1.a	B3	TF	B3->B3	ahy.4VG87	ınner.gnm1	104	8	-3.792
ner.gnm1.a	B3	TF	B3->B3	ahy.6FQ15	ınner.gnm1	41	5	-3.025
ıner.gnm1.a	B3	TF	B3->B3	rahy.6PZ11	ınner.gnm1	94	257	1.454
ner.gnm1.a	B3	TF	B3->B3	ahy.7Y7E6	ınner.gnm1	366	953	1.38
ıer.gnm1.aı	B3	TF	B3->B3	ahy.DRN5	ınner.gnm1	23	0	-6.859
ıer.gnm1.aı	B3	TF	B3->B3	ahy.ES60G	ınner.gnm1	13	1	-3.177
ıer.gnm1.aı	B3	TF	B3->B3	ahy.HB4W	ınner.gnm1	81	175	1.103
ner.gnm1.a	B3	TF	B3->B3	ahy.I35XG	ınner.gnm1	519	1076	1.052
ıner.gnm1.a	B3	TF	B3->B3	rahy.IR6J7	ınner.gnm1	74	9	-3.02
ıer.gnm1.aı	B3	TF	B3->B3	ahy.WFEC	ınner.gnm1	119	42	-1.516
er.gnm1.aı	B3	TF	B3->B3	ahy.YY0B0	ınner.gnm1	167	483	1.537
ner.gnm1.a	B3	TF	B3->B3	rahy.ZJXJ5	ınner.gnm1	212	649	1.616
ner.gnm1.a	B3-ARF	TF	3->B3-AR	ahy.22510	ınner.gnm1	327	960	1.553
ıer.gnm1.a	B3-ARF	TF	3->B3-AR	ahy.2YRL0	ınner.gnm1	105	34	-1.628
ıner.gnm1.a	B3-ARF	TF	3->B3-AR	rahy.53DI9	ınner.gnm1	570	171	-1.736
ıer.gnm1.aı	B3-ARF	TF	3->B3-AR	ahy.A1HL2	ınner.gnm1	191	50	-1.929
ıer.gnm1.a	B3-ARF	TF	3->B3-AR	ahy.BC5R4	ınner.gnm1	88	43	-1.051
ıer.gnm1.aı	B3-ARF	TF	3->B3-AR	ahy.D4KBI	ınner.gnm1	350	68	-2.358
ıer.gnm1.a	B3-ARF	TF	3->B3-AR	ahy.I1MH9	ınner.gnm1	59	12	-2.24
er.gnm1.aı	B3-ARF	TF	3->B3-AR	hy.WDRD	ınner.gnm1	1505	3142	1.062
er.gnm1.aı	B3-ARF	TF	3->B3-AR	hy.WQVL	ınner.gnm1	319	950	1.575
er.gnm1.an	B3-ARF	TF	3->B3-AR	hy.X1U4M	ınner.gnm1	770	308	-1.324
ıer.gnm1.a	B3-ARF	TF	3->B3-AR	ahy.ZE2N9	ner.gnm1.	5	25	2.378
ner.gnm1.a	bHLH	TF	bHLH	ahy.00JV9	ınner.gnm1	826	18	-5.485
ner.gnm1.a	bHLH	TF	bHLH	ahy.02IZM	ınner.gnm1	16	0	-6.335
ıer.gnm1.a	bHLH	TF	bHLH	ahy.0ZB9Y	ınner.gnm1	92	13	-2.83
ıer.gnm1.a	bHLH	TF	bHLH	ahy.160YL	ınner.gnm1	690	260	-1.407
ıer.gnm1.a	bHLH	TF	bHLH	ahy.1C4B0	ınner.gnm1	177	11	-4.002
ıner.gnm1.a	bHLH	TF	bHLH	rahy.2031J	ınner.gnm1	416	17	-4.62
ner.gnm1.a	bHLH	TF	bHLH	ahy.24ZZ8	ınner.gnm1	13	0	-6.026
ner.gnm1.a	bHLH	TF	bHLH	ahy.26781	ınner.gnm1	1693	356	-2.251
ıer.gnm1.a	bHLH	TF	bHLH	ahy.2Y1E4	ınner.gnm1	2	151	6.379
ner.gnm1.a	bHLH	TF	bHLH	ahy.3CP6K	ınner.gnm1	46	4	-3.401
ıner.gnm1.a	bHLH	TF	bHLH	rahy.48I8D	ınner.gnm1	2	18	3.349
ner.gnm1.a	bHLH	TF	bHLH	ahy.4TH67	ınner.gnm1	12	0	-5.899
ıer.gnm1.aı	bHLH	TF	bHLH	ahy.4XX97	ınner.gnm1	31	5	-2.752
ıer.gnm1.a	bHLH	TF	bHLH	ahy.5BA4E	ınner.gnm1	334	19	-4.184
ner.gnm1.a	bHLH	TF	bHLH	ahy.714UE	ınner.gnm1	36	6	-2.489
ner.gnm1.a	bHLH	TF	bHLH	ahy.7G8F9	ınner.gnm1	118	39	-1.588
ner.gnm1.a	bHLH	TF	bHLH	ahy.96VL2	ınner.gnm1	905	303	-1.58
ıer.gnm1.aı	bHLH	TF	bHLH	ahy.AUJU4	ınner.gnm1	1782	144	-3.632
ıer.gnm1.a	bHLH	TF	bHLH	ahy.CWTI0	ınner.gnm1	0	7	5.388
ıer.gnm1.aı	bHLH	TF	bHLH	ahy.E5BLP	ınner.gnm1	1	97	6.333

ner.gnm1.a	bHLH	TF	bHLH	ahy.EG7Binner.gnm1	2701	1079	-1.324
ner.gnm1.a	bHLH	TF	bHLH	ahy.FH0TLinner.gnm1	182	1	-7.957
ner.gnm1.a	bHLH	TF	bHLH	ahy.G6BHlinner.gnm1	19	2	-3.64
ner.gnm1.an	bHLH	TF	bHLH	ahy.GL41Vinner.gnm1	1872	751	-1.318
ner.gnm1.a	bHLH	TF	bHLH	ahy.H39FEinner.gnm1	119	32	-1.876
ner.gnm1.a	bHLH	TF	bHLH	ahy.HM1IVinner.gnm1	497	49	-3.349
ner.gnm1.a	bHLH	TF	bHLH	ahy.HR6FNinner.gnm1	106	0	-9.037
ner.gnm1.a	bHLH	TF	bHLH	ahy.HVT1(inner.gnm1	11	318	4.833
ner.gnm1.a	bHLH	TF	bHLH	ahy.I9XMinner.gnm1	380	3	-6.938
ner.gnm1.a	bHLH	TF	bHLH	rahy.J0D1Jinner.gnm1	662	253	-1.389
ner.gnm1.a	bHLH	TF	bHLH	ahy.JV9T2inner.gnm1	22	1	-4.872
ner.gnm1.a	bHLH	TF	bHLH	ahy.K3IFFinner.gnm1	11	177	4.023
ner.gnm1.a	bHLH	TF	bHLH	ahy.K68B(inner.gnm1	414	134	-1.628
er.gnm1.an	bHLH	TF	bHLH	hy.L9MGVinner.gnm1	60	24	-1.332
ner.gnm1.a	bHLH	TF	bHLH	ahy.MB81inner.gnm1	131	45	-1.547
er.gnm1.an	bHLH	TF	bHLH	hy.MW3S(inner.gnm1	97	19	-2.372
ner.gnm1.a	bHLH	TF	bHLH	ahy.MX4L(inner.gnm1	142	8	-4.198
er.gnm1.an	bHLH	TF	bHLH	hy.MXM7(inner.gnm1	268	34	-2.976
ner.gnm1.a	bHLH	TF	bHLH	ahy.N7IIZinner.gnm1	31	445	3.854
er.gnm1.an	bHLH	TF	bHLH	hy.NX4W(inner.gnm1	312	798	1.353
ner.gnm1.a	bHLH	TF	bHLH	ahy.P97QTinner.gnm1	40	189	2.237
ner.gnm1.a	bHLH	TF	bHLH	ahy.PC0Ginner.gnm1	156	635	2.03
ner.gnm1.a	bHLH	TF	bHLH	ahy.PYSXinner.gnm1	1	15	4.582
ner.gnm1.a	bHLH	TF	bHLH	ahy.QSZJ9inner.gnm1	8	0	-5.267
ner.gnm1.a	bHLH	TF	bHLH	ahy.RSI8Tinner.gnm1	340	44	-2.963
ner.gnm1.a	bHLH	TF	bHLH	ahy.RSVCinner.gnm1	30	3	-3.361
ner.gnm1.a	bHLH	TF	bHLH	ahy.SH99Dinner.gnm1	21	81	1.938
ner.gnm1.a	bHLH	TF	bHLH	ahy.SQH7inner.gnm1	74	11	-2.751
ner.gnm1.a	bHLH	TF	bHLH	ahy.SVE7(inner.gnm1	7868	1464	-2.426
ner.gnm1.a	bHLH	TF	bHLH	ahy.T023Hinner.gnm1	10	265	4.72
ner.gnm1.a	bHLH	TF	bHLH	ahy.TWQ0inner.gnm1	230	51	-2.176
ner.gnm1.a	bHLH	TF	bHLH	ahy.U584Ninner.gnm1	19	1	-4.698
ner.gnm1.a	bHLH	TF	bHLH	ahy.VFS8Minner.gnm1	146	16	-3.202
ner.gnm1.a	bHLH	TF	bHLH	ahy.W9Z2inner.gnm1	221	42	-2.402
er.gnm1.an	bHLH	TF	bHLH	hy.X3LKCinner.gnm1	57	20	-1.503
ner.gnm1.a	bHLH	TF	bHLH	ahy.XN5C4inner.gnm1	8971	1335	-2.748
ner.gnm1.a	bHLH	TF	bHLH	ahy.Y3C6Linner.gnm1	1	12	3.734
ner.gnm1.a	bHLH	TF	bHLH	ahy.Z8D0Ninner.gnm1	10	0	-5.63
ner.gnm1.a	bHLH	TF	bHLH	ahy.ZXK4Iinner.gnm1	0	15	5.55
ner.gnm1.a	bZIP	TF	bZIP	ahy.0BLG9inner.gnm1	148	1	-7.647
ner.gnm1.a	bZIP	TF	bZIP	ahy.1A1A(inner.gnm1	199	94	-1.085
ner.gnm1.a	bZIP	TF	bZIP	ahy.1B2Z8inner.gnm1	779	2402	1.624
ner.gnm1.a	bZIP	TF	bZIP	ahy.1D6R9inner.gnm1	31	4	-2.863
ner.gnm1.a	bZIP	TF	bZIP	ahy.53DGinner.gnm1	18	270	3.953
ner.gnm1.a	bZIP	TF	bZIP	ahy.661M(inner.gnm1	8	48	2.615

ner.gnm1.a	bZIP	TF	bZIP	ahy.6K442	nner.gnm1	554	1843	1.735
ner.gnm1.ar	bZIP	TF	bZIP	ahy.71DGV	nner.gnm1	94	1	-6.019
ner.gnm1.ar	bZIP	TF	bZIP	ahy.8U3XL	nner.gnm1	23	0	-6.833
ner.gnm1.a	bZIP	TF	bZIP	ahy.980X4	nner.gnm1	140	385	1.458
ner.gnm1.ar	bZIP	TF	bZIP	ahy.9KRV5	nner.gnm1	132	330	1.323
ner.gnm1.a	bZIP	TF	bZIP	ahy.9PR62	nner.gnm1	1227	240	-2.354
ner.gnm1.a	bZIP	TF	bZIP	ahy.A7US1	nner.gnm1	28	76	1.458
ner.gnm1.a	bZIP	TF	bZIP	rahy.AAI19	nner.gnm1	20	75	1.884
ner.gnm1.ar	bZIP	TF	bZIP	ahy.AFV1I	nner.gnm1	182	550	1.597
ner.gnm1.ar	bZIP	TF	bZIP	ahy.B9A4X	nner.gnm1	24	62	1.362
ner.gnm1.a	bZIP	TF	bZIP	ahy.F95VP	nner.gnm1	173	5	-5.311
ner.gnm1.ar	bZIP	TF	bZIP	ahy.H6GQI	nner.gnm1	4	30	3.03
ner.gnm1.a	bZIP	TF	bZIP	ahy.K23Z1	nner.gnm1	965	1948	1.013
er.gnm1.an	bZIP	TF	bZIP	hy.KZ9VV	nner.gnm1	695	1409	1.019
er.gnm1.ar	bZIP	TF	bZIP	hy.MHQ5	nner.gnm1	111	53	-1.07
ner.gnm1.a	bZIP	TF	bZIP	ahy.R46QI	nner.gnm1	36	7	-2.431
ner.gnm1.a	bZIP	TF	bZIP	ahy.RIV6Z	nner.gnm1	101	729	2.855
ner.gnm1.ar	bZIP	TF	bZIP	ahy.T95EU	nner.gnm1	2274	6150	1.435
ner.gnm1.a	bZIP	TF	bZIP	ahy.V67U6	nner.gnm1	46	0	-7.845
ner.gnm1.a	bZIP	TF	bZIP	rahy.VA7II	nner.gnm1	1564	4851	1.634
ner.gnm1.a	bZIP	TF	bZIP	ahy.W16IV	nner.gnm1	2074	428	-2.278
ner.gnm1.a	bZIP	TF	bZIP	ahy.ZES6C	nner.gnm1	415	51	-3.021
ner.gnm1.a	2C2-CO-lil	TF	->C2C2-C	ahy.8MV2	nner.gnm1	333	80	-2.054
ner.gnm1.a	2C2-CO-lil	TF	->C2C2-C	ahy.AY8I6	nner.gnm1	363	39	-3.216
er.gnm1.ar	2C2-CO-lil	TF	->C2C2-C	hy.B9QMl	nner.gnm1	634	187	-1.763
er.gnm1.ar	2C2-CO-lil	TF	->C2C2-C	hy.WVH2	nner.gnm1	163	12	-3.797
er.gnm1.ar	2C2-CO-lil	TF	->C2C2-C	hy.ZS9VW	nner.gnm1	542	209	-1.375
ner.gnm1.a	2C2-CO-lil	TF	->C2C2-C	ahy.ZUL46	nner.gnm1	224	1	-7.26
ner.gnm1.ar	C2C2-Dof	TF	2->C2C2-	ahy.1W91V	nner.gnm1	303	43	-2.804
ner.gnm1.ar	C2C2-Dof	TF	2->C2C2-	ahy.2GK99	nner.gnm1	24	0	-5.909
ner.gnm1.a	C2C2-Dof	TF	2->C2C2-	ahy.34G27	nner.gnm1	164	13	-3.719
ner.gnm1.ar	C2C2-Dof	TF	2->C2C2-	ahy.4PTW9	nner.gnm1	33	1	-5.467
ner.gnm1.ar	C2C2-Dof	TF	2->C2C2-	ahy.5HWL	nner.gnm1	180	371	1.046
ner.gnm1.a	C2C2-Dof	TF	2->C2C2-	ahy.8CQX	nner.gnm1	113	11	-3.341
ner.gnm1.ar	C2C2-Dof	TF	2->C2C2-	ahy.90U3D	nner.gnm1	9	0	-5.514
ner.gnm1.ar	C2C2-Dof	TF	2->C2C2-	ahy.915EM	nner.gnm1	44	91	1.054
ner.gnm1.a	C2C2-Dof	TF	2->C2C2-	ahy.9D5CI	nner.gnm1	30	3	-3.384
ner.gnm1.ar	C2C2-Dof	TF	2->C2C2-	ahy.9H8X3	nner.gnm1	82	34	-1.263
ner.gnm1.ar	C2C2-Dof	TF	2->C2C2-	ahy.E40HC	nner.gnm1	66	16	-2.062
ner.gnm1.a	C2C2-Dof	TF	2->C2C2-	ahy.FCS6C	nner.gnm1	17	72	2.134
ner.gnm1.ar	C2C2-Dof	TF	2->C2C2-	hy.K8YLA	nner.gnm1	391	13	-4.9
ner.gnm1.a	C2C2-Dof	TF	2->C2C2-	ahy.ME2J2	nner.gnm1	57	5	-3.443
ner.gnm1.ar	C2C2-Dof	TF	2->C2C2-	hy.MNZ0	nner.gnm1	128	356	1.472
ner.gnm1.ar	C2C2-Dof	TF	2->C2C2-	ahy.NQ5K	nner.gnm1	23	90	1.964
ner.gnm1.a	C2C2-Dof	TF	2->C2C2-	ahy.TL4KI	nner.gnm1	64	217	1.76

ıer.gnm1.ar	C2C2-Dof	TF	22->C2C2-	ahy.V6RQ(	ınner.gnm1	309	117	-1.399
ıer.gnm1.ar	C2C2-Dof	TF	22->C2C2-	ahy.VNR2(	ınner.gnm1	56	20	-1.468
ıer.gnm1.ar	C2C2-Dof	TF	22->C2C2-	ahy.Y4ERN	ınner.gnm1	154	60	-1.356
ner.gnm1.a	C2C2-Dof	TF	22->C2C2-	ahy.ZL8RI	ınner.gnm1	745	1660	1.155
ner.gnm1.a	22C2-GAT.	TF	2->C2C2-G	ahy.3J6WS	ınner.gnm1	7	0	-5.128
ner.gnm1.a	22C2-GAT.	TF	2->C2C2-G	ahy.3S8P0	ınner.gnm1	1001	3065	1.615
ner.gnm1.a	22C2-GAT.	TF	2->C2C2-G	ahy.4Y7J5	ınner.gnm1	104	20	-2.343
ıer.gnm1.ar	22C2-GAT.	TF	2->C2C2-G	ahy.GUPV5	ınner.gnm1	98	15	-2.736
ıer.gnm1.ar	22C2-GAT.	TF	2->C2C2-G	ahy.HHX0	ınner.gnm1	116	9	-3.6
ner.gnm1.a	22C2-GAT.	TF	2->C2C2-G	ahy.I6JZD	ınner.gnm1	1250	3229	1.369
ner.gnm1.a	22C2-GAT.	TF	2->C2C2-G	ahy.KA6Y.	ınner.gnm1	129	53	-1.282
ıer.gnm1.ar	22C2-GAT.	TF	2->C2C2-G	ahy.NCU4I	ınner.gnm1	25	3	-3.124
ıer.gnm1.a	2C2-YABE	TF	->C2C2-Y	ahy.3KF6F	ınner.gnm1	0	9	4.764
ner.gnm1.a	C2H2	TF	C2H2	ahy.0S1LA	ınner.gnm1	282	116	-1.28
ner.gnm1.a	C2H2	TF	C2H2	ahy.20D4K	ınner.gnm1	41	146	1.831
ner.gnm1.a	C2H2	TF	C2H2	ahy.2IZ1H	ınner.gnm1	12	73	2.578
ner.gnm1.a	C2H2	TF	C2H2	ahy.30LJX	ınner.gnm1	126	62	-1.032
ıer.gnm1.a	C2H2	TF	C2H2	ahy.32BR6	ınner.gnm1	234	2	-6.731
ner.gnm1.a	C2H2	TF	C2H2	ahy.369ZB	ınner.gnm1	110	37	-1.56
ner.gnm1.a	C2H2	TF	C2H2	ahy.3E1TE	ınner.gnm1	11	98	3.221
ıer.gnm1.ar	C2H2	TF	C2H2	ahy.53HSK	ınner.gnm1	46	176	1.95
ıer.gnm1.a	C2H2	TF	C2H2	ahy.55B6K	ınner.gnm1	74	737	3.321
ner.gnm1.a	C2H2	TF	C2H2	ahy.5FCQ	ınner.gnm1	229	14	-4.04
ıer.gnm1.ar	C2H2	TF	C2H2	ahy.60VM	ınner.gnm1	310	57	-2.446
ıer.gnm1.a	C2H2	TF	C2H2	ahy.6835Z	ınner.gnm1	528	222	-1.25
ner.gnm1.a	C2H2	TF	C2H2	ahy.860KU	ınner.gnm1	7	0	-5.085
ıer.gnm1.ar	C2H2	TF	C2H2	ahy.8W2D	ınner.gnm1	1359	419	-1.699
ner.gnm1.a	C2H2	TF	C2H2	ahy.908HV	ınner.gnm1	150	22	-2.77
ıer.gnm1.ar	C2H2	TF	C2H2	ahy.A8Q4I	ınner.gnm1	292	36	-3.016
ner.gnm1.a	C2H2	TF	C2H2	ahy.B4LZ3	ınner.gnm1	229	12	-4.199
ıer.gnm1.ar	C2H2	TF	C2H2	ahy.BAL5E	ınner.gnm1	468	1116	1.253
ıer.gnm1.a	C2H2	TF	C2H2	ahy.CA07E	ınner.gnm1	63	362	2.538
ıer.gnm1.a	C2H2	TF	C2H2	ahy.DX8F	ınner.gnm1	119	378	1.665
ıer.gnm1.ar	C2H2	TF	C2H2	ahy.GRPH	ınner.gnm1	4	24	2.746
ıer.gnm1.ar	C2H2	TF	C2H2	ahy.H7D8H	ınner.gnm1	404	97	-2.065
er.gnm1.ar	C2H2	TF	C2H2	ahy.H8QNV	ınner.gnm1	57	134	1.23
ner.gnm1.a	C2H2	TF	C2H2	ahy.IVEQ2	ınner.gnm1	363	982	1.437
ıer.gnm1.a	C2H2	TF	C2H2	ahy.LUSX	ınner.gnm1	142	305	1.105
er.gnm1.ar	C2H2	TF	C2H2	ahy.LXQM	ınner.gnm1	359	127	-1.505
er.gnm1.an	C2H2	TF	C2H2	hy.MH5U\	ınner.gnm1	185	403	1.125
ıer.gnm1.ar	C2H2	TF	C2H2	ahy.N4UY	ınner.gnm1	103	11	-3.236
ner.gnm1.a	C2H2	TF	C2H2	ahy.N8H2	ınner.gnm1	67	152	1.187
ıer.gnm1.ar	C2H2	TF	C2H2	ahy.QY5N(	ınner.gnm1	133	33	-2.033
ıer.gnm1.a	C2H2	TF	C2H2	ahy.T9EC(	ınner.gnm1	18	102	2.466
ner.gnm1.a	C2H2	TF	C2H2	ahy.TFH4	ınner.gnm1	17	1	-3.954

ner.gnm1.a	C2H2	TF	C2H2	ahy.U3PP5	inner.gnm1	163	538	1.727
ner.gnm1.ar	C2H2	TF	C2H2	ahy.UTHZ1	inner.gnm1	29	353	3.635
er.gnm1.an	C2H2	TF	C2H2	hy.WX8A0	inner.gnm1	24	3	-2.781
ner.gnm1.a	C2H2	TF	C2H2	ahy.YDP8I	inner.gnm1	377	84	-2.176
ner.gnm1.a	C2H2	TF	C2H2	ahy.YVFI3	inner.gnm1	4	97	4.444
ner.gnm1.ar	C3H	TF	C3H	ahy.S57KM	nner.gnm1.	94	19	-2.302
ner.gnm1.ar	C3H	TF	C3H	ahy.W4V2I	inner.gnm1	19	136	2.831
er.gnm1.ar	C3H	TF	C3H	hy.WU4X(	inner.gnm1	7	75	3.436
er.gnm1.an	C3H	TF	C3H	hy.YQ2HF	inner.gnm1	170	419	1.3
ner.gnm1.ar	CPP	TF	CPP	ahy.VMK2	inner.gnm1	157	30	-2.376
ner.gnm1.a	DBB	TF	DBB	ahy.3FT6Z	inner.gnm1	1083	458	-1.242
ner.gnm1.a	DBB	TF	DBB	ahy.PY0P2	inner.gnm1	409	116	-1.824
ner.gnm1.ar	DBB	TF	DBB	ahy.R1XH/	inner.gnm1	587	193	-1.609
er.gnm1.an	DBP	TF	DBP	hy.WG32V	inner.gnm1	182	4	-5.405
ner.gnm1.a	E2F-DP	TF	E2F-DP	ahy.2L64Q	inner.gnm1	14	1	-3.672
ner.gnm1.a	E2F-DP	TF	E2F-DP	ahy.C6R17	inner.gnm1	19	0	-6.54
ner.gnm1.a	E2F-DP	TF	E2F-DP	ahy.Q67Z2	inner.gnm1	471	209	-1.178
ner.gnm1.a	FAR1	TF	FAR1	ahy.8SNE7	inner.gnm1	10	31	1.649
ner.gnm1.ar	ARP-ARR-	TF	->GARP-A	hy.Q3RA0	inner.gnm1	36	6	-2.635
er.gnm1.ar	ARP-ARR-	TF	->GARP-A	hy.UG2HI	inner.gnm1	66	25	-1.371
ner.gnm1.a	ARP-G2-li	TF	->GARP-G	ahy.1J8Q1	inner.gnm1	39	11	-1.798
ner.gnm1.a	ARP-G2-li	TF	->GARP-G	ahy.1Z7G0	inner.gnm1	960	254	-1.921
ner.gnm1.a	ARP-G2-li	TF	->GARP-G	ahy.25X10	inner.gnm1	207	89	-1.219
ner.gnm1.ar	ARP-G2-li	TF	->GARP-G	hy.A9PKF	inner.gnm1	393	1	-8.09
ner.gnm1.a	ARP-G2-li	TF	->GARP-G	ahy.DD0T0	inner.gnm1	588	257	-1.191
ner.gnm1.ar	ARP-G2-li	TF	->GARP-G	ahy.G8T7F	inner.gnm1	281	749	1.418
er.gnm1.an	ARP-G2-li	TF	->GARP-G	hy.GRY8C	inner.gnm1	1489	490	-1.605
ner.gnm1.a	ARP-G2-li	TF	->GARP-G	rahy.J44A6	inner.gnm1	156	48	-1.688
ner.gnm1.a	ARP-G2-li	TF	->GARP-G	ahy.KD5B	inner.gnm1	116	36	-1.715
er.gnm1.ar	ARP-G2-li	TF	->GARP-G	hy.MQY6	inner.gnm1	1439	489	-1.558
ner.gnm1.ar	ARP-G2-li	TF	->GARP-G	ahy.N0RGI	inner.gnm1	48	2	-4.433
ner.gnm1.ar	ARP-G2-li	TF	->GARP-G	hy.N8Q6V	inner.gnm1	535	211	-1.342
ner.gnm1.a	ARP-G2-li	TF	->GARP-G	ahy.QX8C	inner.gnm1	312	88	-1.827
ner.gnm1.a	ARP-G2-li	TF	->GARP-G	ahy.SI8X9	inner.gnm1	25	0	-6.937
ner.gnm1.a	ARP-G2-li	TF	->GARP-G	rahy.SPR8	inner.gnm1	85	4	-4.569
ner.gnm1.a	ARP-G2-li	TF	->GARP-G	ahy.UB10Y	inner.gnm1	532	197	-1.435
ner.gnm1.ar	ARP-G2-li	TF	->GARP-G	hy.V5WQ	inner.gnm1	15	89	2.6
ner.gnm1.a	ARP-G2-li	TF	->GARP-G	ahy.VJ5Q	inner.gnm1	331	105	-1.653
ner.gnm1.ar	ARP-G2-li	TF	->GARP-G	hy.VRF1C	inner.gnm1	513	1181	1.204
ner.gnm1.a	ARP-G2-li	TF	->GARP-G	ahy.Y2U0	inner.gnm1	1004	402	-1.322
ner.gnm1.a	ARP-G2-li	TF	->GARP-G	ahy.YIX46	inner.gnm1	791	158	-2.319
er.gnm1.ar	ARP-G2-li	TF	->GARP-G	hy.ZMGM	inner.gnm1	1211	266	-2.188
ner.gnm1.a	GNAT	TR	GNAT	rahy.15S67	inner.gnm1	1	62	5.768
ner.gnm1.a	GNAT	TR	GNAT	ahy.2AD11	inner.gnm1	3	23	2.805
ner.gnm1.a	GNAT	TR	GNAT	ahy.43GZ2	inner.gnm1	2	19	3.389

ıer.gnm1.a	GNAT	TR	GNAT	ahy.5V2BX	ınner.gnm1	73	12	-2.627
ıer.gnm1.aı	GNAT	TR	GNAT	ahy.FQMP	ınner.gnm1	38	8	-2.232
ıer.gnm1.aı	GNAT	TR	GNAT	ahy.GCG1	ınner.gnm1	43	154	1.865
ıer.gnm1.aı	GNAT	TR	GNAT	ahy.ML1PU	ınner.gnm1	26	67	1.388
ıer.gnm1.aı	GNAT	TR	GNAT	ahy.NU1P8	ınner.gnm1	158	57	-1.482
ıer.gnm1.aı	GNAT	TR	GNAT	ahy.Q2LGC	ınner.gnm1	204	61	-1.728
er.gnm1.an	GNAT	TR	GNAT	hy.RYXG2	ınner.gnm1	95	612	2.69
ıer.gnm1.aı	GNAT	TR	GNAT	ahy.V4ADI	ınner.gnm1	1	139	6.879
ıer.gnm1.a	GNAT	TR	GNAT	ahy.XZ9N	ınner.gnm1	19	141	2.915
er.gnm1.ar	GRAS	TF	GRAS	ıhy.1BB2M	ınner.gnm1	3503	1398	-1.325
ıer.gnm1.aı	GRAS	TF	GRAS	ahy.8X00Q	ınner.gnm1	296	95	-1.634
er.gnm1.ar	GRAS	TF	GRAS	ıhy.M96W	ınner.gnm1	1116	521	-1.098
ıer.gnm1.a	GRAS	TF	GRAS	ahy.P9UX8	ınner.gnm1	263	90	-1.543
ner.gnm1.a	GRAS	TF	GRAS	ahy.YX6J2	ınner.gnm1	3	33	3.678
ner.gnm1.a	GRF	TF	GRF	ahy.727QE	ınner.gnm1	81	34	-1.247
ıer.gnm1.aı	GRF	TF	GRF	ahy.7VQ11	ınner.gnm1	117	11	-3.376
ıer.gnm1.aı	GRF	TF	GRF	ahy.AF8AI	ınner.gnm1	34	7	-2.381
ner.gnm1.a	GRF	TF	GRF	ahy.E8HE7	ınner.gnm1	84	14	-2.602
ıer.gnm1.aı	GRF	TF	GRF	ahy.EY9JV	ınner.gnm1	61	10	-2.591
ıer.gnm1.aı	GRF	TF	GRF	ahy.G4PR1	ınner.gnm1	139	54	-1.374
ner.gnm1.a	GRF	TF	GRF	ahy.IE1EN	ınner.gnm1	124	10	-3.617
er.gnm1.ar	GRF	TF	GRF	ıhy.KV1X6	ınner.gnm1	125	24	-2.405
er.gnm1.an	GRF	TF	GRF	hy.MSLW	ınner.gnm1	112	38	-1.545
ıer.gnm1.aı	GRF	TF	GRF	ahy.N7G5L	ınner.gnm1	49	9	-2.487
ıer.gnm1.aı	HB-BELL	TF	3->HB-BE	ahy.2C6XE	ınner.gnm1	133	404	1.605
er.gnm1.ar	HB-BELL	TF	3->HB-BE	ıhy.4HW5A	ınner.gnm1	33	1	-4.951
ıer.gnm1.aı	HB-BELL	TF	3->HB-BE	ahy.69CRN	ınner.gnm1	115	236	1.04
ner.gnm1.a	HB-HD-ZII	TF	->HB-HD-	ahy.1QJ78	ınner.gnm1	70	2	-5.25
ner.gnm1.a	HB-HD-ZII	TF	->HB-HD-	ahy.29D94	ınner.gnm1	275	101	-1.438
ıer.gnm1.a	HB-HD-ZII	TF	->HB-HD-	ahy.43GLK	ınner.gnm1	847	212	-1.999
ıer.gnm1.a	HB-HD-ZII	TF	->HB-HD-	ahy.5G8LZ	ınner.gnm1	866	1999	1.207
ıer.gnm1.a	HB-HD-ZII	TF	->HB-HD-	ahy.69QRI	ınner.gnm1	252	67	-1.917
ıer.gnm1.a	HB-HD-ZII	TF	->HB-HD-	ahy.71NB6	ınner.gnm1	85	28	-1.597
ner.gnm1.a	HB-HD-ZII	TF	->HB-HD-	ahy.899DF	ınner.gnm1	45	4	-3.328
ıer.gnm1.a	HB-HD-ZII	TF	->HB-HD-	ahy.97AX4	ınner.gnm1	513	222	-1.212
ıer.gnm1.a	HB-HD-ZII	TF	->HB-HD-	ahy.BYTU	ınner.gnm1	265	129	-1.044
ıer.gnm1.a	HB-HD-ZII	TF	->HB-HD-	ahy.EBSD6	ınner.gnm1	567	1480	1.384
ner.gnm1.a	HB-HD-ZII	TF	->HB-HD-	ahy.FYS41	ınner.gnm1	0	10	5.865
ner.gnm1.a	HB-HD-ZII	TF	->HB-HD-	ahy.J7P0L	ınner.gnm1	20	113	2.531
ner.gnm1.a	HB-HD-ZII	TF	->HB-HD-	ahy.JCYU	ınner.gnm1	1091	180	-2.601
ıer.gnm1.a	HB-HD-ZII	TF	->HB-HD-	ahy.NX7S1	ınner.gnm1	406	1625	2.003
ıer.gnm1.a	HB-HD-ZII	TF	->HB-HD-	ahy.QL12S	ınner.gnm1	600	1227	1.033
er.gnm1.ar	HB-HD-ZII	TF	->HB-HD-	ıhy.RWXL	ınner.gnm1	100	43	-1.213
ıer.gnm1.a	HB-HD-ZII	TF	->HB-HD-	ahy.VK2HI	ınner.gnm1	608	278	-1.132
er.gnm1.ar	HB-HD-ZII	TF	->HB-HD-	ıhy.VQN41	ınner.gnm1	38	3	-3.689

ıer.gnm1.aı	HB-HD-ZII	TF	->HB-HD-	ahy.VX60J	ınner.gnm1	436	114	-1.93
er.gnm1.ar	HB-HD-ZII	TF	->HB-HD-	ıhy.W0PH	ınner.gnm1	230	715	1.639
ıer.gnm1.aı	HB-HD-ZII	TF	->HB-HD-	ahy.YS1R7	ınner.gnm1	1300	82	-3.998
ıer.gnm1.a	HB-HD-ZII	TF	->HB-HD-	ahy.ZBAY	ınner.gnm1	225	452	1.01
ıer.gnm1.aı	HB-KNOX	TF	3->HB-KN	ahy.4NTH	ınner.gnm1	2286	4581	1.003
ıer.gnm1.aı	HB-KNOX	TF	3->HB-KN	ahy.EUX0	ınner.gnm1	0	9	5.815
ıer.gnm1.aı	HB-KNOX	TF	3->HB-KN	ahy.F9ETH	ınner.gnm1	128	63	-1.029
ıer.gnm1.aı	HB-KNOX	TF	3->HB-KN	ıhy.FN3HA	ınner.gnm1	44	0	-7.753
ıer.gnm1.aı	HB-KNOX	TF	3->HB-KN	ahy.U5Q1C	ınner.gnm1	396	90	-2.134
ıer.gnm1.aı	HB-KNOX	TF	3->HB-KN	ahy.Z81W	ınner.gnm1	23	0	-6.823
er.gnm1.an	HB-other	TF	B->HB-oth	ıhy.12HW	ınner.gnm1	2182	34	-5.995
ıer.gnm1.a	HB-other	TF	B->HB-oth	ahy.6BR0C	ınner.gnm1	188	386	1.042
ner.gnm1.a	HB-other	TF	B->HB-oth	ahy.KEIQ	ınner.gnm1	1320	11	-6.91
ıer.gnm1.aı	HB-WOX	TF	3->HB-WC	ıhy.1P3WH	ınner.gnm1	17	50	1.56
ıer.gnm1.aı	HB-WOX	TF	3->HB-WC	ahy.9WG3	ınner.gnm1	41	330	3.012
ıer.gnm1.a	HB-WOX	TF	3->HB-WC	ahy.H61EE	ınner.gnm1	75	10	-2.854
ıer.gnm1.aı	HB-WOX	TF	3->HB-WC	ıhy.LR4Q	ınner.gnm1	33	0	-7.378
er.gnm1.ar	HB-WOX	TF	3->HB-WC	ıhy.WG7T	ınner.gnm1	1	52	5.472
ıer.gnm1.a	HMG	TR	HMG	ahy.1D56Y	ınner.gnm1	168	9	-4.195
ıer.gnm1.aı	HMG	TR	HMG	ahy.BLWT	ınner.gnm1	197	11	-4.151
ner.gnm1.a	HMG	TR	HMG	rahy.Y783	ınner.gnm1	170	2	-6.839
ner.gnm1.a	HMG	TR	HMG	ahy.Z3R5C	ınner.gnm1	264	7	-5.17
ner.gnm1.a	HSF	TF	HSF	rahy.4L43E	ınner.gnm1	1115	437	-1.351
ıer.gnm1.a	HSF	TF	HSF	ahy.7EA4E	ınner.gnm1	147	0	-9.515
ıer.gnm1.aı	HSF	TF	HSF	ahy.7H2V	ınner.gnm1	149	17	-3.126
ıer.gnm1.aı	HSF	TF	HSF	ahy.8U41W	ınner.gnm1	649	1884	1.538
ıer.gnm1.aı	HSF	TF	HSF	ahy.B8VX	ınner.gnm1	495	88	-2.499
ner.gnm1.a	HSF	TF	HSF	rahy.FJH25	ınner.gnm1	147	571	1.954
ıer.gnm1.a	HSF	TF	HSF	ahy.FTRM	ınner.gnm1	815	157	-2.376
er.gnm1.ar	HSF	TF	HSF	ıhy.M7PLV	ınner.gnm1	60	0	-8.217
ıer.gnm1.a	HSF	TF	HSF	ahy.W19Z	ınner.gnm1	409	1256	1.62
ıer.gnm1.a	IWS1	TR	IWS1	ahy.SF5C	ınner.gnm1	1479	730	-1.02
ıer.gnm1.a	IWS1	TR	IWS1	ahy.TK2Q	ınner.gnm1	1704	769	-1.148
ıer.gnm1.a	Jumonji	TR	Jumonji	ahy.9S6A	ınner.gnm1	584	267	-1.128
er.gnm1.ar	Jumonji	TR	Jumonji	ıhy.H9D2V	ınner.gnm1	1976	5694	1.527
ıer.gnm1.aı	Jumonji	TR	Jumonji	ahy.JYC97	ınner.gnm1	3913	1631	-1.263
ner.gnm1.a	Jumonji	TR	Jumonji	rahy.Y876J	ınner.gnm1	1316	3234	1.298
ıer.gnm1.a	LIM	TF	LIM	ahy.DE3F1	ınner.gnm1	82	7	-3.499
ner.gnm1.a	LIM	TF	LIM	rahy.VFJ7H	ınner.gnm1	6	0	-5.004
ıer.gnm1.a	LOB	TF	LOB	ahy.54E1W	ınner.gnm1	10	173	4.189
ner.gnm1.a	LOB	TF	LOB	rahy.8GTF	ınner.gnm1	170	386	1.18
ıer.gnm1.a	LOB	TF	LOB	ahy.A5HF	ınner.gnm1	12	1	-3.974
ıer.gnm1.aı	LOB	TF	LOB	ıhy.C3HPV	ınner.gnm1	1367	532	-1.361
er.gnm1.ar	LOB	TF	LOB	ıhy.DCW7	ınner.gnm1	209	501	1.262
ıer.gnm1.a	LOB	TF	LOB	ahy.HZIC2	ınner.gnm1	98	43	-1.178

ıer.gnm1.aı	LOB	TF	LOB	ıhy.KN2B7	ınner.gnm1	6	0	-4.784
ner.gnm1.a	LOB	TF	LOB	ahy.LBX2(	ınner.gnm1	16	0	-6.299
er.gnm1.an	LOB	TF	LOB	hy.MBY2C	ınner.gnm1	39	10	-1.923
ner.gnm1.a	LOB	TF	LOB	ahy.SEL59	ınner.gnm1	43	1	-4.88
ner.gnm1.a	LOB	TF	LOB	ahy.WEI72	ınner.gnm1	16	264	4.07
ıer.gnm1.aı	LOB	TF	LOB	ıhy.XFT5L	ınner.gnm1	60	1	-6.388
ner.gnm1.aı	LOB	TF	LOB	ahy.Z62C6	ınner.gnm1	1183	439	-1.428
ner.gnm1.a	ADS-MIK	TF	S->MADS-	ahy.438FZ	ınner.gnm1	185	697	1.915
ner.gnm1.a	ADS-MIK	TF	S->MADS-	ahy.8A3K	ınner.gnm1	96	550	2.519
ıer.gnm1.a	ADS-MIK	TF	S->MADS-	ahy.D4F11	ınner.gnm1	248	674	1.444
ner.gnm1.a	ADS-MIK	TF	S->MADS-	ahy.IQR3N	ınner.gnm1	145	1060	2.871
er.gnm1.ar	ADS-MIK	TF	S->MADS-	ıhy.MX0V	ınner.gnm1	163	796	2.287
er.gnm1.ar	ADS-MIK	TF	S->MADS-	ıhy.QB26W	ınner.gnm1	541	1454	1.427
ner.gnm1.a	ADS-MIK	TF	S->MADS-	ahy.SC2RC	ınner.gnm1	125	410	1.722
ıer.gnm1.a	ADS-MIK	TF	S->MADS-	ahy.SLJ44	ınner.gnm1	2475	5637	1.187
er.gnm1.ar	ADS-MIK	TF	S->MADS-	ıhy.WIWA	ınner.gnm1	165	618	1.904
ıer.gnm1.a	ADS-MIK	TF	S->MADS-	ahy.Z4CR3	ınner.gnm1	457	1503	1.717
ner.gnm1.a	ADS-M-ty	TF	->MADS-	ahy.5M652	ınner.gnm1	0	16	6.561
ıer.gnm1.a	ADS-M-ty	TF	->MADS-	ahy.GD5Y	ınner.gnm1	185	41	-2.174
ner.gnm1.a	ADS-M-ty	TF	->MADS-	rahy.PIZ1S	ınner.gnm1	113	52	-1.141
er.gnm1.ar	ADS-M-ty	TF	->MADS-	ıhy.TW1RI	ınner.gnm1	197	618	1.655
ıer.gnm1.aı	MBF1	TR	MBF1	ahy.1CR6H	ınner.gnm1	1780	3648	1.035
ner.gnm1.a	MBF1	TR	MBF1	rahy.B1IT4	ınner.gnm1	1171	2511	1.101
ıer.gnm1.aı	mTERF	TR	mTERF	ahy.0G4LN	ınner.gnm1	70	24	-1.552
er.gnm1.ar	mTERF	TR	mTERF	ıhy.AYWF	ınner.gnm1	125	62	-1.012
er.gnm1.ar	mTERF	TR	mTERF	ıhy.M5TQI	ınner.gnm1	57	25	-1.187
ıer.gnm1.aı	mTERF	TR	mTERF	ahy.SB3XC	ınner.gnm1	364	70	-2.378
ner.gnm1.aı	mTERF	TR	mTERF	ahy.WZ54I	ınner.gnm1	384	181	-1.085
ıer.gnm1.a	MYB	TF	IYB->MY	ahy.02XPC	ınner.gnm1	462	28	-4.06
ner.gnm1.a	MYB	TF	IYB->MY	ahy.04JND	ınner.gnm1	880	322	-1.449
ner.gnm1.a	MYB	TF	IYB->MY	ahy.0KDF	ınner.gnm1	291	53	-2.458
ner.gnm1.a	MYB	TF	IYB->MY	ahy.12J3V	ınner.gnm1	257	79	-1.703
ner.gnm1.a	MYB	TF	IYB->MY	ahy.28HN	ınner.gnm1	11	0	-4.752
ıer.gnm1.aı	MYB	TF	IYB->MY	ıhy.2BK3K	ınner.gnm1	580	100	-2.533
ıer.gnm1.a	MYB	TF	IYB->MY	ahy.2DN1	ınner.gnm1	11	1	-3.897
ıer.gnm1.a	MYB	TF	IYB->MY	ahy.2DQF	ınner.gnm1	146	33	-2.16
ıer.gnm1.a	MYB	TF	IYB->MY	ahy.55CRI	ınner.gnm1	97	12	-2.963
ıer.gnm1.aı	MYB	TF	IYB->MY	ahy.57FM2	ınner.gnm1	30	10	-1.486
ıer.gnm1.aı	MYB	TF	IYB->MY	ıhy.A2IWI	ınner.gnm1	503	64	-2.986
ıer.gnm1.aı	MYB	TF	IYB->MY	ıhy.ARS1I	ınner.gnm1	142	46	-1.629
ıer.gnm1.a	MYB	TF	IYB->MY	ahy.DPKT	ınner.gnm1	223	95	-1.232
er.gnm1.an	MYB	TF	IYB->MY	ıhy.DV7Q	ınner.gnm1	1015	420	-1.273
ner.gnm1.a	MYB	TF	IYB->MY	rahy.EJIH9	ınner.gnm1	108	35	-1.646
ıer.gnm1.a	MYB	TF	IYB->MY	ahy.FS3AI	ınner.gnm1	571	284	-1.007
ıer.gnm1.a	MYB	TF	IYB->MY	ahy.H8DJI	ınner.gnm1	158	18	-3.154

ner.gnm1.a	MYB	TF	MYB->MY	ahy.I2L3K	nner.gnm1	25	115	2.163
ner.gnm1.a	MYB	TF	MYB->MY	ahy.J3K16	nner.gnm1	21	0	-6.709
ner.gnm1.a	MYB	TF	MYB->MY	ahy.LQRE	nner.gnm1	5	26	2.363
er.gnm1.an	MYB	TF	MYB->MY	hy.MN60M	nner.gnm1	5	0	-4.675
ner.gnm1.a	MYB	TF	MYB->MY	ahy.N15QI	nner.gnm1	74	5	-3.863
ner.gnm1.a	MYB	TF	MYB->MY	ahy.PR7AY	nner.gnm1	324	12	-4.721
ner.gnm1.a	MYB	TF	MYB->MY	ahy.QF6X9	nner.gnm1	162	52	-1.633
ner.gnm1.a	MYB	TF	MYB->MY	ahy.QZK5	nner.gnm1	131	306	1.233
er.gnm1.an	MYB	TF	MYB->MY	hy.R9MXI	nner.gnm1	2	38	4.313
er.gnm1.an	MYB	TF	MYB->MY	hy.S8NMI	nner.gnm1	891	59	-3.919
ner.gnm1.a	MYB	TF	MYB->MY	ahy.SJGE2	nner.gnm1	793	20	-5.308
ner.gnm1.a	MYB	TF	MYB->MY	ahy.T1CM	nner.gnm1	548	229	-1.263
ner.gnm1.a	MYB	TF	MYB->MY	ahy.US2SK	nner.gnm1	306	36	-3.08
er.gnm1.an	MYB	TF	MYB->MY	hy.UX5VI	nner.gnm1	132	265	1.002
ner.gnm1.a	MYB	TF	MYB->MY	ahy.W3P5I	nner.gnm1	505	172	-1.555
er.gnm1.an	MYB	TF	MYB->MY	hy.W7ZT	nner.gnm1	1043	462	-1.175
er.gnm1.an	MYB	TF	MYB->MY	hy.WMRY	nner.gnm1	86	217	1.327
er.gnm1.an	MYB	TF	MYB->MY	hy.WSM8	nner.gnm1	518	16	-4.996
ner.gnm1.a	MYB	TF	MYB->MY	ahy.Y8Q1I	nner.gnm1	55	121	1.147
ner.gnm1.a	MYB	TF	MYB->MY	ahy.YFKH	nner.gnm1	715	1636	1.195
ner.gnm1.a	MYB	TF	MYB->MY	ahy.YX3IC	nner.gnm1	649	242	-1.422
ner.gnm1.a	MYB	TF	MYB->MY	ahy.ZZ8D5	nner.gnm1	255	124	-1.042
ner.gnm1.a	MYB-relate	TF	3->MYB-re	ahy.006X4	nner.gnm1	136	40	-1.751
ner.gnm1.a	MYB-relate	TF	3->MYB-re	ahy.18CGF	nner.gnm1	358	135	-1.4
ner.gnm1.a	MYB-relate	TF	3->MYB-re	ahy.29NKI	nner.gnm1	39	1878	5.603
ner.gnm1.a	MYB-relate	TF	3->MYB-re	ahy.2A56X	nner.gnm1	28	122	2.113
ner.gnm1.a	MYB-relate	TF	3->MYB-re	ahy.3B72J	nner.gnm1	35	83	1.232
ner.gnm1.a	MYB-relate	TF	3->MYB-re	ahy.53JK2	nner.gnm1	64	12	-2.454
ner.gnm1.a	MYB-relate	TF	3->MYB-re	ahy.53Z0F	nner.gnm1	12	0	-5.845
ner.gnm1.a	MYB-relate	TF	3->MYB-re	ahy.6ZYP	nner.gnm1	789	142	-2.48
ner.gnm1.a	MYB-relate	TF	3->MYB-re	ahy.7P5JV	nner.gnm1	57	1	-5.281
ner.gnm1.a	MYB-relate	TF	3->MYB-re	ahy.B03SC	nner.gnm1	13	40	1.599
ner.gnm1.a	MYB-relate	TF	3->MYB-re	ahy.BFP4V	nner.gnm1	22	3	-2.745
ner.gnm1.a	MYB-relate	TF	3->MYB-re	ahy.C0LG	nner.gnm1	8	0	-5.346
ner.gnm1.a	MYB-relate	TF	3->MYB-re	ahy.CD1P	nner.gnm1	606	37	-4.041
ner.gnm1.a	MYB-relate	TF	3->MYB-re	ahy.FP3AS	nner.gnm1	31	1	-5.388
ner.gnm1.a	MYB-relate	TF	3->MYB-re	ahy.H5DQ	nner.gnm1	134	9	-3.818
ner.gnm1.a	MYB-relate	TF	3->MYB-re	ahy.H8IXF	nner.gnm1	13	0	-5.068
ner.gnm1.a	MYB-relate	TF	3->MYB-re	ahy.HB1R	nner.gnm1	128	32	-1.985
ner.gnm1.a	MYB-relate	TF	3->MYB-re	ahy.IEZ2L	nner.gnm1	6	0	-4.931
ner.gnm1.a	MYB-relate	TF	3->MYB-re	ahy.K64R	nner.gnm1	52	833	4.008
ner.gnm1.a	MYB-relate	TF	3->MYB-re	ahy.L85IH	nner.gnm1	313	809	1.369
er.gnm1.an	MYB-relate	TF	3->MYB-re	hy.LQ8VV	nner.gnm1	517	56	-3.216
ner.gnm1.a	MYB-relate	TF	3->MYB-re	ahy.NV91I	nner.gnm1	225	16	-3.834
ner.gnm1.a	MYB-relate	TF	3->MYB-re	ahy.NXJT	nner.gnm1	40	4	-3.184

ner.gnm1.a	MYB-relate	TF	3->MYB-re	ahy.R24AV	inner.gnm1	146	62	-1.234
ner.gnm1.a	MYB-relate	TF	3->MYB-re	ahy.RX2H	inner.gnm1	106	14	-2.924
ner.gnm1.a	MYB-relate	TF	3->MYB-re	ahy.S1X8C	inner.gnm1	941	436	-1.113
er.gnm1.an	MYB-relate	TF	3->MYB-re	hy.T7BW	inner.gnm1	1546	613	-1.335
er.gnm1.an	MYB-relate	TF	3->MYB-re	hy.U5RW	inner.gnm1	35	79	1.162
er.gnm1.an	MYB-relate	TF	3->MYB-re	hy.WHG5	inner.gnm1	1410	339	-2.056
ner.gnm1.a	MYB-relate	TF	3->MYB-re	ahy.XL1L	inner.gnm1	887	44	-4.337
ner.gnm1.a	MYB-relate	TF	3->MYB-re	ahy.XL6S	inner.gnm1	57	5	-3.379
ner.gnm1.a	NAC	TF	NAC	ahy.05SHI	inner.gnm1	10	0	-4.613
ner.gnm1.a	NAC	TF	NAC	ahy.0V68X	inner.gnm1	146	677	2.212
er.gnm1.an	NAC	TF	NAC	hy.12HUM	inner.gnm1	402	1487	1.888
ner.gnm1.a	NAC	TF	NAC	ahy.1GPE(	inner.gnm1	9	0	-5.388
ner.gnm1.a	NAC	TF	NAC	rahy.1I167	inner.gnm1	18	111	2.636
ner.gnm1.a	NAC	TF	NAC	ahy.1UE6	inner.gnm1	3367	7477	1.151
ner.gnm1.a	NAC	TF	NAC	ahy.2MPS	inner.gnm1	768	2731	1.83
ner.gnm1.a	NAC	TF	NAC	rahy.2P3Z	inner.gnm1	0	7	5.392
ner.gnm1.a	NAC	TF	NAC	rahy.3F2LJ	inner.gnm1	87	178	1.031
ner.gnm1.a	NAC	TF	NAC	rahy.5P3U	inner.gnm1	3	98	5.33
ner.gnm1.a	NAC	TF	NAC	ahy.76LAE	inner.gnm1	7	102	3.958
ner.gnm1.a	NAC	TF	NAC	ahy.7G7SI	inner.gnm1	149	321	1.109
ner.gnm1.a	NAC	TF	NAC	rahy.8E68Z	inner.gnm1	546	2128	1.964
ner.gnm1.a	NAC	TF	NAC	ahy.8GCY	inner.gnm1	166	747	2.168
ner.gnm1.a	NAC	TF	NAC	rahy.9732X	inner.gnm1	14	1	-3.229
ner.gnm1.a	NAC	TF	NAC	rahy.AIPG	inner.gnm1	351	43	-3.025
ner.gnm1.a	NAC	TF	NAC	ahy.B9XEI	inner.gnm1	26	692	4.715
ner.gnm1.a	NAC	TF	NAC	rahy.BEF3	inner.gnm1	14	2	-2.754
ner.gnm1.a	NAC	TF	NAC	ahy.BPCJ1	inner.gnm1	538	158	-1.767
ner.gnm1.a	NAC	TF	NAC	ahy.CDPA	inner.gnm1	545	156	-1.811
ner.gnm1.a	NAC	TF	NAC	ahy.D5FD	inner.gnm1	700	2529	1.855
ner.gnm1.a	NAC	TF	NAC	ahy.F01Q5	inner.gnm1	739	2903	1.974
ner.gnm1.a	NAC	TF	NAC	ahy.FD63A	inner.gnm1	24	4	-2.376
ner.gnm1.a	NAC	TF	NAC	ahy.GU1U	inner.gnm1	6	0	-4.907
ner.gnm1.a	NAC	TF	NAC	ahy.JHHH	inner.gnm1	102	36	-1.522
ner.gnm1.a	NAC	TF	NAC	rahy.K4491	inner.gnm1	133	42	-1.681
ner.gnm1.a	NAC	TF	NAC	ahy.L5HH	inner.gnm1	493	2059	2.065
er.gnm1.an	NAC	TF	NAC	hy.NB8KF	inner.gnm1	3755	1832	-1.036
ner.gnm1.a	NAC	TF	NAC	ahy.Q3Y7	inner.gnm1	525	2049	1.963
er.gnm1.an	NAC	TF	NAC	hy.QL5RC	inner.gnm1	10	113	3.491
er.gnm1.an	NAC	TF	NAC	hy.QVEY	inner.gnm1	22	176	3.019
ner.gnm1.a	NAC	TF	NAC	ahy.S9FEU	inner.gnm1	3290	213	-3.947
ner.gnm1.a	NAC	TF	NAC	rahy.SJ3Y3	inner.gnm1	6	0	-4.836
ner.gnm1.a	NAC	TF	NAC	ahy.T50EN	inner.gnm1	50	9	-2.549
er.gnm1.an	NAC	TF	NAC	hy.TY7W	inner.gnm1	560	2005	1.84
ner.gnm1.a	NAC	TF	NAC	ahy.UX5J	inner.gnm1	27	11	-1.32
ner.gnm1.a	NAC	TF	NAC	ahy.V0X4	inner.gnm1	10	1	-3.799

er.gnm1.ar	NAC	TF	NAC	ahy.V9YDI	ınner.gnm1	11	56	2.288
ner.gnm1.a	NAC	TF	NAC	ahy.XB6K	ınner.gnm1	16	787	5.57
ner.gnm1.a	NAC	TF	NAC	ahy.YY4A	ınner.gnm1	12	0	-4.885
ner.gnm1.a	NAC	TF	NAC	ahy.ZM1I0	ınner.gnm1	28	1	-4.278
ner.gnm1.ar	NF-YA	TF	F-Y->NF-Y	ahy.EWBI0	ınner.gnm1	27	4	-2.633
ner.gnm1.a	NF-YA	TF	F-Y->NF-Y	ahy.HITK0	ınner.gnm1	334	154	-1.118
er.gnm1.ar	NF-YA	TF	F-Y->NF-Y	ıhy.TY3GI	ınner.gnm1	417	172	-1.277
ner.gnm1.a	NF-YA	TF	F-Y->NF-Y	ahy.V1T15	ınner.gnm1	22	5	-2.332
ner.gnm1.a	NF-YB	TF	F-Y->NF-Y	ahy.8XPX	ınner.gnm1	361	32	-3.502
er.gnm1.ar	NF-YB	TF	F-Y->NF-Y	ıhy.BTE5M	ınner.gnm1	450	35	-3.708
ner.gnm1.a	NF-YC	TF	F-Y->NF-Y	ahy.F7CTI	ınner.gnm1	114	249	1.118
ner.gnm1.a	NF-YC	TF	F-Y->NF-Y	rahy.FIIU1	ınner.gnm1	276	1065	1.946
ner.gnm1.a	NF-YC	TF	F-Y->NF-Y	ahy.Y83U0	ınner.gnm1	19	58	1.583
ner.gnm1.a	NF-YC	TF	F-Y->NF-Y	ahy.Z93F9	ınner.gnm1	41	14	-1.517
ner.gnm1.a	Others	TR	Others	rahy.0MYI	ınner.gnm1	1004	398	-1.336
er.gnm1.ar	Others	TR	Others	ıhy.1U4M	ınner.gnm1	576	184	-1.646
ner.gnm1.a	Others	TR	Others	ahy.22FLX	ınner.gnm1	18	432	4.559
ner.gnm1.a	Others	TR	Others	rahy.4KS3	ınner.gnm1	963	194	-2.31
ner.gnm1.a	Others	TR	Others	ahy.64XMI	ınner.gnm1	31	12	-1.39
ner.gnm1.a	Others	TR	Others	ahy.8D0AI	ınner.gnm1	283	666	1.236
ner.gnm1.a	Others	TR	Others	rahy.91F6L	ınner.gnm1	114	47	-1.294
ner.gnm1.a	Others	TR	Others	ahy.AE0X9	ınner.gnm1	20	2	-3.219
ner.gnm1.a	Others	TR	Others	ahy.C3DR	ınner.gnm1	1418	659	-1.106
ner.gnm1.a	Others	TR	Others	rahy.D687J	ınner.gnm1	212	66	-1.672
ner.gnm1.a	Others	TR	Others	ahy.EU8XI	ınner.gnm1	8	37	2.237
ner.gnm1.a	Others	TR	Others	ahy.FQS2	ınner.gnm1	891	307	-1.538
ner.gnm1.a	Others	TR	Others	ahy.IH9T3	ınner.gnm1	435	1094	1.331
ner.gnm1.a	Others	TR	Others	rahy.IJP2R	ınner.gnm1	83	191	1.204
ner.gnm1.a	Others	TR	Others	ahy.IKLL4	ınner.gnm1	1	13	3.427
ner.gnm1.a	Others	TR	Others	ahy.IUYB4	ınner.gnm1	724	1464	1.016
ner.gnm1.a	Others	TR	Others	ahy.K5EF2	ınner.gnm1	48	12	-2.045
ner.gnm1.a	Others	TR	Others	ahy.Q1XF9	ınner.gnm1	64	16	-2.021
ner.gnm1.a	Others	TR	Others	ahy.QIR43	ınner.gnm1	386	193	-1.001
ner.gnm1.a	Others	TR	Others	ahy.TJ20X	ınner.gnm1	373	1144	1.618
ner.gnm1.a	Others	TR	Others	ahy.UU73	ınner.gnm1	587	107	-2.458
er.gnm1.ar	Others	TR	Others	ıhy.WRX7	ınner.gnm1	83	21	-1.982
ner.gnm1.a	Others	TR	Others	ahy.Y5JHF	ınner.gnm1	602	134	-2.172
ner.gnm1.a	Others	TR	Others	ahy.YRC4I	ınner.gnm1	77	23	-1.733
ner.gnm1.a	Others	TR	Others	ahy.Z2QY	ınner.gnm1	256	522	1.03
ner.gnm1.a	Others	TR	Others	ahy.ZRH2	ınner.gnm1	6	321	5.863
ner.gnm1.a	PHD	TR	PHD	rahy.0IX9E	ınner.gnm1	2	27	3.883
ner.gnm1.a	PHD	TR	PHD	ahy.14DVU	ınner.gnm1	59	5	-3.475
ner.gnm1.a	PHD	TR	PHD	ahy.1QTR	ınner.gnm1	246	37	-2.719
ner.gnm1.a	PHD	TR	PHD	ahy.8M1K	ınner.gnm1	27	3	-2.89
ner.gnm1.a	PHD	TR	PHD	ahy.EJB0X	ınner.gnm1	90	209	1.22

ner.gnm1.a	PHD	TR	PHD	rahy.J886V	inner.gnm1	66	5	-3.81
ner.gnm1.a	PHD	TR	PHD	rahy.KJ11S	inner.gnm1	128	37	-1.793
ner.gnm1.ar	PHD	TR	PHD	ahy.M25Y1	inner.gnm1	30	4	-2.905
ner.gnm1.ar	PHD	TR	PHD	ahy.M25Y1	inner.gnm1	30	4	-2.905
ner.gnm1.a	PHD	TR	PHD	rahy.S706J	inner.gnm1	30	9	-1.768
ner.gnm1.a	PHD	TR	PHD	rahy.SI6M6	inner.gnm1	110	37	-1.57
er.gnm1.ar	PHD	TR	PHD	hy.TXM5I	inner.gnm1	135	314	1.22
ner.gnm1.a	PLATZ	TF	PLATZ	rahy.C4KZ	inner.gnm1	11	1	-3.92
ner.gnm1.ar	PLATZ	TF	PLATZ	ahy.QDSR	inner.gnm1	22	1	-4.919
ner.gnm1.a	PLATZ	TF	PLATZ	ahy.RCY4	inner.gnm1	74	26	-1.529
ner.gnm1.ar	PLATZ	TF	PLATZ	ahy.YU3L	inner.gnm1	17	1	-4.05
ner.gnm1.a	eudo ARR	TR	eudo ARR	ahy.PEG0F	inner.gnm1	1926	918	-1.069
ner.gnm1.a	eudo ARR	TR	eudo ARR	ahy.V64N	inner.gnm1	2866	1351	-1.085
ner.gnm1.a	RWP-RK	TF	RWP-RK	ahy.657RU	inner.gnm1	48	177	1.868
ner.gnm1.a	RWP-RK	TF	RWP-RK	rahy.8R729	inner.gnm1	192	922	2.26
ner.gnm1.ar	RWP-RK	TF	RWP-RK	ahy.DEK8	inner.gnm1	62	214	1.779
ner.gnm1.a	RWP-RK	TF	RWP-RK	rahy.DJ079	inner.gnm1	117	48	-1.29
ner.gnm1.a	RWP-RK	TF	RWP-RK	rahy.JSL8J	inner.gnm1	405	1206	1.575
ner.gnm1.a	SBP	TF	SBP	ahy.18KTS	inner.gnm1	160	3	-5.778
ner.gnm1.ar	SBP	TF	SBP	ahy.6HMZ	inner.gnm1	165	2	-6.228
ner.gnm1.a	SBP	TF	SBP	rahy.6IJR0	inner.gnm1	288	47	-2.609
ner.gnm1.a	SBP	TF	SBP	rahy.79TY2	inner.gnm1	18	1	-4.598
ner.gnm1.ar	SBP	TF	SBP	ahy.B3PCV	inner.gnm1	16	0	-6.28
ner.gnm1.ar	SBP	TF	SBP	ahy.CR8X	inner.gnm1	296	92	-1.682
er.gnm1.ar	SBP	TF	SBP	hy.GW0D	inner.gnm1	135	0	-9.392
ner.gnm1.ar	SBP	TF	SBP	ahy.H3CM	inner.gnm1	42	13	-1.688
ner.gnm1.a	SBP	TF	SBP	rahy.I2W0I	inner.gnm1	283	86	-1.729
ner.gnm1.ar	SBP	TF	SBP	ahy.LT7EI	inner.gnm1	6	0	-4.814
ner.gnm1.a	SBP	TF	SBP	rahy.TYJ1F	inner.gnm1	129	5	-4.665
ner.gnm1.ar	SBP	TF	SBP	ahy.YYFQ	inner.gnm1	198	12	-4.062
ner.gnm1.a	SET	TR	SET	rahy.27B95	inner.gnm1	61	8	-2.927
er.gnm1.ar	SET	TR	SET	hy.BQLE9	inner.gnm1	64	25	-1.343
ner.gnm1.ar	SET	TR	SET	ahy.E1ZVI	inner.gnm1	159	79	-1.019
ner.gnm1.a	SET	TR	SET	rahy.HIV4	inner.gnm1	171	4	-5.554
ner.gnm1.ar	SET	TR	SET	ahy.RN3X	inner.gnm1	231	68	-1.763
er.gnm1.ar	SET	TR	SET	hy.UX6KI	inner.gnm1	203	76	-1.418
ner.gnm1.a	SET	TR	SET	ahy.YEC9	inner.gnm1	264	55	-2.265
ner.gnm1.ar	SET	TR	SET	ahy.ZYNV	inner.gnm1	209	64	-1.712
ner.gnm1.ar	SNF2	TR	SNF2	ahy.3X7A	inner.gnm1	529	235	-1.174
ner.gnm1.a	SNF2	TR	SNF2	rahy.5H7P	inner.gnm1	337	58	-2.533
ner.gnm1.a	SNF2	TR	SNF2	rahy.97JS8	inner.gnm1	338	154	-1.139
er.gnm1.an	SNF2	TR	SNF2	hy.BW40V	inner.gnm1	124	51	-1.297
ner.gnm1.ar	SNF2	TR	SNF2	ahy.C2GR1	inner.gnm1	348	69	-2.346
ner.gnm1.ar	SNF2	TR	SNF2	ahy.H93RV	inner.gnm1	103	16	-2.654
er.gnm1.an	SNF2	TR	SNF2	hy.WMY7	inner.gnm1	650	175	-1.897

ıer.gnm1.aı	SNF2	TR	SNF2	ahy.Y7U9ɛ	ınner.gnm1	259	560	1.11
ner.gnm1.a	SRS	TF	SRS	rahy.BI3DJ	ınner.gnm1	82	442	2.43
ıer.gnm1.aı	SRS	TF	SRS	ahy.USG1Y	ınner.gnm1	53	293	2.471
ner.gnm1.al	I/SNF-BAF	TR	I/SNF-BAF	ahy.0HJ3T	ınner.gnm1	1615	707	-1.191
ner.gnm1.al	I/SNF-BAF	TR	I/SNF-BAF	ahy.2RX12	ınner.gnm1	415	155	-1.417
ner.gnm1.al	I/SNF-BAF	TR	I/SNF-BAF	ahy.4BPU9	ınner.gnm1	802	392	-1.035
ner.gnm1.al	I/SNF-BAF	TR	I/SNF-BAF	ahy.JT5AL	ınner.gnm1	312	146	-1.099
ıer.gnm1.aı	I/SNF-BAF	TR	I/SNF-BAF	ıhy.MJPM8	ınner.gnm1	1065	445	-1.26
ner.gnm1.al	I/SNF-BAF	TR	I/SNF-BAF	ahy.MS53l	ınner.gnm1	704	296	-1.253
ıer.gnm1.aı	I/SNF-BAF	TR	I/SNF-BAF	ıhy.PC5DA	ınner.gnm1	2088	661	-1.66
ner.gnm1.a	TAZ	TR	TAZ	ahy.D773L	ınner.gnm1	203	47	-2.125
ner.gnm1.a	TAZ	TR	TAZ	ahy.S20US	ınner.gnm1	21	4	-2.253
ner.gnm1.a	TCP	TF	TCP	ahy.22FU9	ınner.gnm1	388	170	-1.186
ıer.gnm1.aı	TCP	TF	TCP	ahy.3CY5U	ınner.gnm1	41	131	1.686
ıer.gnm1.aı	TCP	TF	TCP	ahy.6V3SV	ınner.gnm1	194	93	-1.061
ner.gnm1.a	TCP	TF	TCP	ahy.7QL59	ınner.gnm1	54	10	-2.443
ıer.gnm1.aı	TCP	TF	TCP	ahy.ABQ1H	ınner.gnm1	370	86	-2.117
ıer.gnm1.aı	TCP	TF	TCP	ahy.Q9YRA	ınner.gnm1	269	121	-1.157
ner.gnm1.a	TCP	TF	TCP	ahy.T0E7S	ınner.gnm1	64	12	-2.379
ner.gnm1.a	Tify	TF	Tify	ahy.25PL5	ınner.gnm1	120	18	-2.765
ner.gnm1.a	Tify	TF	Tify	ahy.29TRS	ınner.gnm1	2327	514	-2.18
ıer.gnm1.aı	Tify	TF	Tify	ahy.A76KI	ınner.gnm1	6	60	3.191
ner.gnm1.a	Tify	TF	Tify	ahy.IG3UT	ınner.gnm1	25	130	2.408
ıer.gnm1.aı	Tify	TF	Tify	ahy.P4WX	ınner.gnm1	8	79	3.306
ner.gnm1.a	Tify	TF	Tify	ahy.TPQ86	ınner.gnm1	58	11	-2.385
ner.gnm1.a	Tify	TF	Tify	ahy.WUI64	ınner.gnm1	1665	251	-2.733
ner.gnm1.a	TRAF	TR	TRAF	ahy.4T73S	ınner.gnm1	36	236	2.729
ner.gnm1.a	TRAF	TR	TRAF	ahy.71BXT	ınner.gnm1	903	2364	1.39
ner.gnm1.a	TRAF	TR	TRAF	ahy.825X9	ınner.gnm1	62	18	-1.772
ner.gnm1.a	TRAF	TR	TRAF	ahy.96G3U	ınner.gnm1	259	103	-1.327
ıer.gnm1.aı	TRAF	TR	TRAF	ahy.A4KSI	ınner.gnm1	23	1	-4.944
ner.gnm1.a	TRAF	TR	TRAF	ahy.C5K50	ınner.gnm1	924	297	-1.639
ner.gnm1.a	TRAF	TR	TRAF	ahy.CV66A	ınner.gnm1	47	7	-2.744
ıer.gnm1.aı	TRAF	TR	TRAF	ıhy.D2XTN	ınner.gnm1	32	2	-3.644
ner.gnm1.a	TRAF	TR	TRAF	ahy.K4FBU	ınner.gnm1	75	6	-3.627
ıer.gnm1.aı	TRAF	TR	TRAF	ıhy.Q49QF	ınner.gnm1	197	399	1.02
ıer.gnm1.aı	TRAF	TR	TRAF	ıhy.R60CU	ınner.gnm1	366	1061	1.534
ner.gnm1.a	TRAF	TR	TRAF	ahy.V8ELJ	ınner.gnm1	113	50	-1.176
ıer.gnm1.aı	TRAF	TR	TRAF	ıhy.Z47KV	ınner.gnm1	61	368	2.585
ner.gnm1.a	TRAF	TR	TRAF	ahy.ZSLI3	ınner.gnm1	496	1322	1.415
ıner.gnm1.a	Trihelix	TF	Trihelix	rahy.192FJ	ınner.gnm1	416	121	-1.781
ner.gnm1.a	Trihelix	TF	Trihelix	ahy.22G7N	ınner.gnm1	390	10	-5.281
ner.gnm1.a	Trihelix	TF	Trihelix	ahy.56MB	ınner.gnm1	45	238	2.417
ner.gnm1.a	Trihelix	TF	Trihelix	ahy.6ZR46	ınner.gnm1	4253	912	-2.222
ner.gnm1.a	Trihelix	TF	Trihelix	ahy.708L2	ınner.gnm1	346	95	-1.876

ner.gnm1.ai	Trihelix	TF	Trihelix	ahy.FP0NY	inner.gnm1	918	306	-1.587
ner.gnm1.a	Trihelix	TF	Trihelix	rahy.IGA8	inner.gnm1	2340	544	-2.106
ner.gnm1.ai	Trihelix	TF	Trihelix	ahy.K6EU	inner.gnm1	1625	293	-2.471
ner.gnm1.a	Trihelix	TF	Trihelix	ahy.Q5JGI	inner.gnm1	264	1245	2.241
ner.gnm1.ar	Trihelix	TF	Trihelix	ahy.UTRD	inner.gnm1	104	260	1.323
er.gnm1.ar	Trihelix	TF	Trihelix	ahy.WDZX	inner.gnm1	1861	508	-1.875
ner.gnm1.a	Trihelix	TF	Trihelix	rahy.XCJ6	inner.gnm1	62	196	1.664
er.gnm1.an	Trihelix	TF	Trihelix	hy.XW1W	inner.gnm1	89	194	1.13
ner.gnm1.a	TUB	TF	TUB	ahy.597RP	inner.gnm1	15	2	-3.073
ner.gnm1.ai	TUB	TF	TUB	ahy.HE2FX	inner.gnm1	6	0	-4.81
ner.gnm1.a	TUB	TF	TUB	rahy.PAJJ3	inner.gnm1	8	0	-4.338
ner.gnm1.a	TUB	TF	TUB	rahy.PAJJ3	inner.gnm1	8	0	-4.338
ner.gnm1.a	ULT	TF	ULT	ahy.F3JNC	inner.gnm1	165	383	1.216
ner.gnm1.a	WRKY	TF	WRKY	ahy.091A4	inner.gnm1	536	2295	2.1
ner.gnm1.a	WRKY	TF	WRKY	ahy.0S4LA	inner.gnm1	8	51	2.655
ner.gnm1.ai	WRKY	TF	WRKY	ahy.1T4KV	inner.gnm1	11	131	3.623
ner.gnm1.ai	WRKY	TF	WRKY	ahy.34PMV	inner.gnm1	3	16	2.673
ner.gnm1.ai	WRKY	TF	WRKY	ahy.3BGC	inner.gnm1	279	684	1.291
ner.gnm1.a	WRKY	TF	WRKY	ahy.431KH	inner.gnm1	1339	233	-2.524
ner.gnm1.a	WRKY	TF	WRKY	rahy.4IR4X	inner.gnm1	71	214	1.591
ner.gnm1.a	WRKY	TF	WRKY	ahy.74VGI	inner.gnm1	12	47	2.031
ner.gnm1.ai	WRKY	TF	WRKY	ahy.77CZV	inner.gnm1	49	192	1.971
ner.gnm1.a	WRKY	TF	WRKY	rahy.9C1IJ	inner.gnm1	13	63	2.214
ner.gnm1.ai	WRKY	TF	WRKY	ahy.A3ULY	inner.gnm1	2881	559	-2.367
ner.gnm1.a	WRKY	TF	WRKY	ahy.ALL8	inner.gnm1	46	326	2.834
ner.gnm1.a	WRKY	TF	WRKY	rahy.B1IT3	inner.gnm1	1098	209	-2.393
ner.gnm1.ar	WRKY	TF	WRKY	ahy.EVAW	inner.gnm1	9	211	4.61
ner.gnm1.ai	WRKY	TF	WRKY	ahy.EXY7	inner.gnm1	3	34	3.61
er.gnm1.an	WRKY	TF	WRKY	hy.GV8AV	inner.gnm1	233	802	1.785
ner.gnm1.a	WRKY	TF	WRKY	ahy.I0TYP	inner.gnm1	2	27	3.498
ner.gnm1.a	WRKY	TF	WRKY	ahy.JRMF	inner.gnm1	424	1936	2.19
ner.gnm1.ai	WRKY	TF	WRKY	ahy.K37UI	inner.gnm1	420	82	-2.366
ner.gnm1.a	WRKY	TF	WRKY	ahy.LLK2	inner.gnm1	14	119	3.16
ner.gnm1.ai	WRKY	TF	WRKY	ahy.NA5G	inner.gnm1	46	152	1.745
ner.gnm1.a	WRKY	TF	WRKY	ahy.P7H0	inner.gnm1	3	63	4.501
ner.gnm1.ai	WRKY	TF	WRKY	ahy.P8AW	inner.gnm1	291	101	-1.52
ner.gnm1.ai	WRKY	TF	WRKY	ahy.TAM8	inner.gnm1	862	395	-1.127
ner.gnm1.a	WRKY	TF	WRKY	ahy.TC7Y	inner.gnm1	22	319	3.872
ner.gnm1.ai	WRKY	TF	WRKY	ahy.W479	inner.gnm1	2	18	3.258
er.gnm1.ar	WRKY	TF	WRKY	ahy.WH9G	inner.gnm1	3815	427	-3.161
ner.gnm1.a	WRKY	TF	WRKY	ahy.Y4PU	inner.gnm1	1077	480	-1.168
ner.gnm1.a	WRKY	TF	WRKY	ahy.YJ3JQ	inner.gnm1	295	57	-2.384
ner.gnm1.ai	WRKY	TF	WRKY	ahy.ZN7Y	inner.gnm1	729	1709	1.231

ion factors in the testa of R4 and R7 stages of Shanhua15 and Zhonghua12 peanut

pvalue	FDR	regulated	Start	End	Strand	Length	Type	GO
1.06E-06	1.59E-05	down	25700643	25707477	-	6835	otein_codi	omatin moc
0.008809	0.035595	down	2284303	2288329	-	4027	otein_codi	ethylene-ac
1.35E-09	3.99E-08	down	4028297	4032342	-	4046	otein_codi	ethylene-ac
0.002042	0.010763	up	32463332	32473037	+	9706	otein_codi	########
0.000407	0.002791	up	1473987	1474718	+	732	otein_codi	########
3.84E-07	6.4E-06	down	16755329	16758854	-	3526	otein_codi	########
0.006801	0.028862	up	7894918	7895406	+	489	otein_codi	or activity;
2.6E-05	0.000263	up	7941002	7944524	+	3523	otein_codi	########
0.008094	0.033286	down	9932157	9933686	+	1530	otein_codi	########
0.001122	0.006564	down	7805280	7807646	+	2367	otein_codi	########
8.57E-14	5.55E-12	down	1.35E+08	1.35E+08	-	1262	otein_codi	or activity;
0.005347	0.023735	down	1280507	1281121	+	615	otein_codi	########
0.007678	0.031945	up	98737122	98739745	-	2624	otein_codi	########
0.000223	0.001672	down	17225079	17227847	-	2769	otein_codi	########
0.001994	0.010567	up	23090442	23091197	-	756	otein_codi	########
0.002286	0.011815	up	40014506	40015132	+	627	otein_codi	0009873,et
3.19E-09	8.62E-08	down	1.04E+08	1.04E+08	-	1320	otein_codi	or activity;
1.06E-05	0.00012	down	8822894	8823409	+	516	otein_codi	########
2.62E-05	0.000265	up	1898822	1899271	-	450	otein_codi	iption facto
0.008031	0.033066	up	4016328	4016792	-	465	otein_codi	iption facto
4.12E-06	5.28E-05	down	3355306	3356774	+	1469	otein_codi	########
7.36E-09	1.82E-07	down	22752359	22758481	+	6123	otein_codi	--
0.000109	0.000913	up	1.17E+08	1.17E+08	+	1521	otein_codi	########
0.002188	0.011402	down	1.32E+08	1.32E+08	-	4635	otein_codi	--
1.28E-14	9.3E-13	down	29751754	29757840	-	6087	otein_codi	########
0.010055	0.03955	up	7823873	7824358	+	486	otein_codi	or activity;
0.000401	0.002756	up	8231560	8232651	+	1092	otein_codi	########
1.37E-05	0.000151	down	1.1E+08	1.1E+08	-	1248	otein_codi	873,ethyle
6.14E-07	9.79E-06	down	1.16E+08	1.16E+08	-	5440	otein_codi	########
0.009649	0.038295	down	1.52E+08	1.52E+08	+	5422	otein_codi	########
1.07E-05	0.000121	down	36394941	36397501	-	2561	otein_codi	in-activatec
0.000358	0.002501	down	14610162	14612037	-	1876	otein_codi	355,regulat
1.41E-15	1.21E-13	up	88013839	88016187	-	2349	otein_codi	355,regulat
3.74E-09	9.9E-08	down	843705	846614	-	2910	otein_codi	--
0.012223	0.046147	down	34350703	34353156	-	2454	otein_codi	in-activatec
2.83E-07	4.91E-06	up	14659380	14665897	+	6518	otein_codi	355,regulat
6.56E-08	1.32E-06	down	52772777	52775165	-	2389	otein_codi	in-activatec
7.25E-06	8.71E-05	down	1179747	1182310	+	2564	otein_codi	in-activatec
6.07E-13	3.35E-11	down	79895559	79898101	-	2543	otein_codi	in-activatec
5.59E-06	6.91E-05	down	1.32E+08	1.32E+08	+	3537	otein_codi	ding; GO:(
5.59E-06	6.91E-05	down	1.32E+08	1.32E+08	+	3537	otein_codi	ding; GO:(
3.82E-07	6.36E-06	down	88861113	88864141	+	3029	otein_codi	########
2.22E-05	0.000229	down	1.17E+08	1.17E+08	-	2391	otein_codi	355,regulat

0.000548	0.003592	up	87976295	87977546	-	1252	otein_codi	tion of tran
1.64E-06	2.34E-05	up	1611710	1614661	+	2952	otein_codi	--
2.07E-05	0.000216	down	19084887	19087046	+	2160	otein_codi	tion of tran
0.000103	0.000869	down	7948779	7950524	-	1746	otein_codi	tion of tran
0.000461	0.003098	up	94126775	94130769	-	3995	otein_codi	--
3.19E-07	5.45E-06	up	42638591	42643609	-	5019	otein_codi	--
0.000339	0.002392	down	97440973	97443938	-	2966	otein_codi	tion of tran
0.008379	0.034234	down	32002250	32004242	-	1993	otein_codi	tion of tran
0.000183	0.001411	up	1.31E+08	1.31E+08	-	5355	otein_codi	########
0.006611	0.028182	up	93201972	93211684	-	9713	otein_codi	0006355,re
5.48E-06	6.8E-05	down	1.31E+08	1.31E+08	-	2306	otein_codi	--
7.99E-07	1.23E-05	down	1.54E+08	1.54E+08	-	1717	otein_codi	n; GO:0006
2.45E-05	0.00025	up	4150790	4153634	+	2845	otein_codi	--
2.6E-07	4.53E-06	up	1666853	1669792	-	2940	otein_codi	developm
6.77E-07	1.07E-05	up	1610947	1614946	+	4000	otein_codi	way; GO:00
0.000313	0.002235	down	1.47E+08	1.47E+08	+	2561	otein_codi	way; GO:00
8.59E-05	0.000744	down	28409812	28416517	-	6706	otein_codi	########
8.89E-11	3.28E-09	down	4111347	4117372	+	6026	otein_codi	########
0.000641	0.004104	down	10728298	10735524	-	7227	otein_codi	way; GO:00
9.37E-13	4.98E-11	down	4159776	4166467	-	6692	otein_codi	########
0.000988	0.005908	down	22912943	22917489	+	4547	otein_codi	pecific DN
0.009602	0.038143	up	96759870	96766284	+	6415	otein_codi	pecific DN
1.33E-07	2.5E-06	up	3391202	3395370	+	4169	otein_codi	way; GO:00
4.26E-06	5.44E-05	down	2017414	2023492	+	6079	otein_codi	way; GO:00
0.001523	0.008459	up	6307	9576	+	3270	otein_codi	way; GO:00
5.07E-19	7.49E-17	down	25467997	25471531	+	3535	otein_codi	:0080113,r
2.32E-05	0.000238	down	19902705	19905937	+	3233	otein_codi	########
6.59E-07	1.04E-05	down	8402801	8404872	-	2072	otein_codi	,protein dir
0.002461	0.012527	down	9824380	9828012	+	3633	otein_codi	,protein dir
9.53E-11	3.48E-09	down	46375335	46378139	+	2805	otein_codi	,protein dir
5.56E-14	3.68E-12	down	1.08E+08	1.08E+08	-	3041	otein_codi	O:0006355,
0.00011	0.000915	down	1.16E+08	1.16E+08	+	4424	otein_codi	########
8.88E-10	2.73E-08	down	1.14E+08	1.14E+08	+	12090	otein_codi	c process; (
1.53E-13	9.52E-12	up	1.02E+08	1.02E+08	+	1066	otein_codi	########
3.13E-07	5.36E-06	down	1086647	1089206	-	2560	otein_codi	factor acti
0.007336	0.030762	up	1.45E+08	1.45E+08	-	5212	otein_codi	########
0.000316	0.00225	down	955171	960423	-	5253	otein_codi	3565,seque
0.009781	0.038693	down	2468242	2476478	-	8237	otein_codi	c process; (
6.83E-12	3.09E-10	down	7345694	7348877	-	3184	otein_codi	########
0.008811	0.035598	down	1.19E+08	1.19E+08	+	4498	otein_codi	########
0.004402	0.020271	down	731267	733832	+	2566	otein_codi	########
0.000618	0.003979	down	9980272	9982233	+	1962	otein_codi	########
2.6E-16	2.56E-14	down	50118700	50121735	+	3036	otein_codi	--
0.001843	0.009919	up	11043755	11045255	+	1501	otein_codi	########
1.15E-09	3.45E-08	up	1.32E+08	1.32E+08	-	3740	otein_codi	########

0.009109	0.036564	down	1.32E+08	1.32E+08	-	2607	otein_codi	########
2.77E-13	1.63E-11	down	1048779	1052077	-	3299	otein_codi	,protein dir
0.006704	0.028509	down	1.57E+08	1.57E+08	-	2717	otein_codi	--
0.000242	0.001798	down	1.34E+08	1.34E+08	-	2539	otein_codi	########
4.1E-06	5.26E-05	down	10594948	10597069	-	2122	otein_codi	,protein dir
1.18E-17	1.43E-15	down	15802995	15815996	+	13002	otein_codi	c process; (
6.94E-12	3.13E-10	down	1.33E+08	1.33E+08	-	2449	otein_codi	):0006355,
4.05E-12	1.92E-10	up	26752843	26756242	+	3400	otein_codi	,protein dir
9.21E-05	0.00079	down	37446703	37449482	+	2780	otein_codi	,protein dir
0.001011	0.00602	down	87721533	87724859	+	3327	otein_codi	########
0.000271	0.00198	down	4730198	4731619	+	1422	otein_codi	########
5.43E-08	1.11E-06	up	4288893	4292280	-	3388	otein_codi	########
0.00603	0.02616	down	13533419	13537044	+	3626	otein_codi	,protein dir
0.012626	0.047308	down	54831707	54834737	-	3031	otein_codi	########
4.27E-05	0.000406	down	1.07E+08	1.07E+08	-	4468	otein_codi	########
0.000477	0.003192	down	1.34E+08	1.34E+08	-	5769	otein_codi	########
1.29E-12	6.66E-11	down	4870759	4874187	-	3429	otein_codi	########
2.51E-07	4.4E-06	down	4295543	4298069	-	2527	otein_codi	,protein dir
3.87E-11	1.53E-09	up	25512126	25515278	-	3153	otein_codi	,protein dir
6.39E-05	0.000576	up	66166541	66169596	+	3056	otein_codi	########
2E-11	8.28E-10	up	21527344	21530900	+	3557	otein_codi	########
2.4E-07	4.23E-06	up	36740553	36743185	-	2633	otein_codi	########
0.004742	0.021534	up	1.23E+08	1.23E+08	-	2202	otein_codi	########
0.007098	0.02994	down	1.42E+08	1.42E+08	-	3419	otein_codi	,protein dir
6.91E-16	6.4E-14	down	99327035	99331216	+	4182	otein_codi	########
8.35E-05	0.000726	down	4398415	4401338	-	2924	otein_codi	,protein dir
0.005357	0.023775	up	4790190	4792892	+	2703	otein_codi	limerizatio
4.87E-09	1.26E-07	down	7061693	7064500	-	2808	otein_codi	,protein dir
2.11E-09	5.97E-08	down	17825877	17828689	+	2813	otein_codi	factor acti
1.53E-10	5.36E-09	up	1.29E+08	1.29E+08	-	4687	otein_codi	########
3.49E-09	9.31E-08	down	1.12E+08	1.12E+08	-	5025	otein_codi	########
0.002346	0.012064	down	57185713	57189750	-	4038	otein_codi	########
1.18E-07	2.26E-06	down	13568223	13570653	-	2431	otein_codi	factor acti
3.87E-09	1.02E-07	down	1.51E+08	1.51E+08	-	4935	otein_codi	########
0.003641	0.017372	down	266600	269484	-	2885	otein_codi	,protein dir
4.73E-06	5.96E-05	down	4744641	4746421	-	1781	otein_codi	factor acti
0.009568	0.03804	up	6050204	6053111	+	2908	otein_codi	,protein dir
0.000928	0.005608	down	1.3E+08	1.3E+08	-	2670	otein_codi	factor acti
0.000415	0.002837	up	22642153	22643953	+	1801	otein_codi	########
2.76E-09	7.59E-08	down	12159290	12163476	+	4187	otein_codi	########
0.000404	0.002774	down	97379079	97385073	+	5995	otein_codi	########
7.08E-05	0.00063	up	1.59E+08	1.59E+08	-	2734	otein_codi	binding tr
0.000245	0.001813	down	1.17E+08	1.17E+08	-	5069	otein_codi	uence-speci
2.52E-22	5.95E-20	up	5876309	5876683	-	375	otein_codi	########
0.002205	0.01147	up	1.1E+08	1.1E+08	+	6863	otein_codi	65,sequen

0.000106	0.000891	up	1.27E+08	1.27E+08	-	4308	otein_codi	044212,tra
1.83E-11	7.6E-10	down	22983987	22990037	+	6051	otein_codi	########
0.000247	0.001824	down	47734426	47742974	+	8549	otein_codi	########
1.31E-06	1.91E-05	up	6346989	6353680	-	6692	otein_codi	########
2.1E-05	0.000219	up	52402349	52405322	-	2974	otein_codi	########
3.02E-06	4.02E-05	down	3820645	3824182	-	3538	otein_codi	binding tr
0.003412	0.016444	up	11045167	11045571	-	405	otein_codi	########
1.23E-05	0.000138	up	23833951	23839123	+	5173	otein_codi	044212,tra
0.00515	0.023033	up	1658283	1662607	+	4325	otein_codi	########
0.006132	0.026524	up	72291302	72296528	+	5227	otein_codi	########
4.88E-18	6.33E-16	down	24326734	24331766	+	5033	otein_codi	########
1.85E-05	0.000196	up	1.22E+08	1.22E+08	-	7182	otein_codi	65,sequen
0.00095	0.005718	up	1.14E+08	1.14E+08	-	2632	otein_codi	binding tr
9.78E-06	0.000112	up	10302740	10309738	-	6999	otein_codi	GO:00435
0.002742	0.013704	down	16794729	16798308	+	3580	otein_codi	binding tr
0.000651	0.004158	down	1.43E+08	1.43E+08	-	5147	otein_codi	--
2.58E-15	2.12E-13	up	1.24E+08	1.24E+08	-	4296	otein_codi	044212,tra
6.96E-07	1.09E-05	up	1.14E+08	1.14E+08	-	768	otein_codi	########
6.33E-09	1.59E-07	down	34602480	34606092	+	3613	otein_codi	########
1.31E-06	1.92E-05	up	1132695	1133483	+	789	otein_codi	########
6.25E-06	7.63E-05	down	19494561	19498164	+	3604	otein_codi	binding tr
5.28E-07	8.55E-06	down	27396337	27400963	+	4627	otein_codi	########
4.38E-05	0.000415	down	36437808	36440469	+	2662	otein_codi	ctor activit
9.49E-20	1.56E-17	down	1.55E+08	1.55E+08	+	1777	otein_codi	ctor activit
4.68E-06	5.91E-05	down	1.18E+08	1.18E+08	-	2159	otein_codi	9,regulatio
1.1E-10	3.95E-09	down	1.14E+08	1.14E+08	-	2044	otein_codi	ctor activit
0.001432	0.008048	down	96109143	96111539	-	2397	otein_codi	9,regulatio
1.79E-20	3.3E-18	down	38660582	38663247	+	2666	otein_codi	ctor activit
4.98E-18	6.44E-16	down	81197429	81199604	+	2176	otein_codi	########
7.79E-05	0.000684	down	4383704	4384094	+	391	otein_codi	ription reg
8.59E-07	1.31E-05	down	4010800	4012960	+	2161	otein_codi	--
6.38E-06	7.77E-05	down	6355145	6355843	-	699	otein_codi	########
0.000447	0.003017	up	49590798	49594441	+	3644	otein_codi	:0006355,r
1.98E-08	4.52E-07	down	27421913	27424062	-	2150	otein_codi	--
0.000707	0.004454	down	5095546	5096241	-	696	otein_codi	########
0.009409	0.037571	up	3697429	3699062	+	1634	otein_codi	########
2.9E-05	0.000289	down	1.31E+08	1.31E+08	-	2041	otein_codi	########
0.005379	0.023854	down	2277175	2277465	+	291	otein_codi	:0006355,r
3.8E-05	0.000366	down	1.06E+08	1.06E+08	-	2356	otein_codi	########
0.001254	0.007196	up	1600992	1602950	+	1959	otein_codi	########
1.15E-28	5.79E-26	down	98770357	98772872	+	2516	otein_codi	########
5.65E-05	0.000518	down	28190625	28192560	+	1936	otein_codi	46872,meta
1.12E-06	1.67E-05	up	5221663	5223445	+	1783	otein_codi	--
9.46E-06	0.000109	up	7152193	7153638	-	1446	otein_codi	:0006355,r
6.44E-09	1.62E-07	up	1.21E+08	1.21E+08	-	1458	otein_codi	6872,metal

1.01E-12	5.32E-11	down	1.28E+08	1.28E+08	-	1928	otein_codi	--
0.00049	0.003264	down	2271177	2271467	+	291	otein_codi	:0006355,r
4.25E-05	0.000404	down	3413615	3415486	-	1872	otein_codi	########
0.00012	0.000988	up	4399729	4400262	+	534	otein_codi	:0006355,r
0.006612	0.028183	down	10491801	10493761	+	1961	otein_codi	########
0.000299	0.002149	up	1.47E+08	1.47E+08	+	3675	otein_codi	--
1.39E-09	4.12E-08	down	5609802	5611642	-	1841	otein_codi	########
2.3E-06	3.18E-05	down	1.26E+08	1.26E+08	+	1732	otein_codi	########
1.78E-07	3.25E-06	down	94867241	94868959	+	1719	otein_codi	########
0.003491	0.016764	up	1.19E+08	1.19E+08	-	3795	otein_codi	--
0.001821	0.009826	down	3033691	3036478	-	2788	otein_codi	########
7.08E-05	0.000629	down	70664814	70666699	+	1886	otein_codi	########
0.008454	0.034458	up	94416228	94420390	-	4163	otein_codi	########
2.07E-07	3.69E-06	down	40081224	40088225	-	7002	otein_codi	########
0.000329	0.002328	up	1.36E+08	1.36E+08	-	1521	otein_codi	--
2.34E-07	4.13E-06	up	1.38E+08	1.38E+08	-	1530	otein_codi	--
0.002357	0.012104	down	20861262	20864282	+	3021	otein_codi	########
2.54E-17	2.93E-15	down	17847302	17852480	-	5179	otein_codi	########
0.002064	0.010862	down	46910694	46911668	+	975	otein_codi	)3676,nucl
3.73E-16	3.62E-14	up	66363355	66365464	+	2110	otein_codi	--
2.72E-05	0.000273	up	5266770	5267672	+	903	otein_codi	,sequence-s
3.89E-06	5.03E-05	up	3510985	3511473	-	489	otein_codi	########
9.98E-16	8.83E-14	down	16098715	16101701	+	2987	otein_codi	--
1.97E-07	3.54E-06	down	14436578	14442569	-	5992	otein_codi	--
0.000939	0.005662	down	308056	312351	-	4296	otein_codi	########
0.007897	0.032643	down	6297629	6301961	-	4333	otein_codi	)3676,nucl
1.81E-05	0.000192	down	1949469	1953549	-	4081	otein_codi	ion bindin
1.78E-12	8.86E-11	down	38826102	38828102	+	2001	otein_codi	)3676,nucl
1.34E-10	4.73E-09	down	19211521	19214935	+	3415	otein_codi	--
3.03E-15	2.44E-13	down	36370291	36373374	+	3084	otein_codi	--
0.001358	0.007693	up	1.39E+08	1.39E+08	-	2749	otein_codi	):0050832,
1.74E-06	2.48E-05	up	4475182	4475634	+	453	otein_codi	########
0.000195	0.00149	up	39680722	39683697	+	2976	otein_codi	########
0.000635	0.004072	up	55242115	55244427	-	2313	otein_codi	--
0.000286	0.002069	down	1940180	1941614	-	1435	otein_codi	6872,metal
4.16E-06	5.32E-05	up	1831923	1836173	-	4251	otein_codi	--
0.000295	0.002127	up	1.36E+08	1.36E+08	-	2749	otein_codi	):0050832,
1.61E-05	0.000173	up	1.09E+08	1.09E+08	+	1290	otein_codi	ription regu
4E-08	8.51E-07	down	5628184	5629458	-	1275	otein_codi	--
0.010626	0.041372	up	28443351	28446318	+	2968	otein_codi	########
3.48E-11	1.39E-09	down	13845532	13849074	-	3543	otein_codi	########
0.004172	0.019397	up	2539040	2544526	-	5487	otein_codi	--
5.67E-07	9.13E-06	down	31890449	31897536	-	7088	otein_codi	--
0.000447	0.003021	up	1.16E+08	1.16E+08	-	765	otein_codi	########
0.000433	0.002943	down	1.53E+08	1.53E+08	+	2399	otein_codi	--

6.16E-06	7.53E-05	up	1.12E+08	1.13E+08	+	2012	otein_codi	coenzyme .
9.24E-11	3.39E-09	up	2797539	2798573	-	1035	otein_codi	,sequence-s
0.009955	0.039228	down	30492671	30496583	-	3913	otein_codi	)3676,nucl
2.37E-05	0.000242	down	3639475	3640880	-	1406	otein_codi	6872,metal
1.63E-12	8.17E-11	up	769563	770594	-	1032	otein_codi	,sequence-s
4.52E-06	5.73E-05	down	25888	30615	-	4728	otein_codi	########
1.7E-05	0.000182	up	10784823	10787530	-	2708	otein_codi	ing; GO:00
0.004146	0.019306	up	10216950	10219734	+	2785	otein_codi	ing; GO:00
0.000277	0.002014	up	44775660	44779253	+	3594	otein_codi	; GO:0016
2.48E-06	3.39E-05	down	1.11E+08	1.11E+08	-	5470	otein_codi	quence-spe
0.004441	0.020406	down	1.31E+08	1.31E+08	+	4340	otein_codi	GO:00082
2.09E-08	4.74E-07	down	6502804	6505328	+	2525	otein_codi	########
3.32E-08	7.21E-07	down	8037706	8040125	-	2420	otein_codi	########
7.65E-16	7E-14	down	98195774	98198020	-	2247	otein_codi	)4722,prote
0.011788	0.044879	down	92793864	92797880	+	4017	otein_codi	########
1.45E-05	0.000158	down	1.15E+08	1.15E+08	-	3900	otein_codi	########
1.91E-05	0.000201	down	1.08E+08	1.08E+08	-	6593	otein_codi	########
0.01235	0.0465	up	12569757	12573885	-	4129	otein_codi	ng; GO:00
8.29E-06	9.77E-05	down	8827407	8830701	-	3295	otein_codi	########
0.00134	0.007608	down	15453744	15457394	-	3651	otein_codi	########
0.002555	0.012903	down	1.43E+08	1.43E+08	-	4045	otein_codi	--
2.26E-13	1.37E-11	down	1.34E+08	1.34E+08	-	4371	otein_codi	########
0.00063	0.004046	down	9107010	9109715	-	2706	otein_codi	########
2.66E-17	3.04E-15	down	22144006	22150585	-	6580	otein_codi	ence; GO:
0.000446	0.003015	down	46499782	46503091	+	3310	otein_codi	########
5.22E-08	1.08E-06	up	45174022	45178877	+	4856	otein_codi	########
0.000599	0.003881	down	1.42E+08	1.42E+08	-	2436	otein_codi	activity; GO
2.33E-05	0.000239	down	13788950	13796185	+	7236	otein_codi	########
3.93E-06	5.06E-05	down	17739900	17742125	+	2226	otein_codi	--
3.79E-05	0.000366	down	1.21E+08	1.21E+08	+	3307	otein_codi	########
0.000955	0.005742	down	1958340	1961734	+	3395	otein_codi	activity; GO
2.06E-08	4.68E-07	down	1.17E+08	1.17E+08	+	3605	otein_codi	########
2.81E-08	6.18E-07	down	1.31E+08	1.31E+08	-	4279	otein_codi	########
1.8E-06	2.55E-05	down	23387292	23394293	-	7002	otein_codi	ence; GO:
3.71E-07	6.22E-06	down	12936061	12938928	-	2868	otein_codi	activity; GO
0.009709	0.03847	down	30468900	30475430	-	6531	otein_codi	r activity; G
1.29E-09	3.82E-08	up	1.08E+08	1.08E+08	+	4884	otein_codi	activity; GO
2.4E-15	1.99E-13	down	8958328	8961007	-	2680	otein_codi	########
4.3E-07	7.09E-06	up	62367968	62374717	-	6750	otein_codi	########
9.06E-07	1.37E-05	down	1.1E+08	1.1E+08	-	3260	otein_codi	########
1.2E-08	2.85E-07	down	61353233	61355956	-	2724	otein_codi	activity; GO
1.09E-11	4.74E-10	down	1.2E+08	1.2E+08	+	2385	otein_codi	activity; GO
0.000198	0.001513	up	27004271	27006505	+	2235	otein_codi	transferring
0.000373	0.002593	up	32533953	32536120	-	2168	otein_codi	transferring
0.007666	0.031919	up	20486710	20488909	+	2200	otein_codi	transferring

1.4E-06	2.03E-05	down	8708407	8714694	-	6288	otein_codi	--
0.003588	0.017162	down	10952304	10958588	+	6285	otein_codi	--
0.00123	0.007077	up	581013	585578	-	4566	otein_codi	########
0.009596	0.038128	up	4303653	4304186	-	534	otein_codi	--
0.000274	0.001999	down	50073906	50077062	+	3157	otein_codi	lpha-N-ace
0.000799	0.004949	down	1.01E+08	1.01E+08	-	1770	otein_codi	--
5.23E-11	2.02E-09	up	575942	579664	-	3723	otein_codi	########
2.94E-08	6.47E-07	up	21632110	21634325	+	2216	otein_codi	transferring
0.000466	0.003127	up	116257	123762	+	7506	otein_codi	--
0.00795	0.032815	down	4816404	4824408	+	8005	otein_codi	########
2.48E-09	6.91E-08	down	3263051	3265528	-	2478	otein_codi	########
6.17E-06	7.54E-05	down	7826889	7828253	-	1365	otein_codi	########
0.000262	0.001923	down	147397	149022	-	1626	otein_codi	########
0.000331	0.002337	up	12169861	12172516	+	2656	otein_codi	secondary
0.011549	0.044128	down	46790359	46794126	-	3768	otein_codi	GO:000635
3.18E-07	5.44E-06	down	1.49E+08	1.49E+08	-	3197	otein_codi	--
0.001084	0.006381	down	1.51E+08	1.51E+08	+	5450	otein_codi	GO:000635
0.001577	0.008706	down	94207648	94209012	+	1365	otein_codi	0006355,re
0.000132	0.001072	down	2029296	2033171	+	3876	otein_codi	--
0.004504	0.020639	down	94527988	94531175	+	3188	otein_codi	0006355,re
1.08E-11	4.69E-10	down	82327575	82333852	+	6278	otein_codi	--
0.000185	0.001427	down	66975934	66979061	+	3128	otein_codi	0006355,re
0.008482	0.034537	down	73424708	73427845	+	3138	otein_codi	--
0.007682	0.031953	down	1.34E+08	1.34E+08	+	2472	otein_codi	########
0.007113	0.029997	up	941244	954746	-	13503	otein_codi	ption factor
2.95E-05	0.000294	down	1.5E+08	1.5E+08	+	2650	otein_codi	########
0.011612	0.044345	up	9611305	9619259	-	7955	otein_codi	ption factor
4.45E-09	1.16E-07	down	1.35E+08	1.35E+08	-	2991	otein_codi	########
0.000897	0.005444	down	10820050	10825762	-	5713	otein_codi	########
1.74E-07	3.18E-06	down	10604528	10613994	-	9467	otein_codi	########
0.00564	0.024814	up	1.12E+08	1.12E+08	+	2533	otein_codi	########
0.000213	0.001612	down	1.33E+08	1.33E+08	-	3024	otein_codi	actor activi
0.000463	0.003111	down	3398867	3415263	-	16397	otein_coding	ng; GO:000
0.002602	0.013097	down	6708903	6712861	-	3959	otein_codi	GO:004356
1.84E-07	3.34E-06	down	9330083	9335812	-	5730	otein_codi	########
4.35E-06	5.53E-05	down	80004185	80013368	+	9184	otein_coding	ng; GO:000
0.000231	0.001726	up	23515920	23519166	+	3247	otein_codi	########
0.00051	0.003376	up	1.34E+08	1.34E+08	-	1453	otein_codi	########
0.000102	0.000858	up	1406454	1412047	+	5594	otein_coding	ng; GO:000
0.000179	0.001386	down	11222360	11226390	+	4031	otein_codi	########
1.18E-09	3.55E-08	up	28511187	28514592	+	3406	otein_codi	########
0.000411	0.002819	up	1.06E+08	1.06E+08	+	2842	otein_codi	########
0.002754	0.013751	down	1.58E+08	1.58E+08	+	6020	otein_codi	########
0.001153	0.006707	down	17934161	17944438	-	10278	otein_codi	########
2.55E-05	0.000258	down	13223949	13226054	+	2106	otein_codi	GO:004356

0.009058	0.0364	down	18180851	18185115	+	4265	otein_codi	########
1.89E-06	2.67E-05	up	1.22E+08	1.22E+08	-	6310	otein_codi	########
7.1E-25	2.37E-22	down	1.37E+08	1.37E+08	-	2287	otein_codi	########
0.006374	0.027374	up	16020676	16023743	+	3068	otein_codi	########
3.2E-05	0.000315	up	12959989	12964723	-	4735	otein_codi	g; GO:000
0.004371	0.02015	up	5926202	5931136	+	4935	otein_codi	55,regulati
0.003879	0.01826	down	1.35E+08	1.35E+08	-	7975	otein_codi	ty; GO:00
1.53E-09	4.48E-08	down	1.37E+08	1.37E+08	+	3503	otein_codi	55,regulati
1.43E-09	4.21E-08	down	25202366	25211803	+	9438	otein_codi	A binding;
5.87E-07	9.42E-06	down	1.04E+08	1.04E+08	-	4518	otein_codi	55,regulati
7.32E-21	1.4E-18	down	10386016	10388380	+	2365	otein_codi	uence-spe
0.000145	0.001156	up	62517680	62523385	+	5706	otein_codi	--
1.03E-39	1.58E-36	down	10002573	10004649	+	2077	otein_codi	uence-spe
0.000796	0.004929	up	1.03E+08	1.03E+08	-	1765	otein_codi	ment; GO:0
2.85E-09	7.81E-08	up	12198899	12200929	+	2031	otein_codi	########
0.000215	0.001622	down	38198063	38201854	+	3792	otein_codi	transcripti
2.15E-06	2.99E-05	down	39570818	39574606	+	3789	otein_codi	transcripti
1.32E-06	1.93E-05	up	89060053	89063439	-	3387	otein_codi	--
1.1E-12	5.74E-11	down	1.02E+08	1.02E+08	-	2436	otein_codi	ng; GO:000
2.3E-10	7.83E-09	down	1.53E+08	1.53E+08	-	1968	otein_codi	; transcripti
1.61E-12	8.09E-11	down	1.01E+08	1.01E+08	-	2443	otein_codi	ng; GO:000
1.43E-12	7.34E-11	down	1.15E+08	1.15E+08	+	5290	otein_codi	; transcripti
6.39E-15	4.89E-13	down	1915150	1918222	-	3073	otein_codi	tor activity
1.05E-10	3.8E-09	down	1.2E+08	1.2E+08	-	2266	otein_codi	43565,sequ
9.32E-10	2.85E-08	down	11553957	11557132	+	3176	otein_codi	43565,sequ
0.001409	0.007938	up	8725520	8728456	+	2937	otein_codi	GO:004356
9.79E-22	2.12E-19	down	16708931	16713269	+	4339	otein_codi	tor activity
0.000284	0.002061	up	1.33E+08	1.33E+08	-	2580	otein_codi	GO:004356
9.41E-07	1.42E-05	down	1.49E+08	1.49E+08	+	3836	otein_codi	GO:004356
3.7E-07	6.2E-06	down	6531489	6533729	-	2241	otein_codi	--
0.000114	0.000946	up	8528588	8531230	+	2643	otein_codi	GO:004356
0.01163	0.044391	down	1.38E+08	1.38E+08	-	4628	otein_codi	ation of tra
0.000967	0.005802	down	1.12E+08	1.12E+08	-	4597	otein_codi	ation of tra
0.000857	0.005234	down	1.39E+08	1.39E+08	+	20760	otein_codi	########
0.006168	0.026651	up	13308462	13318245	+	9784	otein_codi	005829,cy
5.04E-06	6.32E-05	down	1.15E+08	1.15E+08	+	7348	otein_codi	########
0.010616	0.041334	up	1.3E+08	1.3E+08	+	9149	otein_codi	005829,cy
6.01E-09	1.53E-07	down	88945004	88946814	-	1811	otein_codi	ent binding
0.006052	0.026243	down	1.15E+08	1.15E+08	+	2445	otein_codi	:0046872,r
1.95E-05	0.000205	up	1.06E+08	1.06E+08	-	2943	otein_codi	005634,nu
0.005789	0.025328	up	8916998	8919601	+	2604	otein_codi	,proximal/c
0.003927	0.018458	down	29281618	29282856	-	1239	otein_codi	morphogen
0.002157	0.011266	down	7122328	7124934	+	2607	otein_codi	--
0.001841	0.009914	up	9060910	9063536	+	2627	otein_codi	,proximal/c
0.000393	0.002709	down	1.31E+08	1.31E+08	-	6531	otein_codi	7,tyrosine c

0.012845	0.047975	down	1.56E+08	1.56E+08	-	1035	otein_codi	--
1.79E-05	0.000191	down	1.55E+08	1.55E+08	-	1237	otein_codi	--
0.000501	0.003323	down	204458	207567	+	3110	otein_codi	--
1.53E-07	2.84E-06	down	20212508	20213725	+	1218	otein_codi	morphogen
1.6E-05	0.000173	up	84538047	84541033	-	2987	otein_codi	005634,nu
1.18E-05	0.000133	down	32224485	32227800	-	3316	otein_codi	--
0.009685	0.038401	down	7152505	7155115	+	2611	otein_codi	--
2.84E-09	7.77E-08	up	1.09E+08	1.09E+08	-	6845	otein_codi	########
3.07E-08	6.73E-07	up	65288000	65297803	+	9804	otein_codi	########
0.000154	0.001224	up	12284902	12287618	+	2717	otein_codi	########
3.22E-15	2.58E-13	up	1.05E+08	1.05E+08	-	8991	otein_codi	########
1.36E-08	3.22E-07	up	1.12E+08	1.12E+08	-	4962	otein_codi	########
0.002417	0.01236	up	11638128	11640646	+	2519	otein_codi	--
2.99E-08	6.55E-07	up	1646001	1651342	+	5342	otein_codi	########
5.15E-06	6.45E-05	up	1799893	1808791	+	8899	otein_codi	########
2.38E-16	2.36E-14	up	1.29E+08	1.29E+08	-	9512	otein_codi	########
3.78E-07	6.31E-06	up	38848556	38857698	-	9143	otein_codi	########
0.001956	0.010404	up	1.31E+08	1.31E+08	+	801	otein_codi	########
5.5E-13	3.08E-11	down	1.38E+08	1.38E+08	+	4241	otein_codi	########
0.001757	0.009537	down	1.35E+08	1.35E+08	+	4148	otein_codi	########
0.000242	0.001799	up	1.34E+08	1.34E+08	+	5374	otein_codi	########
3.26E-05	0.00032	up	35417334	35419361	+	2028	otein_codi	tion coacti
4.5E-05	0.000425	up	13946592	13948645	+	2054	otein_codi	tion coacti
0.000541	0.003555	down	1.02E+08	1.02E+08	+	1457	otein_codi	--
0.002007	0.01062	down	2870764	2873810	+	3047	otein_codi	########
0.003752	0.017774	down	1.02E+08	1.02E+08	+	1533	otein_codi	--
0.002052	0.010807	down	1.42E+08	1.42E+08	-	7483	otein_codi	########
0.010515	0.041046	down	1.15E+08	1.15E+08	-	7927	otein_codi	########
1.33E-22	3.25E-20	down	1.15E+08	1.15E+08	-	3694	otein_codi	########
9.09E-06	0.000106	down	1.14E+08	1.14E+08	+	1647	otein_codi	908,flowe
1.7E-18	2.34E-16	down	1.2E+08	1.2E+08	-	2136	otein_codi	tion of tran
4.07E-10	1.33E-08	down	1.18E+08	1.18E+08	-	1945	otein_codi	tion of tran
0.001656	0.009077	down	41125125	41127474	+	2350	otein_codi	########
8.53E-09	2.08E-07	down	1.27E+08	1.27E+08	-	3672	otein_codi	########
0.012409	0.046682	down	80764051	80766649	-	2599	otein_codi	########
5.1E-05	0.000475	down	1.18E+08	1.18E+08	+	2670	otein_codi	########
4.82E-09	1.25E-07	down	1.42E+08	1.42E+08	-	5779	otein_codi	########
0.007678	0.031945	down	1.52E+08	1.52E+08	+	8241	otein_codi	########
3.91E-20	6.84E-18	down	93590774	93591823	-	1050	otein_codi	########
0.000169	0.001322	down	1.43E+08	1.43E+08	+	3876	otein_codi	########
0.000598	0.003877	down	1.08E+08	1.08E+08	-	1170	otein_codi	########
0.000895	0.005437	down	1.54E+08	1.54E+08	-	1047	otein_codi	908,flowe
0.000295	0.002124	down	27591815	27593754	+	1940	otein_codi	########
0.001292	0.007382	down	5815913	5818751	-	2839	otein_codi	908,flowe
1.74E-07	3.18E-06	down	12768999	12772687	-	3689	otein_codi	########

3.22E-06	4.25E-05	up	62253875	62256359	-	2485	otein_codi	tion of tran
5.74E-05	0.000525	down	1.09E+08	1.09E+08	-	5102	otein_codi	########
0.003998	0.018746	up	23094264	23095496	-	1233	otein_codi	########
0.013164	0.048906	down	1.38E+08	1.38E+08	+	1993	otein_codi	########
4.76E-08	9.93E-07	down	1.45E+08	1.45E+08	-	5814	otein_codi	########
3.67E-21	7.44E-19	down	7865058	7869082	+	4025	otein_codi	########
6E-05	0.000546	down	44870931	44872647	-	1717	otein_codi	########
8.75E-05	0.000756	up	1.01E+08	1.01E+08	-	6378	otein_codi	--
0.000703	0.004434	up	23244594	23245784	+	1191	otein_codi	########
1.38E-12	7.07E-11	down	14366426	14368800	+	2375	otein_codi	########
8.59E-35	8.27E-32	down	38100937	38102756	+	1820	otein_codi	########
0.000452	0.003051	down	5463585	5465926	+	2342	otein_codi	)908,flowe
7.68E-08	1.52E-06	down	45929006	45930796	+	1791	otein_codi	########
3.61E-05	0.000351	up	1.43E+08	1.43E+08	+	6327	otein_codi	--
1.12E-05	0.000127	down	16645741	16649216	-	3476	otein_codi	tion of tran
0.000442	0.002989	down	12625312	12628611	-	3300	otein_codi	tion of tran
8.07E-05	0.000705	up	16917107	16923273	-	6167	otein_codi	########
3.05E-36	3.32E-33	down	6615480	6618446	-	2967	otein_codi	########
6.89E-05	0.000615	up	15609871	15612810	-	2940	otein_codi	########
0.008528	0.034692	up	23278956	23280030	-	1075	otein_codi	########
2.33E-08	5.22E-07	down	1.16E+08	1.16E+08	+	3891	otein_codi	########
0.000656	0.004181	down	1.02E+08	1.02E+08	-	6434	otein_codi	########
0.000252	0.001861	down	47848801	47856094	+	7294	otein_codi	--
0.000126	0.001028	down	4808088	4813520	+	5433	otein_codi	gulation of
2.93E-24	9.18E-22	up	7287112	7288301	+	1190	otein_codi	########
8.53E-05	0.00074	up	16931722	16934390	-	2669	otein_codi	########
0.000294	0.002119	up	526667	529908	-	3242	otein_codi	--
0.002308	0.011905	down	2203454	2204614	+	1161	otein_codi	--
0.000227	0.001702	down	1.39E+08	1.39E+08	+	1259	otein_codi	)908,flowe
3.67E-08	7.89E-07	down	52861516	52863722	-	2207	otein_codi	########
2.09E-06	2.92E-05	down	67678432	67679326	-	895	otein_codi	)908,flowe
0.004944	0.022293	up	1.33E+08	1.33E+08	-	2203	otein_codi	--
0.000904	0.005482	down	95665788	95672357	+	6570	otein_codi	########
0.00224	0.01162	down	80574479	80575667	+	1189	otein_codi	########
8.02E-22	1.79E-19	down	1.09E+08	1.09E+08	+	3870	otein_codi	########
0.001002	0.005976	down	3491553	3493730	+	2178	otein_codi	########
2.88E-09	7.86E-08	down	94234410	94235777	+	1368	otein_codi	--
0.000432	0.002938	down	1.33E+08	1.33E+08	-	1249	otein_codi	########
8.51E-07	1.3E-05	down	43058362	43061924	+	3563	otein_codi	--
0.008963	0.036084	down	82913170	82914050	-	881	otein_codi	)908,flowe
1.97E-14	1.38E-12	up	13525720	13526988	-	1269	otein_codi	########
0.011815	0.044958	up	31176464	31179007	+	2544	otein_codi	########
6.85E-14	4.5E-12	down	32090209	32092077	-	1869	otein_codi	########
3.43E-10	1.13E-08	down	1450704	1453091	+	2388	otein_codi	########
1.07E-05	0.000122	down	4436627	4437994	-	1368	otein_codi	--

7.26E-05	0.000643	down	38237400	38241219	+	3820	otein_codi	--
1.21E-07	2.31E-06	down	1.49E+08	1.49E+08	-	1673	otein_codi	--
8.15E-05	0.000711	down	1.02E+08	1.02E+08	+	5397	otein_codi	--
6.37E-05	0.000575	down	43481655	43486858	+	5204	otein_codi	--
0.007996	0.032967	up	12466711	12469987	+	3277	otein_codi	--
1.65E-07	3.03E-06	down	14207391	14211790	-	4400	otein_codi	########
1.47E-62	1.01E-58	down	18197461	18202695	-	5235	otein_codi	########
0.000436	0.002961	down	16884846	16886968	-	2123	otein_codi	########
0.002975	0.014642	down	53546421	53550826	-	4406	otein_codi	########
7.07E-06	8.53E-05	up	19289698	19296599	-	6902	otein_codi	########
1.19E-05	0.000133	up	1.01E+08	1.01E+08	-	1720	otein_codi	########
0.001884	0.010094	down	18202760	18208274	+	5515	otein_codi	0090603,si
7.04E-07	1.11E-05	up	35134951	35138007	-	3057	otein_codi	ding; GO:(
0.002245	0.011639	up	99254525	99259219	-	4695	otein_codi	########
2.95E-06	3.94E-05	up	210008	211705	+	1698	otein_codi	binding tra
0.002413	0.012344	up	1.56E+08	1.56E+08	-	1917	otein_codi	########
0.002006	0.010619	up	1.26E+08	1.26E+08	-	2316	otein_codi	003700,DN
1E-09	3.04E-08	up	6544944	6546522	+	1579	otein_codi	binding tra
0.000156	0.001236	up	5793583	5797056	+	3474	otein_codi	ding; GO:(
0.005842	0.025516	up	1.24E+08	1.24E+08	+	1932	otein_codi	binding tra
2.19E-05	0.000226	up	1.32E+08	1.32E+08	-	1828	otein_codi	########
6.47E-05	0.000582	up	14868225	14874967	-	6743	otein_codi	########
0.002293	0.011844	down	11907903	11910313	+	2411	otein_codi	########
0.000258	0.001898	down	1.38E+08	1.38E+08	-	1456	otein_codi	########
4.16E-11	1.64E-09	up	40056890	40059540	-	2651	otein_codi	########
0.00529	0.023522	down	34472678	34475068	+	2391	otein_codi	########
1.09E-11	4.74E-10	down	6621922	6624293	+	2372	otein_codi	########
5.95E-17	6.41E-15	down	30826824	30829116	+	2293	otein_codi	########
1.02E-06	1.53E-05	up	1.3E+08	1.3E+08	-	1904	otein_codi	tivity; GO:
0.010249	0.040175	up	1217685	1221909	-	4225	otein_codi	########
0.001782	0.00966	down	1.31E+08	1.31E+08	+	4929	otein_codi	########
0.008648	0.035072	down	1.48E+08	1.48E+08	+	3123	otein_codi	--
0.003001	0.014747	down	1.37E+08	1.37E+08	+	4175	otein_codi	########
5.92E-05	0.000539	down	1.05E+08	1.05E+08	+	4484	otein_codi	########
3.29E-09	8.83E-08	up	51200843	51202462	-	1620	otein_codi	binding tra
0.002416	0.012354	down	29651468	29653481	-	2014	otein_codi	tion of tran
2.09E-06	2.92E-05	up	1.27E+08	1.27E+08	-	2153	otein_codi	tivity; GO:
3.75E-12	1.79E-10	up	4143171	4146189	-	3019	otein_codi	########
0.003152	0.015379	up	27133222	27134709	-	1488	otein_codi	########
2.46E-07	4.31E-06	down	1.35E+08	1.35E+08	-	1783	otein_codi	########
0.011306	0.043447	down	12184444	12184988	+	545	otein_codi	########
3.69E-06	4.81E-05	down	1.5E+08	1.5E+08	+	6316	otein_codi	########
0.012692	0.047523	up	7810468	7813061	+	2594	otein_codi	########
0.01091	0.042237	down	95117452	95123223	-	5772	otein_codi	########
0.007372	0.030894	down	1.15E+08	1.15E+08	-	2574	otein_codi	--

2.47E-05	0.000251	up	2052797	2056222	-	3426	otein_codi	########
7.37E-05	0.000652	up	26244149	26246859	-	2711	otein_codi	########
0.001004	0.005986	down	1.6E+08	1.6E+08	-	2561	otein_codi	--
5.56E-06	6.88E-05	down	13979981	13983527	+	3547	otein_codi	########
0.000402	0.002759	down	1.18E+08	1.18E+08	+	5516	otein_codi	--
0.000738	0.004617	down	1.11E+08	1.11E+08	+	4429	otein_codi	)03700,DN
9.36E-09	2.27E-07	down	44492626	44498160	-	5535	otein_codi	########
0.002538	0.012835	down	47820230	47823687	+	3458	otein_codi	)03700,DN
8.44E-14	5.49E-12	down	2248428	2249015	-	588	otein_codi	erodimeriza
3.12E-21	6.42E-19	down	937559	938146	-	588	otein_codi	########
0.000176	0.001369	up	1902649	1905985	+	3337	otein_codi	########
5.62E-15	4.36E-13	up	5734442	5736074	+	1633	otein_codi	########
0.000478	0.003199	up	7695567	7696286	-	720	otein_codi	########
0.002968	0.014617	down	7517931	7527681	+	9751	otein_codi	--
4.44E-06	5.64E-05	down	93739927	93744144	-	4218	otein_codi	########
7.25E-07	1.13E-05	down	1.36E+08	1.36E+08	-	13173	otein_codi	########
1.54E-15	1.31E-13	up	40162652	40169922	+	7271	otein_codi	--
2.09E-08	4.75E-07	down	1.27E+08	1.27E+08	+	4078	otein_codi	########
0.005654	0.024865	down	13148836	13153824	-	4989	otein_codi	ing; GO:00
6.7E-06	8.14E-05	up	15818311	15825647	-	7337	otein_codi	372,metal i
0.011904	0.045241	down	1.34E+08	1.34E+08	-	4043	otein_codi	########
9.54E-05	0.000813	down	27787037	27788611	-	1575	otein_codi	--
0.000336	0.00237	down	71136570	71140741	-	4172	otein_codi	########
6.73E-05	0.000603	down	1.42E+08	1.42E+08	+	1901	otein_codi	########
0.004088	0.019092	up	1.44E+08	1.44E+08	+	680	otein_codi	########
0.002727	0.013646	down	1.4E+08	1.4E+08	+	3638	otein_codi	########
0.001014	0.006035	up	3304701	3309586	+	4886	otein_codi	########
0.00099	0.005919	up	18620545	18622801	+	2257	otein_codi	########
0.001453	0.00815	up	1.19E+08	1.19E+08	-	10803	otein_codi	########
0.000581	0.00378	up	1.39E+08	1.39E+08	-	6957	otein_codi	########
4.11E-05	0.000392	down	5747386	5747850	-	465	otein_codi	########
0.000366	0.00255	down	1.16E+08	1.16E+08	+	2221	otein_codi	########
0.000159	0.001252	down	93799802	93803387	+	3586	otein_codi	########
0.000627	0.004026	up	9645882	9650415	+	4534	otein_codi	372,metal i
9.19E-05	0.000789	down	26048733	26052182	+	3450	otein_codi	########
0.000911	0.005517	down	9387555	9392191	-	4637	otein_codi	372,metal i
0.0002	0.001526	down	335515	336396	+	882	otein_codi	--
0.00354	0.016967	down	95497914	95501972	+	4059	otein_codi	targeting tc
0.004941	0.022284	up	928858	935835	+	6978	otein_codi	372,metal i
1.52E-17	1.8E-15	up	44695266	44702013	+	6748	otein_codi	--
0.000566	0.00369	up	73447099	73451495	-	4397	otein_codi	########
0.000504	0.003341	down	30579673	30581450	-	1778	otein_codi	########
6.53E-12	2.97E-10	down	58560425	58568125	-	7701	otein_codi	--
0.000155	0.001231	down	1.34E+08	1.34E+08	+	1878	otein_codi	########
0.000411	0.002818	up	16416553	16430913	+	14361	otein_codi	########

6.52E-09	1.63E-07	down	6180823	6189771	+	8949	otein_codi	########
1.37E-05	0.000151	down	16437662	16449973	+	12312	otein_codi	--
0.00168	0.00919	down	2529365	2535349	+	5985	otein_codi	ase activity
0.00168	0.00919	down	2529365	2535349	+	5985	otein_codi	ase activity
0.004087	0.019089	down	4795240	4803790	-	8551	otein_codi	########
0.001544	0.008555	down	21580697	21590540	+	9844	otein_codi	--
1.11E-05	0.000125	up	4730277	4737866	-	7590	otein_codi	ase activity
0.008553	0.034767	down	23011889	23020296	+	8408	otein_codi	--
0.000242	0.001797	down	17919815	17923230	+	3416	otein_codi	--
7.51E-05	0.000663	down	1.31E+08	1.31E+08	-	11323	otein_codi	--
0.001945	0.010352	down	14098924	14102352	+	3429	otein_codi	--
0.002423	0.012381	down	5211828	5217047	+	5220	otein_codi	--
0.000959	0.005763	down	6554695	6560275	+	5581	otein_codi	n of transc
5.87E-07	9.42E-06	up	2371561	2377933	-	6373	otein_codi	activity; GO
3.12E-09	8.42E-08	up	3693746	3698439	+	4694	otein_codi	binding tra
1.6E-05	0.000173	up	25739009	25745119	-	6111	otein_codi	activity; GO
0.000841	0.005157	down	4014780	4019885	+	5106	otein_codi	activity; GO
3.42E-07	5.79E-06	up	1.04E+08	1.04E+08	-	4687	otein_codi	binding tra
8.59E-16	7.74E-14	down	1.28E+08	1.28E+08	-	1756	otein_codi	########
7.22E-16	6.64E-14	down	1.31E+08	1.31E+08	-	1449	otein_codi	########
1.44E-12	7.36E-11	down	1.42E+08	1.42E+08	-	3122	otein_codi	:0006355,r
0.00184	0.009914	down	7144333	7146328	+	1996	otein_codi	########
1.51E-05	0.000164	down	34167632	34169023	+	1392	otein_codi	########
3.17E-08	6.92E-07	down	1.02E+08	1.02E+08	+	3488	otein_codi	:0006355,r
6.94E-05	0.00062	down	2260932	2262478	-	1547	otein_codi	########
0.005803	0.02538	down	2014080	2019587	-	5508	otein_codi	:0006355,r
4.5E-10	1.46E-08	down	1.45E+08	1.45E+08	-	3492	otein_codi	:0006355,r
0.009159	0.036726	down	11463762	11465084	+	1323	otein_codi	########
5.67E-12	2.64E-10	down	1.13E+08	1.13E+08	-	3695	otein_codi	developme
6.26E-12	2.87E-10	down	1.16E+08	1.16E+08	-	3700	otein_codi	developme
3.28E-05	0.000322	down	1.07E+08	1.07E+08	+	3498	otein_codi	yltransferas
0.006902	0.029226	down	1.47E+08	1.47E+08	+	6312	otein_codi	nsferase ac
0.001021	0.006064	down	1.52E+08	1.52E+08	-	6368	otein_codi	########
1.46E-18	2.01E-16	down	1.38E+08	1.38E+08	+	3717	otein_codi	isphosphat
0.00066	0.004203	down	21531845	21538172	+	6328	otein_codi	########
4.14E-06	5.3E-05	down	17618334	17621271	+	2938	otein_codi	se activity;
3.36E-06	4.42E-05	down	1.35E+08	1.35E+08	+	3418	otein_codi	isphosphat
5.48E-06	6.8E-05	down	1.27E+08	1.27E+08	+	4558	otein_codi	--
0.000456	0.003071	down	1.38E+08	1.38E+08	-	5993	otein_codi	; GO:0003
1.61E-08	3.74E-07	down	10707287	10712468	+	5182	otein_codi	########
3.37E-07	5.72E-06	down	1.06E+08	1.06E+08	-	5710	otein_codi	; GO:0003
0.007052	0.029791	down	46994644	47001340	-	6697	otein_codi	003676,nu
1.45E-06	2.1E-05	down	10265776	10270961	+	5186	otein_codi	########
0.0001	0.000846	down	42355694	42362389	+	6696	otein_codi	003676,nu
1.91E-08	4.38E-07	down	27861070	27863090	-	2021	otein_codi	ding; GO:0

0.000892	0.005422	up	22909402	22915855	+	6454	otein_codi	77,DNA bi
5.22E-06	6.52E-05	up	1.22E+08	1.22E+08	+	5823	otein_codi	########
2.86E-06	3.84E-05	up	43274078	43281143	+	7066	otein_codi	########
1.09E-05	0.000123	down	1.04E+08	1.04E+08	+	3598	otein_codi	--
3.66E-07	6.14E-06	down	15638085	15644781	-	6697	otein_codi	3677,DNA
0.000523	0.003454	down	21991467	21993337	-	1871	otein_codi	########
0.000152	0.001207	down	1.55E+08	1.55E+08	-	4342	otein_codi	3677,DNA
8.2E-09	2.01E-07	down	35335839	35337715	+	1877	otein_codi	########
6.5E-05	0.000584	down	20155349	20159943	+	4595	otein_codi	--
2.23E-12	1.09E-10	down	1.37E+08	1.37E+08	-	3606	otein_codi	--
1.92E-07	3.46E-06	down	53584503	53588907	-	4405	otein_codi	########
0.001865	0.01001	down	9873520	9886893	-	13374	otein_codi	:0008270,z
0.002133	0.011166	down	4287692	4289179	+	1488	otein_codi	########
0.009565	0.038031	up	40133651	40137664	+	4014	otein_codi	########
0.002146	0.011216	down	1.11E+08	1.11E+08	+	1317	otein_codi	--
5.87E-05	0.000535	down	28296178	28298188	-	2011	otein_codi	nent; GO:0
9.7E-06	0.000112	down	2968102	2969592	+	1491	otein_codi	########
9.17E-06	0.000107	down	1.24E+08	1.24E+08	-	1320	otein_codi	--
0.000787	0.00488	down	30175294	30177526	-	2233	otein_codi	nent; GO:0
1.26E-06	1.85E-05	down	1.06E+08	1.06E+08	+	2841	otein_codi	########
7.91E-17	8.39E-15	down	46225686	46232216	+	6531	otein_codi	########
0.001944	0.010351	up	1738701	1741196	+	2496	otein_codi	########
0.011765	0.044806	up	1.41E+08	1.41E+08	+	2085	otein_codi	########
0.006061	0.026275	up	381336	384254	+	2919	otein_codi	########
5.59E-05	0.000513	down	1.34E+08	1.34E+08	-	2898	otein_codi	########
1.26E-17	1.52E-15	down	1.28E+08	1.28E+08	+	7450	otein_codi	########
2.13E-12	1.05E-10	up	4534427	4537991	+	3565	otein_codi	043161,pro
3.48E-09	9.3E-08	up	1.11E+08	1.11E+08	+	14954	otein_codi	eed germin
0.004986	0.022454	down	2394098	2401531	+	7434	otein_codi	eed germin
8.94E-06	0.000104	down	2381282	2383293	+	2012	otein_codi	7,protein ul
7.08E-05	0.00063	down	9157463	9160745	-	3283	otein_codi	########
0.000148	0.001182	down	65214844	65219781	-	4938	otein_codi	########
0.000276	0.002007	down	1.3E+08	1.3E+08	-	2798	otein_codi	stress; GO:
1.1E-05	0.000124	down	65582832	65588937	+	6106	otein_codi	########
8.93E-10	2.74E-08	down	80705633	80711418	+	5786	otein_codi	########
0.000213	0.001611	up	4227959	4229393	-	1435	otein_codi	tion; GO:0
2.72E-10	9.16E-09	up	8006109	8010511	-	4403	otein_codi	########
0.002963	0.014596	down	1.32E+08	1.32E+08	-	2807	otein_codi	stress; GO:
7.7E-12	3.46E-10	up	7117018	7120277	-	3260	otein_codi	043161,pro
5.92E-09	1.51E-07	up	9597128	9601553	-	4426	otein_codi	########
6.32E-08	1.28E-06	down	18951483	18954427	+	2945	otein_codi	########
0.0005	0.003319	down	1.59E+08	1.59E+08	-	3520	otein_codi	binding tra
1.12E-06	1.67E-05	up	25189940	25192989	-	3050	otein_codi	########
1.66E-06	2.38E-05	down	1.5E+08	1.5E+08	-	3990	otein_codi	--
1.99E-05	0.000208	down	4078092	4080954	-	2863	otein_codi	binding tra

1.89E-08	4.35E-07	down	62047203	62051030	-	3828	otein_codi	--
2.97E-06	3.97E-05	down	1.17E+08	1.17E+08	+	2473	otein_codi	--
3.9E-21	7.87E-19	down	1.14E+08	1.14E+08	+	3415	otein_codi	--
0.00118	0.006839	up	1.2E+08	1.2E+08	-	3194	otein_codi	--
0.001435	0.008061	up	1.04E+08	1.04E+08	+	3581	otein_codi	########
3.49E-09	9.31E-08	down	1.59E+08	1.59E+08	+	3421	otein_codi	--
0.000137	0.001103	up	23717934	23721601	-	3668	otein_codi	########
0.00115	0.006692	up	89983860	89987505	+	3646	otein_codi	########
0.008798	0.035561	down	81003988	81008100	-	4113	otein_codi	iosphatidyl
0.00874	0.035381	down	111389	115501	+	4113	otein_codi	iosphatidyl
0.008414	0.034341	down	6531411	6532295	-	885	otein_codi	--
0.008414	0.034341	down	6531411	6532295	-	885	otein_codi	--
0.000309	0.002209	up	19805117	19808684	+	3568	otein_codi	########
1.17E-06	1.73E-05	up	1.49E+08	1.49E+08	+	1832	otein_codi	########
0.00064	0.004095	up	1.07E+08	1.07E+08	-	5916	otein_codi	########
1.4E-05	0.000153	up	17012671	17016947	+	4277	otein_codi	########
0.003049	0.01495	up	12660139	12662090	-	1952	otein_codi	--
7.26E-05	0.000644	up	5754659	5757697	-	3039	otein_codi	)43565,seq
1.12E-14	8.19E-13	down	25743186	25749644	+	6459	otein_codi	ing; GO:00
0.001874	0.010051	up	1.38E+08	1.38E+08	+	3791	otein_codi	########
0.000668	0.004241	up	1.06E+08	1.06E+08	-	2209	otein_codi	--
0.002991	0.014704	up	1.2E+08	1.2E+08	-	2805	otein_codi	########
0.00473	0.021494	up	1.1E+08	1.1E+08	+	1808	otein_codi	########
6.16E-13	3.4E-11	down	1.33E+08	1.33E+08	-	4252	otein_codi	########
2.14E-06	2.97E-05	up	1.49E+08	1.49E+08	+	1556	otein_codi	########
8.28E-12	3.69E-10	down	37412496	37419499	+	7004	otein_codi	ing; GO:00
5.09E-11	1.97E-09	up	1.23E+08	1.23E+08	+	2087	otein_codi	########
7.23E-05	0.000641	up	16682476	16684568	+	2093	otein_codi	):0050832,
2.09E-09	5.94E-08	up	11478720	11481948	+	3229	otein_codi	)43565,seq
0.000332	0.002348	up	1.08E+08	1.08E+08	-	2321	otein_codi	--
4.44E-06	5.63E-05	up	35583333	35587372	+	4040	otein_codi	GO:004356
3.02E-15	2.44E-13	down	2850620	2857046	+	6427	otein_codi	########
0.00109	0.006411	up	8447212	8449199	+	1988	otein_codi	GO:004356
2.78E-06	3.75E-05	up	1.04E+08	1.04E+08	-	5131	otein_codi	########
1.05E-06	1.57E-05	up	1.33E+08	1.33E+08	-	951	otein_codi	--
0.001007	0.005996	down	1.09E+08	1.09E+08	-	2554	otein_codi	GO:004356
1.27E-05	0.000142	down	11449476	11457025	-	7550	otein_codi	--
2.66E-09	7.35E-08	up	4442239	4445553	-	3315	otein_codi	########
0.012209	0.046109	up	9864670	9866514	-	1845	otein_codi	########
1.7E-12	8.5E-11	down	1.19E+08	1.19E+08	-	4247	otein_codi	########
0.000263	0.001927	down	17402091	17405101	+	3011	otein_codi	)43565,seq
3.94E-12	1.87E-10	down	1553337	1558186	-	4850	otein_codi	########
0.003832	0.018078	up	95307717	95311361	-	3645	otein_codi	########

KEGG	KOG	NR	SwissProt	TrEMBL
--	At3g4279(	bunit A-8	ifinger prot	LFIN-LIKE 3-like isoform X2 {ECO:0000313|RefSeq:]
²2-like fact	At2g2855(	ictor RAP2	tName: Ful	0313|EMBL:KRH24962.1, ECO:0000313|EnsemblPlar
²2-like fact	At2g2855(	factor RAP	tName: Ful	0313|EMBL:KRH24962.1, ECO:0000313|EnsemblPlar
Γ; AP2-like	At3g5432(	ictor WRI1	me: Full=P	aracterized protein {ECO:0000313|EMBL:OIW06152.
--	At1g7549(	t-binding p	ponsive ele	0313|EMBL:KRH61095.1, ECO:0000313|EnsemblPla
--	At3g1677(	tion factor	########	esponsive factor 2 {ECO:0000313|EMBL:ACM79120.1
ΞBP; EREI	At2g2334(	ption facto	hylene-resp	e element binding factor 2 {ECO:0000313|EMBL:AFU
²2-like fact	At4g3692(	ı APETAL	protein Al	cterized protein {ECO:0000313|EMBL:ESW28773.1};
--	At2g4034(	nt-binding	ponsive ele	e element binding factor 6 {ECO:0000313|EMBL:AFU
--	At2g4034(	t-binding p	ponsive ele	e element binding factor 6 {ECO:0000313|EMBL:AFU
ΞBP; EREI	At5g0758(	ption facto	hylene-resp	transcription factor ERF107 {ECO:0000313|RefSeq:X
ΞBP; EREI	At5g1159(	t-binding p	nsive elem	nscription factor {ECO:0000313|EMBL:CAW30732.1
--	At5g6189(	ption facto	000303|Pu	cterized protein {ECO:0000313|EMBL:ESW23712.1};
ΞBP; EREI	At3g1677(	ption facto	########	esponsive factor 2 {ECO:0000313|EMBL:ACM79120.1
ΞBP; EREI	At5g4421(	scription fa	rt=AtERF9	.1}; SubName: Full=Ethylene-responsive element-bind
--	At1g7152(	tion factor	hylene-resp	ass transcription factor {ECO:0000313|EMBL:AES939
--	At5g0758(	tion factor	hylene-resp	transcription factor ERF107 {ECO:0000313|RefSeq:X
--	At5g0541(	lement-bin	ponsive ele	0313|EMBL:ACU13469.1, ECO:0000313|EnsemblPla
--	At5g4722(	riptional ac	anscription	transcriptional activator PTI5-like {ECO:0000313|Ref:
--	At4g1750(	criptional a	anscription	transcriptional activator PTI5-like {ECO:0000313|Ref:
--	At3g2324(	tion factor	=AtERF1B	terized protein {ECO:0000313|EMBL:CCB58688.1};
Γ; AP2-like	--	--	--	sponsive transcription factor {ECO:0000313|EMBL:AF
--	At5g5008(	tion factor	hylene-resp	transcription factor {ECO:0000313|EMBL:ALA09116
--	--	--	--	omain-containing protein {ECO:0000313|EMBL:KVI0
Γ; AP2-like	At4g3775(	anscriptio	e C mutant	cterized protein {ECO:0000313|EMBL:ESW27793.1};
ΞBP; EREI	At5g6719(	tion factor	hylene-resp	e element binding factor 2 {ECO:0000313|EMBL:AFU
ΞBP; EREI	At2g4022(	ption facto	########	cterized protein {ECO:0000313|EMBL:ESR49014.1};
ΑV; RAV	At1g2556(	g transcrip	tName: Fu	0313|EMBL:KRH70603.1, ECO:0000313|EnsemblPlar
--	t1g04880_	ß protein 1	in 15; AltN	0313|EMBL:KRH36268.1, ECO:0000313|EnsemblPla
--	t1g04880_	B protein 1	in 15; AltN	0313|EMBL:KRH36268.1, ECO:0000313|EnsemblPla
ıxin-respor	At4g2908(	otein IAA2	otein 27; A	n-induced protein {ECO:0000256|RuleBase:RU00454(
ıxin-respor	At5g4370(	ein 22D [A	22D; AltN	nduced protein {ECO:0000256|RuleBase:RU004549};
ıxin-respor	At1g0424(	ein IAA4 [	22B; AltN	n-induced protein {ECO:0000256|RuleBase:RU004549
--	--	tein IAA15	--	--
ıxin-respor	At4g2908(	otein IAA2	otein 27; A	n-induced protein {ECO:0000256|RuleBase:RU00454(
--	At3g0473(	n AUX28	: Full=Aux	induced protein {ECO:0000256|RuleBase:RU004549};
--	At3g1650(	tein IAA26	ıll=Indolea	n-induced protein {ECO:0000256|RuleBase:RU004549
ıxin-respor	At4g2908(	otein IAA2	otein 27; A	n-induced protein {ECO:0000256|RuleBase:RU00454(
ıxin-respor	At4g2908(	tein IAA27	otein 27; A	n-induced protein {ECO:0000256|RuleBase:RU00454(
--	At5g6567(	otein IAA	otein IAA	nduced protein {ECO:0000256|RuleBase:RU004549};
--	At5g6567(	otein IAA	otein IAA	nduced protein {ECO:0000256|RuleBase:RU004549};
ıxin-respor	At3g2305(	tein IAA14	: Full=Ind	induced protein {ECO:0000256|RuleBase:RU004549};
ıxin-respor	At5g4370(	ein IAA4 [	22B; AltN	n-induced protein {ECO:0000256|RuleBase:RU004549

--	--	ein Os01g	3 domain-	0313|EMBL:KRH15287.1, ECO:0000313|EnsemblPla
--	At1g4948	ein At5g18	--	--
--	At5g6698	g protein	ative B3 d	domain-containing protein {ECO:0000313|EMBL:KHN
--	t3g19180_	otein At5g	=B3 domai	ng protein At5g42700-like {ECO:0000313|RefSeq:XP_
--	--	n factor A	--	ining transcription factor ABI3 {ECO:0000313|EMBL:
--	--	30905400-l	--	0313|EMBL:KRH63711.1, ECO:0000313|EnsemblPla
--	At4g0158	tion factor	=B3 domai	protein {ECO:0000313|EMBL:ESW25410.1}; Flags: F
--	--	n Os06g01	3 domain-	cterized protein {ECO:0000313|EMBL:ESW29812.1};
--	At3g2679	[Arachis h	ng transcri	ll=FUSCA3 {ECO:0000313|EMBL:AGI43901.1};
--	At2g3047	epressor V	3 domain-	aracterized protein {ECO:0000313|EMBL:BAT97615.
--	--	ein At5g18	--	--
--	At1g4948	ption facto	otein REL	0313|EMBL:KRG90691.1, ECO:0000313|EnsemblPla
--	--	YMA_11	--	0313|EMBL:KRH29567.1, ECO:0000313|EnsemblPlan
--	At2g4687	in Os03g0	g transcript	aracterized protein {ECO:0000313|EMBL:OIW04558.
--	At4g3008	r 10-like [	n response	n response factor {ECO:0000256|RuleBase:RU004561
--	At4g3008	16 isoform	n response	esponse factor {ECO:0000256|RuleBase:RU004561};
, ARF; aux	At5g6045	factor 4 [A	me: Full=A	n response factor {ECO:0000256|RuleBase:RU004561
, ARF; aux	At2g3386	factor 3 [A	response f	esponse factor {ECO:0000256|RuleBase:RU004561};
, ARF; aux	At2g4653	ctor 4-like	me: Full=A	n response factor {ECO:0000256|RuleBase:RU004561
--	At5g6045	factor 4 [A	me: Full=A	n response factor {ECO:0000256|RuleBase:RU004561
, ARF; aux	At4g2398	9 isoform	me: Full=A	n response factor {ECO:0000256|RuleBase:RU004561
, ARF; aux	At4g2398	9 isoform	me: Full=A	esponse factor {ECO:0000256|RuleBase:RU004561};
--	At2g2835	tor 10-like	n response	n response factor {ECO:0000256|RuleBase:RU004561
--	At1g3033	6 isoform	n response	esponse factor {ECO:0000256|RuleBase:RU004561};
--	At4g3008	ctor 18 [A	n response	esponse factor {ECO:0000256|RuleBase:RU004561};
ytochrome-	At1g0953	tor PIF3 [A	ort=OsbHI	0313|EMBL:KRH33665.1, ECO:0000313|EnsemblPla
ytochrome-	At1g0953	r PIF3 [A	########	0313|EMBL:KRH33665.1, ECO:0000313|EnsemblPla
--	At2g4020	bHLH51 [	rt=AtbHLH	aracterized protein {ECO:0000313|EMBL:KOM39605
--	At3g0734	bHLH78 [	rt=AtbHLH	cterized protein {ECO:0000313|EMBL:ESW06158.1}
--	At1g2249	r bHLH96	rt=AtbHLH	0313|EMBL:KRG92821.1, ECO:0000313|EnsemblPlan
--	At5g3986	r PRE6 [A	########	cterized protein {ECO:0000313|EMBL:EMJ16398.1};
--	At3g26744	ICE1-like [	########	racterized protein {ECO:0000313|EMBL:OIW15378.1
--	--	ix-loop-he	Full=Basic	aracterized protein {ECO:0000313|EMBL:KOM35016
--	At1g0953	3 isoform X	########	n factor PIF3-like {ECO:0000313|RefSeq:XP_018836
--	At1g7221	bHLH71 [	rt=AtbHLH	cterized protein {ECO:0000313|EMBL:ESW28413.1}
--	At3g26744	r ICE1 [A	########	0313|EMBL:KRH06842.1, ECO:0000313|EnsemblPla
--	At2g2277	NAI1, parti	rt=AtbHLH	cterized protein {ECO:0000313|EMBL:ESW07780.1};
--	--	protein A-	Full=Basic	p-helix protein A-like {ECO:0000313|RefSeq:XP_014
--	At4g3453	or BEE 2 [	########	0313|EMBL:KRH29448.1, ECO:0000313|EnsemblPla
--	At3g26744	CREAM2-l	########	:0000313|EMBL:KHN17463.1}; EC=1.3.1.74 {ECO:0
--	At5g6564	bHLH93 [	rt=AtbHLH	nscription factor {ECO:0000313|EMBL:ALA09121.1};
; transcripti	At1g3264	r MYC2 [A	########	n factor MYC2-like {ECO:0000313|RefSeq:XP_01450
--	--	bHLH137	--	n factor bHLH137 family {ECO:0000313|EMBL:KYP4
--	At4g2097	HLH162-li	Short=AtbH	ption factor bHLH36 {ECO:0000313|EMBL:KHN352
--	At3g5677	actor bHLH	Short=Atbl	factor bHLH106-like {ECO:0000313|RefSeq:XP_0045

--	At5g6564(	bHLH61 [.	rt=AtbHLH	ption factor bHLH93 {ECO:0000313|EMBL:KYP6638
--	At2g2276(	NAI1, parti	rt=AtbHLH	)0313|EMBL:KRH47400.1, ECO:0000313|EnsemblPla
--	--	bHLH118	--	iption factor bHLH36 {ECO:0000313|EMBL:KYP314(
--	At5g6564(	r bHLH61	rt=AtbHLH	O:0000313|EMBL:KHN04892.1}; EC=1.3.1.74 {ECO:
--	At2g4020(	r bHLH51	rt=AtbHLH	aracterized protein {ECO:0000313|EMBL:KOM39605
--	At1g6365(	A-like iso	Full=Basic	op-helix protein A-like {ECO:0000313|RefSeq:XP_014
--	At5g3986(	r PRE6 [A	########	cterized protein {ECO:0000313|EMBL:EMJ16398.1};
--	At4g3785(	r bHLH18	rt=AtbHLH	ption factor bHLH25 {ECO:0000313|EMBL:KHN475(
--	At1g2249(	HLH96-like	rt=AtbHLH	)313|EMBL:KRG92821.1, ECO:0000313|EnsemblPlan
--	At2g4228(	bHLH130	rt=AtbHLH	otion factor bHLH130 {ECO:0000313|EMBL:KHN262
; transcripti	At1g3264(	bHLH14 [.	########	)0313|EMBL:KRH06400.1, ECO:0000313|EnsemblPla
ıscription f	At3g0612(	MUTE [A	=AtbHLH	iption factor MUTE {ECO:0000313|EMBL:KYP72837
--	At3g0734(	r bHLH78	rt=AtbHLH	cterized protein {ECO:0000313|EMBL:ESW06158.1}
--	At4g3453(	I63 isoform	########	cterized protein {ECO:0000313|EMBL:AFK40379.1};
--	At4g2097(	HLH162-li	Short=AtbH	cterized protein {ECO:0000313|EMBL:ESW10085.1}
--	At3g2674	CREAM2-l	########	LA09129.1}; SubName: Full=Inducer of CBF expressi
--	At4g3453(	bHLH63 [.	########	cription factor {ECO:0000313|EMBL:AES89129.1};
--	At3g2133(	HLH87-like	t=AtbHLH	oop-helix protein BHLH11 {ECO:0000313|EMBL:ACI
--	At4g3785(	I18 isoform	rt=AtbHLH	cterized protein {ECO:0000313|EMBL:ESW27775.1};
--	At5g6564(	r bHLH93	rt=AtbHLH	0313|EMBL:KRH16909.1, ECO:0000313|EnsemblPlan
--	At2g3122(	HLH91-like	rt=AtbHLH	)313|EMBL:KRH58537.1, ECO:0000313|EnsemblPlan
--	At5g6564(	r bHLH93	rt=AtbHLH	0313|EMBL:KRH16909.1, ECO:0000313|EnsemblPlan
--	At1g7450(	or PRE3 [.	########	terized protein {ECO:0000313|EMBL:AFK36119.1};
--	At1g6881(	r bHLH30	rt=AtbHLH	aracterized protein {ECO:0000313|EMBL:ONI31297.1
--	At3g2674	tor ICE1 [.	########	CBF expression 2 {ECO:0000313|EMBL:AGP04218.1
--	At4g3785(	H25 isofor	rt=AtbHLH	iption factor bHLH25 {ECO:0000313|EMBL:KHN190
--	At3g1986(	bHLH121 [	t=AtbHLH	factor bHLH121-like {ECO:0000313|RefSeq:XP_014
--	At4g3785(	H25 isofor	rt=AtbHLH	iption factor bHLH25 {ECO:0000313|EMBL:KHN190
--	--	r bHLH96	rt=AtbHLH	0313|EMBL:KRG96859.1, ECO:0000313|EnsemblPlan
--	At3g5677(	tor bHLH	Short=Atb	factor bHLH106-like {ECO:0000313|RefSeq:XP_0045
--	At1g1012(	H74 isofor	########	cterized protein {ECO:0000313|EMBL:ESW32948.1};
--	At1g4977(	r bHLH95	########	ption factor bHLH95 {ECO:0000313|EMBL:KYP4558
, MRF4; m	At1g7221(	bHLH96 [.	rt=AtbHLH	aracterized protein {ECO:0000313|EMBL:BAT78487.
--	At1g1012(	H74 isofor	########	cterized protein {ECO:0000313|EMBL:ESW32948.1};
--	At4g3785(	bHLH18 [.	rt=AtbHLH	)0313|EMBL:KRH23624.1, ECO:0000313|EnsemblPla
--	At1g7221(	bHLH96 [.	rt=AtbHLH	iption factor bHLH96 {ECO:0000313|EMBL:KYP743(
--	At4g3785(	bHLH18 [.	rt=AtbHLH	0313|EMBL:KRH28296.1, ECO:0000313|EnsemblPlan
, MRF4; m	At1g7221(	bHLH96 [.	rt=AtbHLH	ption factor bHLH96 {ECO:0000313|EMBL:KHN273
--	At4g2097(	bHLH162 [	Short=AtbH	ption factor bHLH36 {ECO:0000313|EMBL:KHN352
--	At3g5812(	pper 34 [A	d:1190683	transcription factor {ECO:0000313|EMBL:KEH3922
--	At2g3627(	-INSENSI	=AtDPBF	SIC ACID-INSENSITIVE 5 {ECO:0000313|EMBL:KY
; transcripti	At1g4370(	tor VIP1 [.	e: Full=Tr	cterized protein {ECO:0000313|EMBL:ESW24040.1};
--	At4g3673(	ke isoform	=AtGBF1;	x-binding factor 1 {ECO:0000313|EMBL:KHM99021.
--	At5g4408(	4 isoform	########	g factor 4 {ECO:0000313|EMBL:KYP78381.1}; Flags:
; transcripti	At1g6864(	2.2 isoform	NTHIA; A	aracterized protein {ECO:0000313|EMBL:OIW16686.1

; transcripti	At5g65210	A1 isoform	nding prote	0313|EMBL:KRH43267.1, ECO:0000313|EnsemblPlan
--	At3g58120	er 61-like |	d:1190683	ine zipper 34-like {ECO:0000313|RefSeq:XP_0145243
--	t3g17610_	ike isoforn	=bZIP trans	n factor HY5-like {ECO:0000313|RefSeq:XP_004514
; transcripti	t1g08320_	A9 isoforn	########	cific DNA-binding protein TGA-2.1 {ECO:0000313|EM
--	--	ı factor 60 |	t=OsbZIP5	cription factor 60 {ECO:0000313|RefSeq:XP_0045147
--	At5g28770	ein CPRF2	RF2; AltNa	IP protein {ECO:0000313|EMBL:BAG24402.1};
--	At5g44080	4 isoform	########	g factor 4 {ECO:0000313|EMBL:KYP78381.1}; Flags:
; transcripti	At5g65210	ription fac	nding prote	X1 {ECO:0000313|RefSeq:XP_004502460.1, ECO:00
plant G-bo	At2g35530	factor 16 [	{ECO:000	0313|EMBL:KRH39597.1, ECO:0000313|EnsemblPlan
--	At2g36270	NSENSIT	=AtDPBF1	cterized protein {ECO:0000313|EMBL:ESW34886.1};
--	At3g58120	er 61-like |	d:1190683	ine zipper 34-like {ECO:0000313|RefSeq:XP_0145243
; transcripti	At1g68640	2-like isof	NTHIA; Al	racterized protein {ECO:0000313|EMBL:OIW16686.1
; transcripti	At1g43700	tor VIP1 [	e: Full=Tr	cterized protein {ECO:0000313|EMBL:ESW24040.1};
; transcripti	At3g12250	2.3 isoform	nce-specifi	terized protein {ECO:0000313|EMBL:AFK49203.1};
--	At2g40620	r RF2b [A	e: Full=Tr	0313|EMBL:KRH17183.1, ECO:0000313|EnsemblPlan
--	At4g36730	--	--	x-binding factor 1 {ECO:0000313|EMBL:KHM99021.
; transcripti	At5g65210	1 isoform	nding prote	0313|EMBL:KRH43267.1, ECO:0000313|EnsemblPlan
--	At1g75390	actor 11-lik	{ECO:000	racterized protein {ECO:0000313|EMBL:KRH05862.1
--	At3g58120	per 34 [Ar	d:1190683	transcription factor {ECO:0000313|EMBL:KEH39223
--	At1g75390	tor 11-like	{ECO:000	nding factor, putative {ECO:0000313|EMBL:EEF5262
AMP-dep	At5g28770	ein CPRF2	RF2; AltNa	IP protein {ECO:0000313|EMBL:BAG24402.1};
--	At3g58120	r 61-like [	d:1190683	ine zipper 34-like {ECO:0000313|RefSeq:XP_0144989
--	At1g25440	TANS-LIK	=Zinc fing	aracterized protein {ECO:0000313|EMBL:OIW09668.1
--	At1g25440	STANS-LI	=Zinc fing	n CONSTANS-LIKE 16 {ECO:0000313|RefSeq:XP_0
finger pro	At5g24930	STANS-L	=Zinc fing	CONSTANS-LIKE 4-like {ECO:0000313|RefSeq:XP_
--	At1g25440	TANS-LIK	=Zinc fing	n CONSTANS-LIKE 16 {ECO:0000313|RefSeq:XP_0
finger pro	At5g24930	TANS-LI	=Zinc fing	CONSTANS-LIKE 4-like {ECO:0000313|RefSeq:XP_
--	At1g25440	STANS-LI	=Zinc fing	n CONSTANS-LIKE 16 {ECO:0000313|RefSeq:XP_0
--	At5g62940	in DOF5.6	f zinc fing	r protein DOF5.6-like {ECO:0000313|RefSeq:XP_014
--	At5g60850	OF1.4 isof	DOF5.4; S	c finger protein {ECO:0000313|EMBL:OVA00370.1}
--	--	OF2.1 isof	--	r protein DOF2.1-like {ECO:0000313|RefSeq:XP_014
--	At1g51700	DOF1.7-lik	f zinc fing	c finger protein DOF1.7 {ECO:0000313|EMBL:KYP5
--	At3g61850	F3.7 isofor	cting germ	terized protein {ECO:0000313|EMBL:AFK44127.1};
--	--	F2.2 isofor	--	in DOF2.2-like isoform X1 {ECO:0000313|RefSeq:XP
--	At1g51700	DOF1.7-lik	f zinc fing	c finger protein DOF1.7 {ECO:0000313|EMBL:KYP5
--	At2g34140	ein DOF1.	ltName: F	aracterized protein {ECO:0000313|EMBL:OIV91978.1
--	At5g62940	F5.6 isofor	f zinc fing	r protein DOF5.6-like {ECO:0000313|RefSeq:XP_014
--	At3g61850	OF1.4 isof	cting germ	r protein, putative {ECO:0000313|EMBL:EEF50839.1
--	At5g62940	F5.6 isofor	f zinc fing	cterized protein {ECO:0000313|EMBL:ESW10068.1};
--	At2g34140	DOF1.5-lik	ltName: F	aracterized protein {ECO:0000313|EMBL:BAT73624.
--	--	ein DOF5.	f zinc fing	r protein DOF5.6-like {ECO:0000313|RefSeq:XP_014
--	At2g28510	F3.2 isofor	f zinc fing	r protein DOF2.1-like {ECO:0000313|RefSeq:XP_014
--	--	OF2.5 isof	--	terized protein {ECO:0000313|EMBL:AFK44127.1};
--	At3g61850	in DOF3.7	cting germ	DNA-binding family protein {ECO:0000313|EMBL:A
--	At3g61850	ein DOF3.	f zinc fing	aracterized protein {ECO:0000313|EMBL:BAT78863.

--	--	in DOF1.4	--	finger 3 protein {ECO:0000313|EMBL:ACF74278.1};
--	At3g6185(	DF1.4 isofc	cting germ	r protein, putative {ECO:0000313|EMBL:EEF50839.1]
--	At2g3759(	ein DOF2.5	f zinc fing	r protein DOF3.6-like {ECO:0000313|RefSeq:XP_014
--	At1g2831(	DF2.2 isofo	f zinc fing	factor 2 {ECO:0000313|EMBL:ATG31849.1}; Flags: F
--	At3g5087(	factor 18 [	e: Full=GA	00313|EMBL:KRH28681.1, ECO:0000313|EnsemblPlan
--	--	on factor 8	--	transcription factor {ECO:0000256|PIRNR:PIRNR016
--	At3g0674(	factor 16 [	e: Full=GA	ranscription factor family protein {ECO:0000313|EMB
--	At4g3289(	on factor 2	ne: Full=G	acterized protein {ECO:0000313|EMBL:ESW33921.1}
--	At4g3289(	n factor 2 [	ne: Full=G	acterized protein {ECO:0000313|EMBL:ESW33921.1}
--	--	n factor 8 [	--	transcription factor {ECO:0000256|PIRNR:PIRNR016
--	--	on factor 9	ne: Full=G	nscription factor {ECO:0000256|PIRNR:PIRNR01699
3; GATA-t	At4g3289(	actor 1-like	ne: Full=G	aracterized protein {ECO:0000313|EMBL:KRH33499.
--	At4g0018(	BBY 1 [A	ne: Full=A	aracterized protein {ECO:0000313|EMBL:OIW00956.
--	At1g0384(	te-domain	17785527]	aracterized protein {ECO:0000313|EMBL:BAT95063.
--	At1g2661(	OC107481	--	aracterized protein {ECO:0000313|EMBL:OIV94219.1
--	At1g2661(	LOC10763	--	cterized protein {ECO:0000313|EMBL:ESW30619.1};
--	At1g6650(	ge factor h	lation and	00313|EMBL:KRH35572.1, ECO:0000313|EnsemblPlan
--	At1g6614(	otein 4 [Ar	l=Zinc fin	0313|EMBL:KRH72945.1, ECO:0000313|EnsemblPlan
--	At1g0203(	ZAT1-like	ne: Full=Z	-finger protein, putative {ECO:0000313|EMBL:AES90
--	At1g2661(	ATPase isc	--	00313|EMBL:KRH75014.1, ECO:0000313|EnsemblPlan
--	At2g2820(	in ZAT5 [	ne: Full=Z	er protein ZFP133 {ECO:0000313|EMBL:ACA51023.1
--	At2g2871(	n ZAT12 [	T12; AltN	er protein ZAT12 {ECO:0000313|RefSeq:XP_0144974
--	At5g5463(	LOC10761	--	aracterized protein {ECO:0000313|EMBL:OIW05964.1
--	At5g6705(	LOC10762	--	uloyl esterase A {ECO:0000313|EMBL:KYP45547.1};
--	At5g6673(	-domain 1	Med:1678	ke zinc finger protein {ECO:0000313|EMBL:EOY1471
--	At1g0829(	in WIP3 [	protein 3 {	cterized protein {ECO:0000313|EMBL:ESW04416.1};
1; beta-gala	At1g5511(	e-domain 1	erminate-d	aracterized protein {ECO:0000313|EMBL:BAT98390.
--	At1g0203(	AT1-like [	ne: Full=Z	-finger protein, putative {ECO:0000313|EMBL:AES90
--	t1g47860_	OC10749	--	protein LOC106763069 {ECO:0000313|RefSeq:XP_01
--	At5g5463(	LOC10761	--	aracterized protein {ECO:0000313|EMBL:OIW05964.1
1; UBX do	At2g0165(	aining prot	ne: Full=A	X domain protein {ECO:0000313|EMBL:AET05390.1}
--	At3g5360(	ZAT12-like	ne: Full=Z	nscription factor {ECO:0000313|EMBL:AAZ03389.1}
--	At1g0384(	ACKDAW	tein 3 {EC	aracterized protein {ECO:0000313|EMBL:BAT85701.
--	At1g2661(	OC10748	--	nger protein ZAT9 {ECO:0000313|EMBL:KHN03635.
--	At3g4526(	-domain 9	PubMed:1	inc finger protein, putative {ECO:0000313|EMBL:AES
--	--	7482397 is	--	aracterized protein {ECO:0000313|EMBL:KYP38308.
1; UBX do	At2g0165(	ining prote	ne: Full=A	X domain protein {ECO:0000313|EMBL:AET05390.1}
--	At3g6058(	in ZAT9 [	ne: Full=Z	aracterized protein {ECO:0000313|EMBL:BAT97623.
--	At2g2966(	LOC1076(	--	0313|EMBL:KRG98330.1, ECO:0000313|EnsemblPlan
--	At1g0384(	ACKDAW	tein 3 {EC	0313|EMBL:KRH67672.1, ECO:0000313|EnsemblPlan
--	At1g6614(	zinc finger	l=Zinc fin	0313|EMBL:KRH72945.1, ECO:0000313|EnsemblPlan
--	7294223	_13213 [H	--	aracterized protein {ECO:0000313|EMBL:PIN14156.1
--	--	lomain 2-li	--	00313|EMBL:KRH33908.1, ECO:0000313|EnsemblPlan
--	At1g8073(	otein 1 [Ar	CO:0000	0313|EMBL:KRH61695.1, ECO:0000313|EnsemblPlan
--	At5g5201(	LOC1076(	--	racterized protein {ECO:0000313|EMBL:OMO78884.

-- At3g18030 ne decarbo C; EC=4.1 se {ECO:0000313|EMBL:KHN03161.1}; EC=4.1.1.36
-- At2g28200 in ZAT5 [/ ne: Full=Zi protein {ECO:0000313|EnsemblPlants:GLYMA11G19
-- At1g08290 in WIP3 [/ protein 3 { cterized protein {ECO:0000313|EMBL:ESW04416.1};
-- At3g45260 te-domain ' |PubMed:1 inc finger protein, putative {ECO:0000313|EMBL:AES
-- At2g28200 ZAT5-like [ne: Full=Zi protein {ECO:0000313|EnsemblPlants:GLYMA11G19
-- At1g21570 7460707 is ontaining pH domain-containing protein 7 {ECO:0000313|EMBL:]
; F-box anc At5g51980 taining pro ort=AtC3H 0313|EMBL:KRH17014.1, ECO:0000313|EnsemblPlan
; F-box anc At5g51980 g protein 4 ort=AtC3H 0313|EMBL:KRH17014.1, ECO:0000313|EnsemblPlan
in-protein] Hs8850223 akorin isof pe E3 ubiq 00313|EMBL:KRH13923.1, ECO:0000313|EnsemblPlan
LIN54; pro At4g14770 CXC 2 isof ; Short=At 0313|EMBL:KRH77830.1, ECO:0000313|EnsemblPlan
-- At1g28050 ANS-LIKE =Zinc fing aracterized protein {ECO:0000313|EMBL:KRH06867.
-- At4g38960 protein 19 }; AltName cterized protein {ECO:0000313|EMBL:ESW03746.1};
-- At4g38960 protein 19 }; AltName cterized protein {ECO:0000313|EMBL:ESW03746.1};
n phosphata At2g25620 hatase 2C 2 tein phosph in phosphatase 2C 27 {ECO:0000313|RefSeq:XP_004
; transcript At3g48160 r-like E2F AltName: F actor-like E2FE isoform X2 {ECO:0000313|RefSeq:XF
; transcript At3g48160 r-like E2F AltName: E ription factor E2F7 {ECO:0000313|EMBL:KYP38992
; transcript At3g48160 r-like E2F AltName: E ription factor E2F7 {ECO:0000313|EMBL:KYP38992
-- At4g38180 EQUENCE =Protein F/ ED SEQUENCE 5 {ECO:0000313|EMBL:KYP44692.
onent respo At1g67710 ator ARR1 R26 {ECO: nt response regulator ARR11 {ECO:0000313|EMBL:K
onent respo At1g67710 ator ARR1 R26 {ECO: nt response regulator ARR11 {ECO:0000313|EMBL:K
-- -- KAN1-lik -- aracterized protein {ECO:0000313|EMBL:BAT73614.
-- At5g16560 N1-like iso n represso essor KAN1 isoform X3 {ECO:0000313|RefSeq:XP_0
-- At2g03500 O5 isoform ######## 0313|EMBL:KRH76947.1, ECO:0000313|EnsemblPlan
-- At2g20570 K1 isoform O-RICH R esponse regulator-like APRR2 {ECO:0000313|EMBL:]
-- -- or HRS1 [######## ription factor family protein {ECO:0000313|EMBL:KF
-- At4g28610 ATION RE 1 {ECO: PHR1-LIKE 1 {ECO:0000313|RefSeq:XP_00449558
-- At1g25550 or HRS1 [omain trans t response regulator ARR2 {ECO:0000313|EMBL:KY
-- -- or KAN1 [n represso 0313|EMBL:KRH17301.1, ECO:0000313|EnsemblPla
-- -- r KAN1-li -- aracterized protein {ECO:0000313|EMBL:OAY57218
-- At1g68670 or HHO3 [scription f aracterized protein {ECO:0000313|EMBL:OIW02257.1
-- At5g45580 factor PHI L11 {ECO: cterized protein {ECO:0000313|EMBL:ESW30193.1};
onent respo At2g25180 ator ARR1 R22 {ECO: t response regulator {ECO:0000256|PIRNR:PIRNR036
-- At5g16560 N1 isoform n represso essor KAN1 isoform X3 {ECO:0000313|RefSeq:XP_0
-- At2g20570 LK1 isofor O-RICH R esponse regulator-like APRR2 {ECO:0000313|EMBL:]
-- At5g06800 r PHL5 iso IL5 {ECO: 0313|EMBL:KRG95366.1, ECO:0000313|EnsemblPla
-- At4g18020 -like APRR APRR2; A esponse regulator-like APRR2 {ECO:0000313|EMBL:
-- At2g01060 PHL7-like IL7 {ECO: transcription factor APL {ECO:0000313|EMBL:KYP3
-- At2g03500 O5 isoform ######## 0313|EMBL:KRH76947.1, ECO:0000313|EnsemblPlan
-- At4g28610 ATION RE 1 {ECO: PHR1-LIKE 1 {ECO:0000313|RefSeq:XP_00449558
-- At1g68670 r HHO3 [A scription f aracterized protein {ECO:0000313|EMBL:OIW02257.1
-- At1g69580 factor PHIL8 {ECO: protein LOC106777111 {ECO:0000313|RefSeq:XP_0
-- At1g25550 r HRS1 [A omain trans 0313|EMBL:KRG92028.1, ECO:0000313|EnsemblPlan
-- At2g30090 e HLS1 iso bable N-ac aracterized protein {ECO:0000313|EMBL:OIW16495.1
-- At2g23060 e HLS1 iso bable N-ac aracterized protein {ECO:0000313|EMBL:BAT75677.
-- At2g23060 HLS1 isof bable N-ac aracterized protein {ECO:0000313|EMBL:BAT75677.

--	t2g41450_	07645523	--	nteracting protein 1 {ECO:0000313|EMBL:KHN30400
--	t2g41450_	OC107492	--	nteracting protein 1 {ECO:0000313|EMBL:KHN30400
--	At4g37580	sferase HL	E PHOTO	cterized protein {ECO:0000313|EMBL:ESW07819.1};
--	At3g22560	OC107491	--	ECO:0000313|EMBL:KYP71767.1}; EC=2.3.1.- {ECO
--	At5g11340	07630471	me: Full=N	terized protein {ECO:0000313|EMBL:AFK33926.1};
--	At4g19980	jilG_0958	--	aracterized protein {ECO:0000313|EMBL:OIV89907.1
--	At4g37580	ferase HLS	E PHOTO	cterized protein {ECO:0000313|EMBL:ESW07819.1};
--	At2g30090	LS1-like is	bable N-ac	aracterized protein {ECO:0000313|EMBL:OIW16495.1
--	Hs766160	-like prote	--	ansferase 9-like protein {ECO:0000313|EMBL:KYP43
--	At1g07530	tein 14 [A	ort=AtSCL	-like protein 14 {ECO:0000313|RefSeq:XP_0125727
--	At1g07530	terized pro	ort=AtSCL	racterized protein {ECO:0000313|EMBL:OIW09187.1
--	At5g41920	tein 23 [A	rt=AtSCL	aracterized protein {ECO:0000313|EMBL:BAT91147.
--	At5g48150	on factor P	ily protein	cterized protein {ECO:0000313|EMBL:ESW14496.1};
--	At3g13840	way 1 pro	l=Nodulati	aling pathway 1 protein {ECO:0000313|RefSeq:XP_01
--	At3g13960	6 isoform	r 1; Short=	lating factor-like protein {ECO:0000313|EMBL:KEH
--	--	r 1 isoforn	--	0313|EMBL:KRH19280.1, ECO:0000313|EnsemblPla
--	At4g37740	g factor 1 [	r 6; Short=	aracterized protein {ECO:0000313|EMBL:OIW15560.
--	At3g13960	1 isoform	r 5; Short=	ng factor 1 isoform X1 {ECO:0000313|RefSeq:XP_01
--	--	factor 3 [A	--	0313|EMBL:KRH76368.1, ECO:0000313|EnsemblPla
--	At3g52910	g factor 3 [	r 4; Short=	cterized protein {ECO:0000313|EMBL:ESW34859.1};
--	--	factor 7 [A	--	0313|EMBL:KRG88736.1, ECO:0000313|EnsemblPla
--	At3g52910	factor 3 [A	r 4; Short=	cterized protein {ECO:0000313|EMBL:ESW34859.1};
--	--	factor 1 [A	--	aracterized protein {ECO:0000313|EMBL:KYP79142.
--	At2g22840	g factor 1 [	r 1; Short=	0313|EMBL:KRH05542.1, ECO:0000313|EnsemblPla
--	At2g23760	ain protein	ein 4; Shor	eodomain protein 2 isoform 1 {ECO:0000313|EMBL:E
--	At4g32980	1 isoform	ne: Full=H	aracterized protein {ECO:0000313|EMBL:BAT82526.
--	At2g23760	ke homeo	ein 4; Shor	terized protein {ECO:0000313|EMBL:ESR39010.1};
omeobox-l	At1g69780	ATHB-13	ne: Full=H	ine zipper protein ATHB-13 {ECO:0000313|EMBL:K
omeobox-l	At2g34710	protein AT	tein ATH	zipper protein ATHB-14 {ECO:0000313|RefSeq:XP_
omeobox-l	At3g61150	NTHOCY	-ZIP protei	SS 2-like isoform X1 {ECO:0000313|RefSeq:XP_014
omeobox-l	At3g01470	r protein H	Full=Hom	0313|EMBL:KRH54501.1, ECO:0000313|EnsemblPla
omeobox-l	At5g15150	in HAT7 is	Full=Hom	ucine zipper protein HAT7 {ECO:0000313|EMBL:KY
omeobox-l	At1g79840	rotein GL	me: Full=H	0313|EMBL:KRH09884.1, ECO:0000313|EnsemblPla
omeobox-l	At3g60390	ein HAT3	AltName: I	ne zipper protein HAT3 {ECO:0000313|RefSeq:XP_00
omeobox-l	At2g34710	protein AT	tein ATH	zipper protein ATHB-14 {ECO:0000313|RefSeq:XP_
omeobox-l	At1g79840	rotein GL	me: Full=H	ne zipper protein GLABRA 2 {ECO:0000313|EMBL:K
omeobox-l	At4g37790	protein HA	AltName: I	e zipper protein HAT22 {ECO:0000313|RefSeq:XP_0
--	At5g06710	ein HAT9	AltName: I	cine zipper protein HOX19 {ECO:0000313|EMBL:KY
--	At5g46880	ucine zipp	ll=HD-ZIP	cterized protein {ECO:0000313|EMBL:ESW30247.1};
omeobox-l	At4g40060	in ATHB-6	ne: Full=H	protein/HALZ domain-containing protein {ECO:0000
omeobox-l	At4g37790	r protein H	AltName: I	e zipper protein HAT22 {ECO:0000313|RefSeq:XP_0
omeobox-l	At3g01470	r protein H	Full=Hom	_012568041.1}; SubName: Full=homeobox-leucine zi
omeobox-l	At3g61150	IOCYANI	-ZIP protei	0313|EMBL:KRH35356.1, ECO:0000313|EnsemblPla
omeobox-l	At1g05230	r protein H	main GLA	0313|EMBL:KRH25399.1, ECO:0000313|EnsemblPla
omeobox-l	At5g06710	r protein H	AltName: I	cine zipper protein HAT14 {ECO:0000313|EMBL:KH

omeobox-l	At4g40060	in ATHB-6	ne: Full=H	protein/HALZ domain-containing protein {ECO:0000
omeobox-l	At4g32880	CVL1_050	me: Full=H	aracterized protein {ECO:0000313|EMBL:OMO96203.
omeobox-l	At1g69780	protein AT	ne: Full=H	ine zipper protein ATHB-13 {ECO:0000313|EMBL:K
omeobox-l	At1g69780	ATHB-13	ne: Full=H	0313|EMBL:KRH74822.1, ECO:0000313|EnsemblPlan
; homeobo	At1g62990	n HD1 [Ar	ne: Full=H	cterized protein {ECO:0000313|EMBL:ESW30443.1}
--	At1g62360	d-1-like LI	me: Full=H	SBH1-like isoform X1 {ECO:0000313|RefSeq:XP_0
--	At1g70510	otted-1-lik	protein kn	aracterized protein {ECO:0000313|EMBL:BAT74700.
--	At1g62360	H1 isoform	me: Full=H	transcription factor STM-like 2 {ECO:0000313|EMBL
; homeobo	At1g70510	tted-1-like	tName: Fu	terized protein {ECO:0000313|EMBL:AFK44130.1};
--	At1g62360	1 isoform	me: Full=H	transcription factor STM-like 2 {ECO:0000313|EMBL
omeobox-l	At4g36740	n ATHB-4	ne: Full=H	r protein ATHB-40 {ECO:0000313|EMBL:KYP66724.
--	--	HAT14-li	--	--
omeobox-l	At4g36740	rotein ATH	ne: Full=H	DX transcription factor {ECO:0000313|EMBL:ALA09
--	At4g35550	omeobox 8	eobox 8; A	L:AKN10292.1}; SubName: Full=Wuschel-related hom
--	At3g11260	omeobox 5	Full=WUS	L-related homeobox 5 {ECO:0000313|EMBL:KHN13
--	--	homeobox	-related h	related homeobox 6 {ECO:0000313|RefSeq:XP_0145
--	--	omeobox 1	-related h	related homeobox 6 {ECO:0000313|RefSeq:XP_0145
--	--	11-like is	--	aracterized protein {ECO:0000313|EMBL:KCW89937
eam-bindi	At4g11080	B protein	in 13; Alt	aracterized protein {ECO:0000313|EMBL:BAT72999.
high mobil	At5g23420	B protein	rt=Nucleos	B protein 7-like isoform X2 {ECO:0000313|RefSeq:X
eam-bindi	At4g11080	ein 6 isofo	in 13; Alt	000313|EMBL:KRH41296.1, ECO:0000313|Ensembl
--	--	isoform X	rt=Nucleos	B protein 7-like isoform X2 {ECO:0000313|RefSeq:X
k transcript	At3g22830	factor A-	iption fact	0313|EMBL:KRG95588.1, ECO:0000313|EnsemblPlan
k transcript	At1g46264	on factor B	ription fac	cription factor B-4-like {ECO:0000313|RefSeq:XP_01
k transcript	At2g41690	ess transcri	ription fac	0313|EMBL:KRH66906.1, ECO:0000313|EnsemblPlan
k transcript	At4g36990	otein HSF2	at shock tr	A87077.1}; SubName: Full=Heat shock transcription f
k transcript	At3g22830	factor A-	iption fact	0313|EMBL:KRG95588.1, ECO:0000313|EnsemblPlan
k transcript	At2g26150	tor A-2 iso	at shock tr	e {ECO:0000313|RefSeq:XP_014500015.1, ECO:0000
--	At2g26150	tor A-2 iso	at shock tr	transcription factor A-2 {ECO:0000313|EMBL:KHN0
k transcript	--	--	--	cription factor B-4-like {ECO:0000313|RefSeq:XP_01
k transcript	At4g36990	ein HSF24	at shock tr	A87077.1}; SubName: Full=Heat shock transcription f
--	At5g05140	anscription	mediator of	aracterized protein {ECO:0000313|EMBL:OIW03214.
--	At5g05140	nscription	nediator of	aracterized protein {ECO:0000313|EMBL:OIW03214.
pecific den	At3g07610	07605295	########	ecific demethylase 3B {ECO:0000313|EMBL:KYP331
--	At1g78280	0 isoform	ne: Full=F-	0313|EMBL:KRH53030.1, ECO:0000313|EnsemblPla
pecific den	At3g07610	JMJ25 isof	########	cterized protein {ECO:0000313|EMBL:ESW14774.1}
--	At1g78280	80 isoform	ne: Full=F-	0313|EMBL:KRH53030.1, ECO:0000313|EnsemblPla
steine and	At1g10200	rotein WL	AltName:	0313|EMBL:KRH03352.1, ECO:0000313|EnsemblPla
--	At3g55770	otein WLIM	{ECO:000	aracterized protein {ECO:0000313|EMBL:OIV89020.1
--	At2g40470	ing protein	Name: Full	313|EMBL:KRH64169.1, ECO:0000313|EnsemblPlan
--	At5g66870	protein 36	tName: Fu	aracterized protein {ECO:0000313|EMBL:BAU00182.
--	At2g30130	protein 12-l	tName: Fu	cterized protein {ECO:0000313|EMBL:EMJ19714.1};
--	At4g37540	ing protein	Name: Full	313|EMBL:KRH77108.1, ECO:0000313|EnsemblPlan
--	At5g66870	protein 36	tName: Fu	aracterized protein {ECO:0000313|EMBL:BAU00182.
--	At2g20340	1 isoform	ll=Tyrosin	ne decarboxylase 1 {ECO:0000313|EMBL:KYP62403.

--	At3g49940	otein 38 isc	Name: Full	0313|EMBL:KRG91097.1, ECO:0000313|EnsemblPlar
--	At1g68510	g protein 4(	Name: Full	in-containing protein 42 {ECO:0000313|EMBL:KYP4
--	--	in 36-like	--	aracterized protein {ECO:0000313|EMBL:BAT75558.
--	At2g30130	protein 12-lt	Name: Fu	cterized protein {ECO:0000313|EMBL:EMJ19714.1};
--	At2g40470	g protein 1	Name: Full	0313|EMBL:KRH64169.1, ECO:0000313|EnsemblPlan
--	At1g31320	ing protein	Name: Full	ain-containing protein 4 {ECO:0000313|EMBL:KYP43
--	At4g37540	g protein 3	Name: Full	0313|EMBL:KRH77108.1, ECO:0000313|EnsemblPlan
DS-box tra	At1g69120	LIFLOWE	me: Full=	box protein {ECO:0000313|EMBL:AFI08227.1};
DS-box tra	At4g37940	tor 27 isof	factor 27;	0313|EMBL:KRH16882.1, ECO:0000313|EnsemblPla
DS-box tra	At3g54340	DS 1 isof	ull=Floral	scription factor TM6 {ECO:0000313|EMBL:AEW4360
DS-box tra	At1g69120	CAULIFLO	ADS-box p	aracterized protein {ECO:0000313|EMBL:KJB07014.
DS-box tra	At3g54340	n DEFICIE	ull=Floral	0313|EMBL:KRH51760.1, ECO:0000313|EnsemblPlar
--	At3g54340	DS 1 isof	--	scription factor TM6 {ECO:0000313|EMBL:AEW4360
DS-box tra	At3g54340	DEFICIEN	ull=Floral	0313|EMBL:KRH51760.1, ECO:0000313|EnsemblPlar
DS-box tra	At2g42830	protein A	-box prote	aracterized protein {ECO:0000313|EMBL:BAT88969.
DS-box tra	At1g69120	AULIFLO	ADS-box p	aracterized protein {ECO:0000313|EMBL:KJB07014.
DS-box tra	At4g37940	tor 27 isof	factor 27;	0313|EMBL:KRH16882.1, ECO:0000313|EnsemblPla
-box transc	At5g48670	protein AC	=Agamous	cterized protein {ECO:0000313|EMBL:ESW35360.1};
--	At1g18750	n AGL30	ius-like MA	x transcription factor 1 {ECO:0000313|EMBL:KYP76
--	At1g18750	n AGL30	ius-like MA	x transcription factor 1 {ECO:0000313|EMBL:KYP76
--	At5g48670	protein AC	=Agamous	cterized protein {ECO:0000313|EMBL:ESW35360.1};
putative tr	At2g42680	ng factor 1	: Full=Mu	ein-bridging factor 1b {ECO:0000313|EMBL:KYP450
putative tr	At2g42680	ng factor 1	: Full=Mu	ein-bridging factor 1b {ECO:0000313|EMBL:KYP450
--	At5g07900	LOC10763	--	TERF protein {ECO:0000313|EMBL:KEH32308.1};
lomain-cor	At5g55580	MTERF9,	########	n LOC106765290 isoform X2 {ECO:0000313|RefSeq:
--	--	--	--	TERF protein {ECO:0000313|EMBL:KEH32308.1};
lomain-cor	At4g14600	RF5, chlor	########	ed protein {ECO:0000313|EMBL:KYP40824.1}; Flags
lomain-cor	At4g14600	MTERF5, c	########	ed protein {ECO:0000313|EMBL:KYP40824.1}; Flags
--	At3g61250	tein 308 [A	00303|Pub	cterized protein {ECO:0000313|EMBL:ESW11886.1};
--	At5g04760	VARICAT	ll=Transcr	duced protein 1-3-1B {ECO:0000313|EMBL:BAB8689
anscription	At2g16720	YB6 isofo	ame: Full=	cterized protein {ECO:0000313|EMBL:ESW09812.1};
anscription	At4g38620	or MYB6	ame: Full=	0313|EMBL:KRH54016.1, ECO:0000313|EnsemblPlar
anscription	At3g12720	38 isoform	Myb homo	iption factor MYB86 {ECO:0000313|EMBL:KYP5907
--	At3g61250	tein 308 [A	00303|Pub	cterized protein {ECO:0000313|EMBL:ESW11886.1};
anscription	At2g37630	or AS1 [Ar	########	cription factor AS1 {ECO:0000313|EMBL:KHN48368
anscription	--	B56 isoform	CSA {E	factor MYB44-like protein {ECO:0000313|EMBL:KH
B; transcr	At5g11510	MYB3R-1	3R-4 {EC	elated protein 3R-1 {ECO:0000313|EMBL:KHN47505
--	At4g18770	MYB118-li	########	factor MYB108-like {ECO:0000313|RefSeq:XP_0145
anscription	At3g13540	MYB3-like	ltName: F	aracterized protein {ECO:0000313|EMBL:KCW89883
--	At5g08520	or SRM1 [	rotein SRM	aracterized protein {ECO:0000313|EMBL:KOM46236.
--	At5g08520	MYBS1 [	rotein SRM	B-related protein 28 {ECO:0000313|EMBL:AHB5961
--	At5g04760	VARICAT	ll=Transcr	b-like protein J {ECO:0000313|EMBL:KYP69140.1};
--	At3g46130	B48 isofor	MYB48; A	0313|EMBL:KRH10298.1, ECO:0000313|EnsemblPlar
--	At2g38090	IVARICA	ll=Transcr	B-related protein 30 {ECO:0000313|EMBL:AHB5961
anscription	At5g49330	r MYB12	########	related protein {ECO:0000313|EMBL:BAF74782.1};

anscriptior	At1g66230	NT1-like [	ODORANT1	in ODORANT1 {ECO:0000313|EMBL:KYP67201.1}
anscriptior	At1g66390	IYB90-like	elated prot	n factor MYB113 family {ECO:0000313|EMBL:KYP4
anscriptior	At3g13540	MYB6-lik	AltName: F	aracterized protein {ECO:0000313|EMBL:BAT87828.
anscriptior	At3g01140	n 306-like	Med:11597	cterized protein {ECO:0000313|EMBL:ESW30636.1};
'B; transcr	At5g11510	MYB3R-1	n 3R-4 {EC	0313|EMBL:KRH52681.1, ECO:0000313|EnsemblPlan
anscriptior	--	--	########	related protein {ECO:0000313|EMBL:BAF74782.1};
anscriptior	At1g22640	MYB8-lik	MYB3; A	tion repressor MYB4 {ECO:0000313|EMBL:KYP4257
anscriptior	--	7490672 is	--	aracterized protein {ECO:0000313|EMBL:BAU02711.
anscriptior	At2g47190	MYB108-li	CO:000030	factor MYB108-like {ECO:0000313|RefSeq:XP_0044
anscriptior	At4g01680	ssor MYB6	Full=Myb	0313|EMBL:KRH68350.1, ECO:0000313|EnsemblPlan
anscriptior	At3g13540	1 protein-l	AltName: F	protein {ECO:0000313|EnsemblPlants:GLYMA05G04
--	At5g58900	VARICAT	ull=Transcr	B-related protein 30 {ECO:0000313|EMBL:AHB5961
anscriptior	At3g13540	r MYB5-lik	AltName: F	factor MYB114-like {ECO:0000313|RefSeq:XP_0045
anscriptior	At3g18100	07644225	--	n LOC101490682 isoform X1 {ECO:0000313|RefSeq:
anscriptior	At2g16720	like isoforr	Full=Antho	aracterized protein {ECO:0000313|EMBL:BAT98311.
anscriptior	At2g16720	like isoforr	Full=Antho	n factor WER-like {ECO:0000313|RefSeq:XP_014515
anscriptior	At2g02820	124 isoforn	: Full=Myb	-related protein B {ECO:0000313|EMBL:KYP65850.1
anscriptior	At4g01680	MYB61 [	Full=Myb	0313|EMBL:KRH68350.1, ECO:0000313|EnsemblPlan
'B; transcr	At5g02320	3R-3 isofo	Med:1159	R MYB protein 10 {ECO:0000313|EMBL:AHB59598.1
--	At1g48000	78-like isof	Full=Myb-	nscription factor {ECO:0000313|EMBL:AES61608.1};
--	At5g08520	r SRM1 [A	rotein SRM	aracterized protein {ECO:0000313|EMBL:KOM46236.
'B; transcr	At4g32730	MYB3R-1 [	########	0313|EMBL:KRH52681.1, ECO:0000313|EnsemblPlan
--	--	y member 1	--	aracterized protein {ECO:0000313|EMBL:OIW04845.
--	At1g07540	ing protein	at-binding	0313|EMBL:KRH29780.1, ECO:0000313|EnsemblPla
anscriptior	At5g40350	YB108-lik	Full=Myb-	tor MYB21 {ECO:0000313|EMBL:KHN14149.1}; Fla
anscriptior	At1g66230	ANT1 [Ar	b-related p	aracterized protein {ECO:0000313|EMBL:BAT81263.
--	--	LOC10761	--	protein LOC101488688 {ECO:0000313|RefSeq:XP_00
--	--	MYB6-lik	--	--
--	At2g21650	DIALIS-lil	Full=Trans	cterized protein {ECO:0000313|EMBL:ESW20833.1}
--	At3g16350	MYBS3 [	Full=Myb-	protein LOC106762661 {ECO:0000313|RefSeq:XP_01
--	At4g39250	S-like 3 [A	Full=Trans	B-related protein 26 {ECO:0000313|EMBL:AHB5961
--	--	LOC10761	--	aracterized protein {ECO:0000313|EMBL:KDO32728.
anscriptior	At3g09370	MYB3R-5	Med:1159	tor MYB65 {ECO:0000313|EMBL:ABH02910.1}; Fla
--	At3g09370	MYB3R-5	Med:1159	lated protein B {ECO:0000313|RefSeq:XP_012568996
anscriptior	At5g15310	MYB34 [	########	on factor MYB34 {ECO:0000313|RefSeq:XP_014518
--	At5g47390	MYBS3 [	Full=Myb-	yb-like protein J {ECO:0000313|EMBL:KYP54264.1};
anscriptior	--	TT2-like [	--	2R3 MYB protein 8 {ECO:0000313|EMBL:AHB59596
--	At5g56840	r MYBS3	Full=Myb-	ption factor MYB1R1 {ECO:0000313|EMBL:KHN322
--	--	ding factor	--	--
--	At4g39250	S-like 3 [A	Full=Trans	B-related protein 26 {ECO:0000313|EMBL:AHB5961
anscriptior	At2g47190	MYB108-li	CO:000030	aracterized protein {ECO:0000313|EMBL:BAT86226.
--	At1g70000	MYB1R1	########	B-related protein 16 {ECO:0000313|EMBL:AHB5960
--	At2g46410	ETC3 iso	tion factor	n and cpc, putative {ECO:0000313|EMBL:EEF32645.1
--	At3g16350	r MYBS3	Full=Myb-	ction factor family protein {ECO:0000313|EMBL:AES
anscriptior	--	B59 isofor	--	cterized protein {ECO:0000313|EMBL:ACJ85039.1};

--	--	--	--	--
anscription	--	-like isofor	--	2R3 MYB protein 8 {ECO:0000313|EMBL:AHB59596
--	--	LOC10761	--	cterized protein {ECO:0000313|EMBL:ESW21075.1};
--	--	OC107458	--	protein LOC101504548 {ECO:0000313|RefSeq:XP_0
--	--	OC107478	--	protein LOC101488688 {ECO:0000313|RefSeq:XP_0
--	At2g46410	d protein 2	tion factor	B-related protein 25 {ECO:0000313|EMBL:AHB5961
--	At2g46410	d protein 2	tion factor	B-related protein 25 {ECO:0000313|EMBL:AHB5961
anscription	At2g46410	factor ET	tion factor	ription factor TRY {ECO:0000313|EMBL:KYP67378.
--	At1g12260	otein 7 iso	########	cterized protein {ECO:0000313|EMBL:ESW27620.1};
--	At1g56010	COTYLED	n-containi	protein {ECO:0000313|EnsemblPlants:GLYMA13G1
--	At5g13180	ing protein	AC083 {EC	ntaining protein 83-like {ECO:0000313|RefSeq:XP_01
--	At1g65910	g protein 8	M-like prot	0313|EMBL:KRH17271.1, ECO:0000313|EnsemblPla
--	At4g17980	g protein 4	:0000303|	in-containing protein 78 {ECO:0000313|EMBL:KYP5
--	At4g35580	-like 9 [Ar	8162}; Alt	LOC101492664 isoform X2 {ECO:0000313|RefSeq:
--	At5g61430	ng protein	3|PubMed	0313|EMBL:KRH56898.1, ECO:0000313|EnsemblPla
--	At2g27300	g protein 4	=ANAC04	aracterized protein {ECO:0000313|EMBL:BAT92104.
--	At5g18270	ng protein	:0000303|	ntaining protein 100-like {ECO:0000313|RefSeq:XP_0
--	At5g22380	g protein 9	domain-c	in-containing protein 90 {ECO:0000313|EMBL:KYP4
--	At4g17980	g protein 8	:0000303|	in-containing protein 78 {ECO:0000313|EMBL:KYP5
--	At5g61430	g protein 9	3|PubMed	0313|EMBL:KRH40060.1, ECO:0000313|EnsemblPlan
--	At5g13180	ing protein	AC083 {EC	ntaining protein 83-like {ECO:0000313|RefSeq:XP_01
--	At1g56010	OTYLED	n-containi	protein {ECO:0000313|EnsemblPlants:GLYMA13G1
--	At4g28500	ing protein	|PubMed:1	ntaining protein 73-like {ECO:0000313|RefSeq:XP_0
--	At1g69490	factor 29	AC029; Alt	ACO40486.1}; SubName: Full=NAC transcription fac
--	At5g53950	OTYLEDC	domain-c	rotein, IPR003441, putative {ECO:0000313|EMBL:EO
--	At4g28500	g protein 7	|PubMed:1	ntaining protein 73-like {ECO:0000313|RefSeq:XP_0
--	At2g27300	otein 40 is	=ANAC04	aracterized protein {ECO:0000313|EMBL:BAT92104.
--	At2g27300	otein 40 is	=ANAC04	aracterized protein {ECO:0000313|EMBL:BAT92104.
--	--	actor 25-lik	AC transc	haracterized protein {ECO:0000313|EMBL:PIA40769.
--	At1g56010	TYLEDON	n-containi	ED COTYLEDON 3-like {ECO:0000313|RefSeq:XP_
--	At1g28470	g protein 1	|PubMed:1	ain-containing protein 8 {ECO:0000313|EMBL:KHN0
--	--	ing protein	--	0313|EMBL:KRH34977.1, ECO:0000313|EnsemblPla
--	At3g01600	MA RESP	########	-containing protein 8 {ECO:0000313|RefSeq:XP_0044
--	At3g01600	SPONSE 1	########	aracterized protein {ECO:0000313|EMBL:OIW03013.
--	At5g61430	g protein 1	3|PubMed	0313|EMBL:KRH56898.1, ECO:0000313|EnsemblPla
--	At4g27410	otein 72 is	Short=AN	transcription factor {ECO:0000313|EMBL:ACI42832.1
--	At1g61110	tor 25-like	AC transc	cterized protein {ECO:0000313|EMBL:ESW31277.1}
--	At4g28500	ing protein	|PubMed:1	ain-containing protein 8 {ECO:0000313|EMBL:KHN1
--	At1g56010	COTYLED	n-containi	ED COTYLEDON 3-like {ECO:0000313|RefSeq:XP_
--	At1g69490	factor 29 [	AC029; Alt	in-containing protein 29 {ECO:0000313|EMBL:KYP4
--	At2g18060	g protein	CO:0000	eristem (NAM) protein {ECO:0000313|EMBL:OMO84
--	At4g29230	ein 73 iso	:0000303|	ain-containing protein 8 {ECO:0000313|EMBL:KYP61
--	At2g17040	factor 32 [	:0000303|	omain-containing protein 94 {ECO:0000313|EMBL:K
--	At4g29230	ein 73 iso	:0000303|	ain-containing protein 8 {ECO:0000313|EMBL:KYP61
--	--	protein 26-	--	--

--	At4g2850	g protein 7	|PubMed:	ain-containing protein 8 {ECO:0000313|EMBL:KHN15
--	At5g5395	TYLEDO	C domain-	PED COTYLEDON 2-like {ECO:0000313|RefSeq:XP_
--	--	g protein 7	--	--
--	At1g1226	in 7-like is	########	ain-containing protein 7 {ECO:0000313|EMBL:KYP55
lear transcr	--	unit A-10-l	--	iption factor Y subunit A-10 {ECO:0000313|EMBL:KY
lear transcr	At5g0651	unit A-10	anscription	iption factor Y subunit A-10 {ECO:0000313|EMBL:KY
lear transcr	At5g1284	bunit A-1	i1; AltNam	ription factor Y subunit A-9 {ECO:0000313|EMBL:KF
lear transcr	At1g7283	ubunit A-7	unit A-3; S	iption factor Y subunit A-3 {ECO:0000313|EMBL:KY
lear transcr	At4g1454	or Y subun	unit B-3; S	n factor Y subunit B-3-like {ECO:0000313|RefSeq:XP
lear transcr	At5g4764	r Y subuni	unit B-2; S	n factor Y subunit B-3-like {ECO:0000313|RefSeq:XP
--	At1g0798	otein 8 iso	bMed:265	aracterized protein {ECO:0000313|EMBL:BAT90596.
--	At1g5617	Y subunit	unit C-2; S	HAP5B {ECO:0000313|EMBL:ACB78194.1};
ear transcri	At1g0897	Y subunit	unit C-9; S	HAP5B {ECO:0000313|EMBL:ACB78194.1};
--	At5g4325	on subunit	--	sociated corepressor {ECO:0000313|EMBL:KYP7568
ethylene r	At3g0458	N4 [Arachi	4; EC=2.7.	ne receptor {ECO:0000256|PIRNR:PIRNR026389};
d amphipat	At1g2419	Sin3-like	Histone dea	0313|EMBL:KRH45772.1, ECO:0000313|EnsemblPla
--	At2g3335	protein 1-li	--	aracterized protein {ECO:0000313|EMBL:OIV89998.1
onent resp	At3g5704	egulator O	response re	cterized protein {ECO:0000313|EMBL:ESW09431.1}
--	At1g1927	like isofor	name: Full=	0313|EMBL:KRH15044.1, ECO:0000313|EnsemblPla
--	At1g1927	lated 1 [A	ame: Full=	XP_004509228.1, ECO:0000313|RefSeq:XP_0045092
--	At1g2419	otein Sin3	Histone dea	0313|EMBL:KRH45772.1, ECO:0000313|EnsemblPla
--	At5g5999	STANS-li	--	0313|EMBL:KRH22973.1, ECO:0000313|EnsemblPla
ethylene r	At3g0458	4 [Arachis	4; EC=2.7.	ne receptor {ECO:0000256|PIRNR:PIRNR026389};
onent resp	At3g5704	tor ORR1	response re	cterized protein {ECO:0000313|EMBL:ESW20933.1};
--	--	5A [Arac	AltName:	nscription factor {ECO:0000313|EMBL:ALA09269.1};
--	At5g5342	TANS-LIK	e: Full=Pr	motif protein {ECO:0000313|EMBL:AES58778.1};
ethylene r	At3g0458	4 [Arachis	4; EC=2.7.	ne receptor {ECO:0000256|PIRNR:PIRNR026389};
--	At1g7554	protein 20	Protein LO	0313|EMBL:KRH05902.1, ECO:0000313|EnsemblPla
--	At5g1072	e isoform	):0000305}	0313|EMBL:KRH61622.1, ECO:0000313|EnsemblPla
ine kinase	At5g3575	ase 4 [Arac	########	inase 1 {ECO:0000313|EMBL:ANQ89644.1}; Flags: F
--	At4g2731	TANS-LIK	tBBX31 {	0313|EMBL:KRH10724.1, ECO:0000313|EnsemblPla
--	At3g4810	regulator	response re	0313|EMBL:KRH03376.1, ECO:0000313|EnsemblPla
onent resp	At2g2518	tor ARR1	R22 {ECO:	t response regulator {ECO:0000256|PIRNR:PIRNR03
--	At1g1927	lated 1 [A	ame: Full=	protein 1 {ECO:0000313|EMBL:AKI32341.1}; Flags:
--	At2g3138	rotein 25 [	9}; AltNar	0313|EMBL:KRH23214.1, ECO:0000313|EnsemblPla
--	At1g1927	isoform X	ame: Full=	protein 1 {ECO:0000313|EMBL:AKI32341.1}; Flags:
--	--	rotein 32 [	--	0313|EMBL:KRH47291.1, ECO:0000313|EnsemblPla
--	At5g5718	PPARATU	OROPLAS	0313|EMBL:KRH19102.1, ECO:0000313|EnsemblPla
--	At1g1927	ated 1 [Ara	ame: Full=	XP_004509228.1, ECO:0000313|RefSeq:XP_0045092
--	At2g3335	in 1-like is	--	0313|EMBL:KRH33593.1, ECO:0000313|EnsemblPla
--	At5g2226	STERILI	PHD finge	protein At1g33420 family {ECO:0000313|EMBL:KYP
HAT com	At3g1474	o1 isoform	########	de-1 isoform X1 {ECO:0000313|RefSeq:XP_0045137
--	--	--	--	--
--	t5g58610_	thylation 1	########	in 4 {ECO:0000313|EMBL:KHN45144.1}; EC=3.6.4.
--	At5g3674	nding prot	########	aracterized protein {ECO:0000313|EMBL:OAY49724.

--	t5g58610_	7470574 is	########	aracterized protein {ECO:0000313|EMBL:OIW09535.1
somerase (I	--	At4g0211(	--	on regulator dpb11, putative {ECO:0000313|EMBL:KE
--	At5g5343(	ansferase A	l=Protein S	O:0000256|SAAS:SAAS00591749}; EC=2.1.1.43 {EC
--	At5g5343(	ansferase A	l=Protein S	O:0000256|SAAS:SAAS00591749}; EC=2.1.1.43 {EC
--	t5g58610_	LOC10764	########	aracterized protein {ECO:0000313|EMBL:OIW09535.1
somerase (I	--	n At4g021	--	on regulator dpb11, putative {ECO:0000313|EMBL:KE
tin-protein	At5g5343(	rase ATX3	l=Protein S	O:0000256|SAAS:SAAS00591749}; EC=2.1.1.43 {EC
--	At3g6067(	7483742 is	--	00313|EMBL:KRH37216.1, ECO:0000313|EnsemblPla
--	At3g6067(	LOC1076(	--	cterized protein {ECO:0000313|EMBL:ESW13927.1};
--	At3g6067(	At3g5080	--	protein LOC107953547 {ECO:0000313|RefSeq:XP_01
--	At3g6067(	OC107457	--	cterized protein {ECO:0000313|EMBL:ESW13927.1};
--	--	-like APRF	--	esponse regulator-like APRR5 {ECO:0000313|EMBL:
pseudo-res	At5g2447(	r-like APR	e regulato	esponse regulator-like APRR5 {ECO:0000313|EMBL:
--	At2g4350(	P2 [Arachi	e: Full=NI	aracterized protein {ECO:0000313|EMBL:KYP58419.1
--	At1g2064(	orm X1 [A	e: Full=NI	00313|EMBL:KRH60929.1, ECO:0000313|EnsemblPla
--	At2g4350(	2 [Arachis	e: Full=NI	aracterized protein {ECO:0000313|EMBL:KYP58419.1
--	At2g4350(	protein NI	e: Full=NI	aracterized protein {ECO:0000313|EMBL:KYP65998.1
--	At1g2064(	P4 [Arachi	e: Full=NI	00313|EMBL:KRH60929.1, ECO:0000313|EnsemblPla
--	At2g3381(	ing-like pr	Squamosa	moter-binding-like protein {ECO:0000256|PIRNR:PIR
--	At2g3381(	e protein 3	Squamosa	moter-binding-like protein {ECO:0000256|PIRNR:PIR
--	At5g4327(	e protein 1	Squamosa	promoter-binding-like protein {ECO:0000313|EMBL:AI
--	At5g4327(	ing-like pr	Squamosa	omoter-binding-like protein {ECO:0000313|EMBL:AI
--	At1g5316(	g protein 1	Squamosa	er-binding protein 1-like {ECO:0000313|RefSeq:XP_01
--	At5g5067(	g-like prot	Squamosa	promoter-binding-like protein {ECO:0000313|EMBL:AI
--	At2g3381(	g-like prot	Squamosa	binding-like protein {ECO:0000313|EMBL:AID59213.
--	At5g4327(	e protein 1	Squamosa	promoter-binding-like protein {ECO:0000313|EMBL:AI
--	At5g4327(	e protein 1	Squamosa	promoter-binding-like protein {ECO:0000313|EMBL:AI
--	At1g5316(	ing protein	Squamosa	er-binding-like protein {ECO:0000256|PIRNR:PIRNR(
--	At5g5067(	-like prote	Squamosa	promoter-binding-like protein {ECO:0000313|EMBL:AI
--	At5g5067(	g-like prot	Squamosa	promoter-binding-like protein {ECO:0000313|EMBL:AI
tin-protein	At5g5343(	se ATX5	l=Protein S	d protein {ECO:0000313|EMBL:KRG94189.1}; Flags
sine N-metl	At4g3086(	ansferase	Full=ASH1	O:0000256|SAAS:SAAS00591749}; EC=2.1.1.43 {EC
--	At3g5508(	unit N-met	########	aracterized protein {ECO:0000313|EMBL:KRH36236.
-bisphosph	At1g1403(	ge subunit	########	subunit N-methyltransferase {ECO:0000313|EMBL:KI
--	At4g2012(	ALLY AC	########	0313|EMBL:KRH37182.1, ECO:0000313|EnsemblPlan
N-methylt	At5g1724(	P 40-like	rotein SET	00313|EMBL:KRH17268.1, ECO:0000313|EnsemblPla
-bisphosph	At1g1403(	subunit N	########	0313|EMBL:KRH50559.1, ECO:0000313|EnsemblPla
N-methylt	At3g0767(	ge subunit	--	0000313|EMBL:KRH17729.1, ECO:0000313|Ensembl
nbination p	At3g2434(	CLASSY 4	ame: Full=	aracterized protein {ECO:0000313|EMBL:OIW07626.
11; ATP-d	At5g6675(	helicase I	########	0313|EMBL:KRH76800.1, ECO:0000313|EnsemblPlan
nbination p	At3g2434(	ein CLASS	ame: Full=	aracterized protein {ECO:0000313|EMBL:OIW07626.
repair prot	At5g4353(	regulator c	########	0313|EMBL:KRG90383.1, ECO:0000313|EnsemblPla
11; ATP-d	At5g6675(	licase DD	########	0313|EMBL:KRH76800.1, ECO:0000313|EnsemblPlan
repair prot	At5g4353(	egulator of	########	0313|EMBL:KRG90383.1, ECO:0000313|EnsemblPla
--	At3g5428(	Rab7-like	ne: Full=R	cterized protein {ECO:0000313|EMBL:ESW31760.1};

on repair pı	At1g03750	[Arachis i	Name: Full	l recombination protein RAD26-like {ECO:0000313|E
--	At3g54430	IORDIUM	Protein LA	0313|EMBL:KRH62844.1, ECO:0000313|EnsemblPla
--	At3g54430	ORDIUM 1	Protein LA	0313|EMBL:KRH62844.1, ECO:0000313|EnsemblPla
--	At4g22360	ng protein	--	racterized protein {ECO:0000313|EMBL:OMO64703.1
--	At5g63700_	08430 isof	ull=Uncha	0313|EMBL:KRH30822.1, ECO:0000313|EnsemblPla
--	At2g14880	it UAF30	); Short=At	aracterized protein {ECO:0000313|EMBL:OIW11184.1
--	At5g08430_	08430 isof	ull=Uncha	0313|EMBL:KRH09302.1, ECO:0000313|EnsemblPla
--	At2g14880	it UAF30	); Short=At	aracterized protein {ECO:0000313|EMBL:OIW11184.1
--	At5g63700_	At5g0843(	--	main-containing protein 44-like {ECO:0000313|RefSeq:
--	At4g22360	ng protein	--	racterized protein {ECO:0000313|EMBL:OMO64703.1
--	At3g48360	ontaining	-containing	main-containing protein 2-like {ECO:0000313|RefSeq:
--	At1g79000	se HAC1-	ferase HAC	aracterized protein {ECO:0000313|EMBL:OWM69462
--	At1g58100	or TCP8 [	ne: Full=Tr	0313|EMBL:KRH06094.1, ECO:0000313|EnsemblPla
--	At4g20270	threonine-p	kinase BA	protein {ECO:0000313|EnsemblPlants:GLYMA02G45(
--	--	r TCP14 [A	--	aracterized protein {ECO:0000313|EMBL:BAT76589.
--	--	or TCP3 [	e: Full=Tra	aracterized protein {ECO:0000313|EMBL:OIV99127.1
--	At1g58100	r TCP8 [A	ne: Full=Tr	0313|EMBL:KRH26342.1, ECO:0000313|EnsemblPla
--	--	or TCP14 [	--	aracterized protein {ECO:0000313|EMBL:BAT76589.
--	--	or TCP3 [	e: Full=Tra	aracterized protein {ECO:0000313|EMBL:OIV99127.1
te ZIM dor	At5g20900	3B [Arach	AltName:	CCT motif protein {ECO:0000313|EMBL:AES92112.1
--	At4g32570	form X2 [A	in TIFY 8	Protein TIFY 8 {ECO:0000313|EMBL:KYP39560.1};
te ZIM dor	--	Y 5A [Arac	AltName:	0313|EMBL:KRH11175.1, ECO:0000313|EnsemblPla
--	At2g34600	Y 5A [Arac	AltName:	0313|EMBL:KRH11175.1, ECO:0000313|EnsemblPla
te ZIM dor	At2g34600	5A [Arach	AltName:	0313|EMBL:KRH11175.1, ECO:0000313|EnsemblPla
te ZIM dor	At5g20900	3B [Arach	AltName:	CCT motif protein {ECO:0000313|EMBL:AES92112.1
--	At4g32570	oform X1 [	in TIFY 8	Protein TIFY 8 {ECO:0000313|EMBL:KYP39560.1};
; speckle-ty	At1g55760	n-containir	TB/POZ do	domain-containing protein 4 {ECO:0000313|EMBL:KY
--	At5g19330	RACTINC	T PROTEI	INTERACTING WITH ABF2-like {ECO:0000313|Re
--	At5g13060	RACTINC	T PROTEI	INTERACTING WITH ABF2-like {ECO:0000313|Re
--	At5g60050	protein At	TB/POZ do	ntaining protein At5g60050 {ECO:0000313|RefSeq:XI
--	At2g41370	in NPR5 [A	B/POZ dom	terized protein {ECO:0000313|EMBL:CCB55300.1};
--	At3g48360	-containing	-containing	l protein {ECO:0000313|EnsemblPlants:GLYMA05G0
; speckle-ty	At2g39760	ining prote	rotein 3; Al	domain-containing protein 3 {ECO:0000313|RefSeq:X
.; regulator	At2g41370	NPR5 [Ar	B/POZ dom	0313|EMBL:KRH72058.1, ECO:0000313|EnsemblPla
.; regulator	At2g41370	NPR5-like	B/POZ dom	rotein NPR5-like {ECO:0000313|RefSeq:XP_018844(
S-phase ki	CE10580	soform X1	ull=SKP1-l	P1 component {ECO:0000313|EMBL:OMO85395.1};
.; regulator	At5g45110	NPR3 [Ar	PR4; AltNa	atory protein NPR3 {ECO:0000313|EMBL:KHN22972
; speckle-ty	At2g39760	ining prote	rotein 3; Al	domain-containing protein 3 {ECO:0000313|RefSeq:X
; speckle-ty	At1g55760	main-conta	TB/POZ do	terized protein {ECO:0000313|EMBL:AFK49561.1};
.; regulator	At5g45110	in NPR3 [A	PR3; AltNa	atory protein NPR3 {ECO:0000313|EMBL:KHN22972
--	At5g28300	factor GTI	l=GT2-LIK	0313|EMBL:KRG96954.1, ECO:0000313|EnsemblPla
--	At1g76890	factor GT-	factor GT-	aracterized protein {ECO:0000313|EMBL:KYP50558.1
--	At3g58630	factor ASII	g protein 1	actor ASIL1-like isoform X2 {ECO:0000313|RefSeq:X
--	--	factor GT-	--	ption factor GT-2-like {ECO:0000313|RefSeq:XP_004
--	At1g76890	actor GTL2	factor GT-	0313|EMBL:KRG96954.1, ECO:0000313|EnsemblPla

--	--	r GTL2, pa	--	cterized protein {ECO:0000313|EMBL:EMJ09797.1};
--	--	factor GT-	--	ption factor GT-2-like {ECO:0000313|RefSeq:XP_004
--	--	actor GT-2	--	racterized protein {ECO:0000313|EMBL:KYP50557.1
--	At3g1004C	in DDB_G	--	0000313|RefSeq:XP_016691470.1, ECO:0000313|Ref
--	At5g0138C	factor GT-	factor GT-3	nscription factor GT-3b {ECO:0000313|EMBL:KHN4
--	--	factor GT-	--	--
--	At3g5863C	factor ASIL	g protein 1	actor ASIL1-like isoform X1 {ECO:0000313|RefSeq:X
--	At5g0138C	actor GT-3	factor GT-3	nscription factor GT-3b {ECO:0000313|EMBL:KHN4
--	At1g1607C	tein 8 [Ara	ll=Tubby-l	like protein 8 {ECO:0000313|RefSeq:XP_014500440.1
--	At1g1607C	tein 8 [Ara	ll=Tubby-l	like protein 8 {ECO:0000313|RefSeq:XP_014500440.1
--	--	e F-box pro	--	-like F-box protein {ECO:0000256|RuleBase:RU36112
--	--	e F-box pro	--	-like F-box protein {ECO:0000256|RuleBase:RU36112
--	At4g2819C	TALA 1-lie	: Full=Pro	ULTRAPETALA 1 {ECO:0000313|EMBL:KYP68829
--	At4g3155C	ription fact	cription fa	0313|EMBL:KRH19120.1, ECO:0000313|EnsemblPlan
WRKY tra	At5g0710C	ription fact	):0000303|	0313|EMBL:KRH76058.1, ECO:0000313|EnsemblPlan
--	At4g0445C	factor 6-li	l=WRKY	00313|EMBL:KRH56160.1, ECO:0000313|EnsemblPla
--	--	ption facto	--	transcription factor 23 {ECO:0000313|RefSeq:XP_00
--	At2g3059C	ription fact	cription fa	KY transcription factor 21 {ECO:0000313|EMBL:KHN
WRKY tra	At2g3726C	factor 44	otein TRA	ranscription factor 44 {ECO:0000313|EMBL:KYP708
--	At4g0445C	r 6 isoforn	l=WRKY	00313|EMBL:KRH36485.1, ECO:0000313|EnsemblPlan
WRKY tra	--	ription fact	--	00313|EMBL:KRH59972.1, ECO:0000313|EnsemblPla
--	At3g5640C	ription fact	cription fa	ctor WRKY57 {ECO:0000313|EMBL:DAA80460.1};
--	At1g8084C	factor 40 is	cription fa	transcription factor 40 {ECO:0000313|RefSeq:XP_00
WRKY tra	At5g5627C	SIBA2-like	O:0000303	00313|EMBL:KRH00919.1, ECO:0000313|EnsemblPla
--	At1g8084C	factor 40	cription fa	cterized protein {ECO:0000313|EMBL:ESW33089.1};
--	At2g3726C	n factor 44	otein TRA	nscription factor {ECO:0000313|EMBL:ALA09266.1}
--	At4g2381C	ription fact	cription fa	KY transcription factor 53 {ECO:0000313|EMBL:KYP
--	At5g2617C	factor 50 is	cription fa	racterized protein {ECO:0000313|EMBL:OIW09221.1
--	At2g3059C	ption facto	cription fa	KY transcription factor 21 {ECO:0000313|EMBL:KHN
WRKY tra	--	ription fact	--	00313|EMBL:KRH59972.1, ECO:0000313|EnsemblPla
--	At3g5871C	factor 69 is	cription fa	0313|EMBL:KRH27073.1, ECO:0000313|EnsemblPlan
--	At4g2664C	factor 20	cription fa	0313|EMBL:KRH73864.1, ECO:0000313|EnsemblPlan
--	At4g1107C	ption facto	cription fa	anscription factor {ECO:0000313|EMBL:ALA09256.1
--	At4g0445C	r 6 isoforn	l=WRKY	00313|EMBL:KRH36485.1, ECO:0000313|EnsemblPlan
--	--	ription fact	--	--
WRKY tra	At2g0334C	iption fact	nscription	aracterized protein {ECO:0000313|EMBL:BAT83325.
--	--	rotein RPS	--	00313|EMBL:KRH59108.1, ECO:0000313|EnsemblPla
--	At5g2411C	ription fact	cription fa	anscription factor {ECO:0000313|EMBL:ALA09263.1
--	At3g5640C	factor 70	cription fa	KY transcription factor 70 {ECO:0000313|EMBL:KYP
WRKY tra	At5g5627C	r SUSIBA2	O:0000303	cterized protein {ECO:0000313|EMBL:ESW11719.1};
--	At2g3059C	ription fact	cription fa	KY transcription factor 21 {ECO:0000313|EMBL:KHN
--	At4g2664C	factor 20 is	cription fa	0313|EMBL:KRH73864.1, ECO:0000313|EnsemblPlan
--	At4g3155C	ption facto	cription fa	RKY13 {ECO:0000313|EMBL:ABC26916.1};

XP_014516537.1};
nts:GLYMA12G07800.3};
nts:GLYMA12G07800.3};
1};
nts:GLYMA04G03070.2};
1};
J07644.1};
;
J07643.1};
J07643.1};
P_004496086.1};
};
;
1};
ling protein {ECO:0000313|EMBL:ABC69353.1};
)03.1};
P_004496086.1};
nts:GLYMA13G38030.1};
Seq:XP_004485859.1};
Seq:XP_004485859.1};

ES90761.2};
5.1};
3141.1};
;
J07644.1};

nts:GLYMA02G11060.1};
nts:GLYMA10G44041.2};
nts:GLYMA10G44041.2};
9};

)};

9};

)};
9};
9};

;
)};

nts:GLYMA14G08641.1};

[illegible]31454.1};
_004501577.1};
KHN24997.1};
nts:GLYMA04G36750.2};
ragment;
;

1};

nts:GLYMA20G24268.1};
ts:GLYMA11G13220.3};
1};
};

};

};
};
};

};

nts:GLYMA10G27910.3};
nts:GLYMA10G27910.3};
.1};
;
ts:GLYMA20G37550.1};

};
.1};
[illegible]91.1};
;
nts:GLYMA16G05390.2};
;
495284.1};
nts:GLYMA11G12450.3};
000313|EMBL:KHN17463.1};
;
[illegible]9886.1};
[illegible]9669.1};
25.1};
[illegible]05138.1};

36.1};
nts:GLYMA07G03081.1};
59.1};
0000313|EMBL:KHN04892.1};
.1};
495284.1};

68.1};
nts:GLYMA20G37550.1};
92.1};
nts:GLYMA16G02320.2};
7.1};
;

;
ion 1 {ECO:0000313|EMBL:ACJ39211.1};

N21635.1};
;
nts:GLYMA14G36370.1};
nts:GLYMA05G26490.1};
nts:GLYMA14G36370.1};

.};
};
45.1};
520018.1};
45.1};
nts:GLYMA19G42520.1};
05138.1};
;
3.1};
1};
;
nts:GLYMA13G44594.1};
52.1};
nts:GLYMA11G04690.1};
71.1};
25.1};
3.1};
YP40713.1};

1};
Fragment;
1};

ıts:GLYMA08G14840.4};
379.1};
285.2};
ИBL:KYP43908.1};
'23.1};

Fragment;
000313|RefSeq:XP_012571863.1};
ıts:GLYMA09G34170.1};

379.1};
l};

nts:GLYMA14G38460.1};
1};
ıts:GLYMA08G14840.4};
.};
3.1};
22.1};

908.1};
1};
04497095.1};
_014502470.1};
04497095.1};
_014502470.1};
014511026.1};
499844.1};
;
518806.1};
58360.1}; SubName: Full=Dof19 {ECO:0000313|EMBL:AJG01739.1};

'_016689061.1};
58360.1}; SubName: Full=Dof19 {ECO:0000313|EMBL:AJG01739.1};
l};
499844.1};
};
;
1};
499844.1};
518806.1};

ES72534.2};
1};

Flags: Fragment;
};
497141.1};
Fragment;
nts:GLYMA11G07350.2};
992};
L:AES82392.1};
;
;
992};
2};
1};
1};
.1};
1};
;
nts:GLYMA10G39710.5};
nts:GLYMA02G40960.3};
901.1};
nts:GLYMA01G06880.2};
1};
72.1};
1};
;
12.1};
;
1};
901.1};
14502723.1};
1};
};
;
.1};
.1};
588616.2};
1};
};
1};
nts:GLYMA18G07330.1};
ts:GLYMA03G33700.1};
nts:GLYMA02G40960.3};
};
nts:GLYMA10G29390.1};
nts:GLYMA04G06660.1};
1};

{ECO:0000313|EMBL:KHN03161.1};
)830.1};
;
588616.2};
)830.1};
KYP76693.1};
ıts:GLYMA14G37100.1};
ıts:GLYMA14G37100.1};
nts:GLYMA15G42820.1};
ıts:GLYMA01G44670.1};
1};

508403.1};
P_014507184.1};
.1};
.1};
1}; Flags: Fragment;
HN24104.1};
HN24104.1};
1};
04503105.1};
ts:GLYMA01G39040.1};
KYP53354.1};
EH30918.1};
1.1};
P53013.1};
nts:GLYMA14G39260.2};
3.1};
1};

5392};
04503105.1};
KYP53354.1};
nts:GLYMA19G32841.1};
KYP35731.1};
4979.1};
ts:GLYMA01G39040.1};
1.1};
1};
14520124.1};
ıts:GLYMA20G32770.1};
1};
1};
1};

).1};
).1};

):0000313|EMBL:KYP71767.1};

.};

1};
029.1};
38.1};
.};
1};
;
2574062.1};
37559.1};
nts:GLYMA13G16924.1};
1};
4508379.1};
nts:GLYMA01G35145.1};
;
nts:GLYMA14G10100.2};
;
1};
nts:GLYMA17G35090.2};
OX91227.1};
1};

HN06836.1};
014518680.1};
516480.1, ECO:0000313|RefSeq:XP_014516481.1};
nts:GLYMA06G20230.1};
P44343.1};
nts:GLYMA15G01960.1};
)4515372.1};
014518680.1};
KYP33458.1};
04509044.1};
P70362.1};

313|EMBL:GAV63258.1};
04509044.1};
ipper protein HAT5 isoform X2 {ECO:0000313|RefSeq:XP_004489298.1};
nts:GLYMA10G38280.2};
nts:GLYMA12G10710.1};
IN35655.1};

313|EMBL:GAV63258.1};
1};
HN06836.1};
ıts:GLYMA01G05230.1};
;
14502681.1};
1};
:ADC35600.1};

:ADC35600.1};
.1}; Flags: Fragment;

186.1}; Flags: Fragment;
neobox 13B2 {ECO:0000313|EMBL:AKN10293.1};
077.1};
)6266.1};
)6266.1};
.1};
1};
P_004497257.1};
Plants:GLYMA08G02550.1};
P_004497257.1};
ıts:GLYMA19G34210.1};
4499941.1};
ıts:GLYMA03G29190.1};
actor HSFB1 {ECO:0000313|EMBL:AFQ93674.1};
ıts:GLYMA19G34210.1};
)313|RefSeq:XP_014500016.1};
5626.1};
4499941.1};
actor HSFB1 {ECO:0000313|EMBL:AFQ93674.1};
1};
1};
31.1};
nts:GLYMA06G10660.2};
;
nts:GLYMA06G10660.2};
nts:GLYMA17G10030.2};
1};
ıts:GLYMA04G39830.1};
1};

ıts:GLYMA01G40010.1};
1};
1};

nts:GLYMA20G26910.1};
4488.1};
1};

nts:GLYMA04G39830.1};
3652.1};
nts:GLYMA01G40010.1};

nts:GLYMA14G36221.1};
03.1};
1};
nts:GLYMA06G02990.1};
03.1};
nts:GLYMA06G02990.1};
1};
1};
nts:GLYMA14G36221.1};

592.1};
592.1};

43.1};
43.1};

XP_014505348.1};

: Fragment;
: Fragment;
;
93.1};
;
nts:GLYMA06G16820.1};
75.1};
;
.1};
G23455.1};
5.1};
15544.1};
.1};
.1};
4.1};

nts:GLYMA15G04620.1};
6.1};

;
9242.1};
1};

ıts:GLYMA06G08660.1};

71.1};
1};
95885.1};
ıts:GLYMA03G38410.1};
900.1};
6.1};
08935.1};
XP_004512739.1};
1};
5266.1};
};
ıts:GLYMA03G38410.1};
1};

.1};
ıts:GLYMA06G08660.1};
1};
nts:GLYMA11G14760.7};
ıgs: Fragment;
1};
)4509512.1};

;
.4502167.1};
2.1};
1};
gs: Fragment;
.1};
)62.1};

.1};
12.1};

2.1};
1};
2.1};
};
57930.1};

.1};
[illegible]
04488477.1};
04509512.1};
1.1};
1.1};
.1};
;
3620.2};
14497474.1};
nts:GLYMA14G39080.1};
5267.1};
XP_012571948.1};
nts:GLYMA05G00930.2};
1};
14491001.1};
3893.1};
5267.1};
nts:GLYMA09G37050.1};
14497474.1};
3620.2};
04487194.1};
tor 29 {ECO:0000313|RefSeq:XP_004490093.1};
Y13097.1};
04487194.1};
1};
1};
1};
_014495173.1};
9961.1};
nts:GLYMA10G36050.1};
99395.1};
1};
nts:GLYMA05G00930.2};
1};
;
5497.1};
_014495173.1};
5399.1};
4594.1};
394.1};
YP68073.1};
394.1};

5497.1};
_014500628.1};

5393.1};
YP56960.1};
YP56960.1};
IN19456.1};
P62217.1};
_014523242.1};
_014523242.1};
1};

3.1};

nts:GLYMA08G40390.4};
.};
;
nts:GLYMA14G07060.2};
29.1, ECO:0000313|RefSeq:XP_012573712.1};
nts:GLYMA08G40390.4};
nts:GLYMA13G40610.2};

;
;

nts:GLYMA17G37430.2};
nts:GLYMA04G06190.2};
ragment;
nts:GLYMA15G07140.1};
nts:GLYMA17G10170.1};
5392};
Fragment;
nts:GLYMA13G41980.1};
Fragment;
nts:GLYMA07G02320.1};
nts:GLYMA13G00520.2};
29.1, ECO:0000313|RefSeq:XP_012573712.1};
nts:GLYMA10G26990.1};
33289.1};
38.1};

12 {ECO:0000313|EMBL:KHN45144.1};
1};

1};
H43208.1};
O:0000256|PROSITE-ProRule:PRU00899, ECO:0000256|SAAS:SAAS00591749};
O:0000256|PROSITE-ProRule:PRU00899, ECO:0000256|SAAS:SAAS00591749};
1};
H43208.1};
O:0000256|PROSITE-ProRule:PRU00899, ECO:0000256|SAAS:SAAS00591749};
nts:GLYMA09G05781.1};
;
6744379.1};
;
KYP38831.1};
KYP38831.1};
1};
nts:GLYMA04G01910.3};
1};
1};
nts:GLYMA04G01910.3};
NR037575};
NR037575};
D59210.1};
D59216.1};
8814874.1};
D59221.1};
1}; Flags: Fragment;
D59211.1};
D59210.1};
37575};
D59219.1};
D59219.1};
: Fragment;
O:0000256|SAAS:SAAS00591749};
1};
H30792.1};
ts:GLYMA09G05510.1};
nts:GLYMA14G39060.1};
nts:GLYMA07G35510.1};
Plants:GLYMA13G11740.1};
1};
ts:GLYMA01G38150.1};
1};
nts:GLYMA20G21940.1};
ts:GLYMA01G38150.1};
nts:GLYMA20G21940.1};
;

MBL:KHN28113.1};
nts:GLYMA04G17885.2};
nts:GLYMA04G17885.2};
1};
nts:GLYMA11G33015.6};
l};
nts:GLYMA16G33391.2};
l};
XP_004494744.1};
1};
XP_004515848.1};
2.1};
nts:GLYMA16G00580.1};
)10.1};
1};
l};
nts:GLYMA12G28970.2};
1};
l};
.};

nts:GLYMA15G09980.1};
nts:GLYMA15G09980.1};
nts:GLYMA15G09980.1};
.};

'P76257.1};
fSeq:XP_014524181.1};
fSeq:XP_004493669.1};
?_014493458.1};

2863.1};
XP_004503177.1};
nts:GLYMA02G34135.1};
)30.1};

2.1};
XP_004503177.1};

2.1};
nts:GLYMA19G43280.1};
.};
P_004505494.1};
496473.1};
nts:GLYMA19G43280.1};

496473.1};
};
Seq:XP_016691471.1};
2520.1};

P_004505493.1};
2520.1};
1};
1};
5};
5};
.1};
nts:GLYMA13G00380.1};
nts:GLYMA01G31921.1};
nts:GLYMA06G46420.2};
4495894.1};
32331.1};
31.1};
nts:GLYMA09G00820.1};
nts:GLYMA05G37390.2};
SubName: Full=WRKY transcription factor {ECO:0000313|EMBL:ALA09265.1};
4501381.1};
nts:GLYMA18G47741.1};
;
};
58634.1};
};
32331.1};
nts:GLYMA05G37390.2};
nts:GLYMA12G33990.1};
ts:GLYMA02G46690.1};
};
nts:GLYMA09G00820.1};

1};
nts:GLYMA05G29921.2};
};
76652.1};
;
32331.1};
ts:GLYMA02G46690.1};
