# Supplementary figures and images for: Transcriptome and Metabolome Analysis Unveil Anthocyanin Metabolism in Pink and Red Testa of Peanut (Arachis hypogaea L.)
Source: Int J Genomics. 2021 Aug 6;2021:5883901. doi: 10.1155/2021/5883901 (PMC8363441; doi:10.1155/2021/5883901)

GO Classification

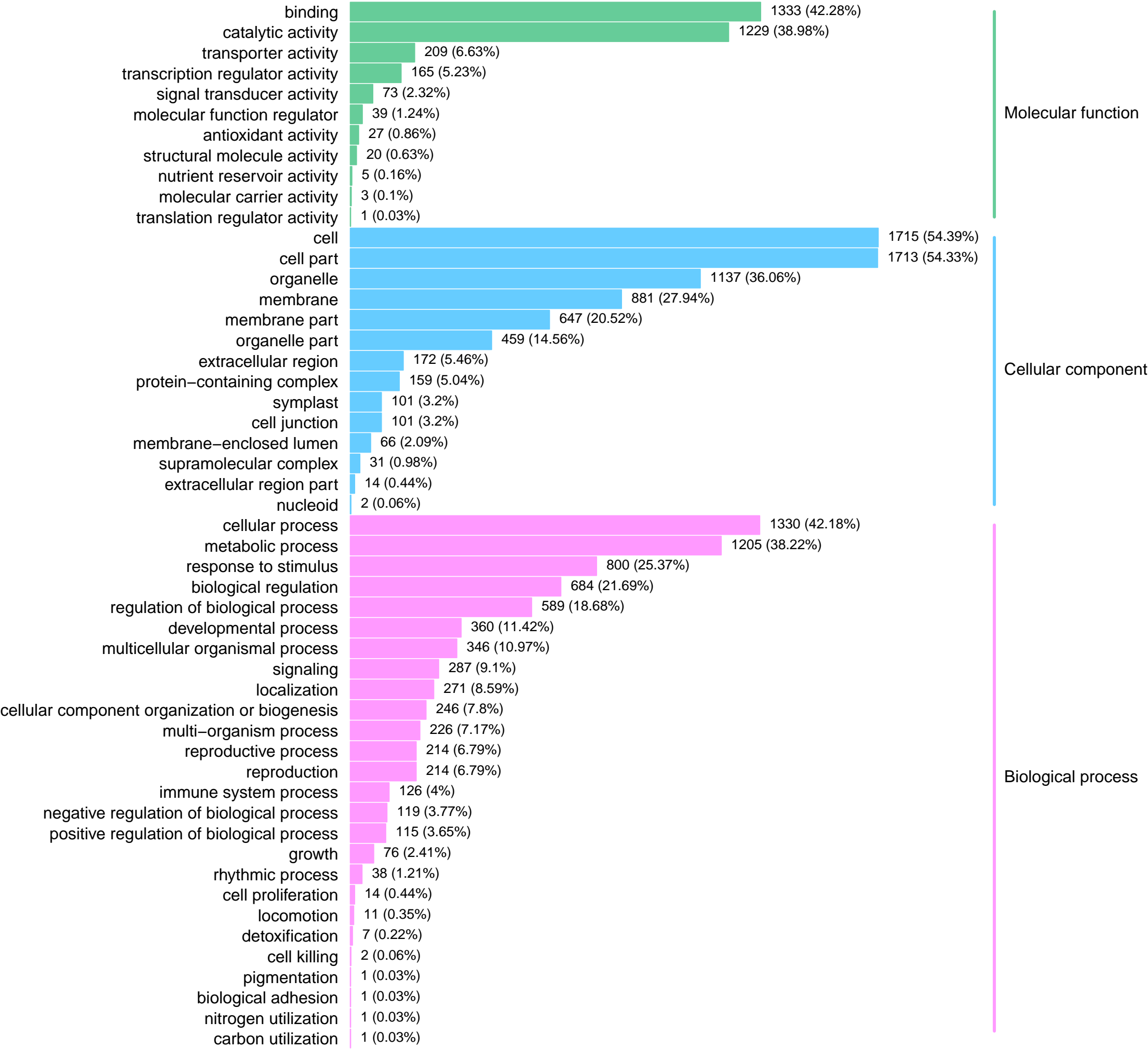

0 5 10 15 20 25 30 35 40 45 50 55  
Percent (%)

Supplement: Supplementary Materials — Supplementary Figure 1: GO classification of differentially expressed genes of R7 stage of Shanhua15 and Zhonghua12 testa. Supplementary Figure 2: COG functional classification of differentially expressed genes of R4 and R7 stages of Shanhua15 and Zhonghua12 testa. S4, Shanhua15 R4 pod period; Z4, Zhonghua12 R4 pod period; S7, Shanhua15 R7 pod period; Z7, Zhonghua12 R7 pod period. Supplementary Table 1: primers for qRT-PCR verification. Supplementary Table 2: significantly changed metabolites between Shanhua15 and Zhonghua12 testa. Supplementary Table 3: summary of the sequencing and de novo assembly. Supplementary Table 4: statistics of GO Enrichment in the testa of R4 and R7 stages of Shanhua15 and Zhonghua12 peanut. Supplementary Table 5: DEGs of flavonoid and anthocyanin biosynthesis in the testa of R4 and R7 stages of Shanhua15 and Zhonghua12 peanut. Supplementary Table 6: differentially expressed transcription factors in the testa of R4 and R7 stages of Shanhua15 and Zhonghua12 peanut. [file 5883901.f1.zip › 5883901.f1/Fig.S1.pdf]

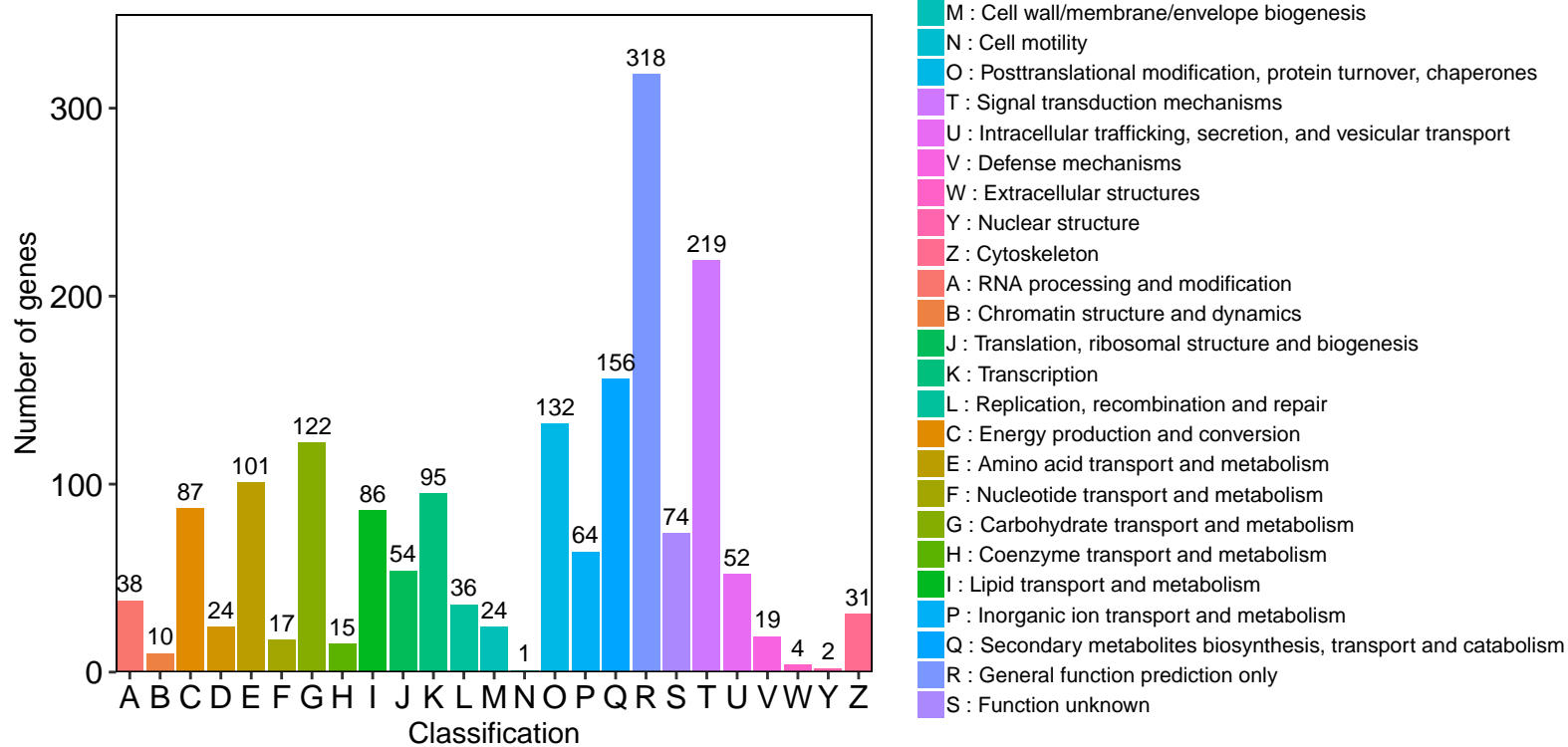

Supplement: Supplementary Materials — Supplementary Figure 1: GO classification of differentially expressed genes of R7 stage of Shanhua15 and Zhonghua12 testa. Supplementary Figure 2: COG functional classification of differentially expressed genes of R4 and R7 stages of Shanhua15 and Zhonghua12 testa. S4, Shanhua15 R4 pod period; Z4, Zhonghua12 R4 pod period; S7, Shanhua15 R7 pod period; Z7, Zhonghua12 R7 pod period. Supplementary Table 1: primers for qRT-PCR verification. Supplementary Table 2: significantly changed metabolites between Shanhua15 and Zhonghua12 testa. Supplementary Table 3: summary of the sequencing and de novo assembly. Supplementary Table 4: statistics of GO Enrichment in the testa of R4 and R7 stages of Shanhua15 and Zhonghua12 peanut. Supplementary Table 5: DEGs of flavonoid and anthocyanin biosynthesis in the testa of R4 and R7 stages of Shanhua15 and Zhonghua12 peanut. Supplementary Table 6: differentially expressed transcription factors in the testa of R4 and R7 stages of Shanhua15 and Zhonghua12 peanut. [file 5883901.f1.zip › 5883901.f1/Fig.S2.pdf]
