# Supplementary material for: Transcriptome and Metabolome Analysis Unveil Anthocyanin Metabolism in Pink and Red Testa of Peanut (Arachis hypogaea L.)
Source: Int J Genomics. 2021 Aug 6;2021:5883901. doi: 10.1155/2021/5883901 (PMC8363441; doi:10.1155/2021/5883901)
Supplement: Supplementary Materials — Supplementary Figure 1: GO classification of differentially expressed genes of R7 stage of Shanhua15 and Zhonghua12 testa. Supplementary Figure 2: COG functional classification of differentially expressed genes of R4 and R7 stages of Shanhua15 and Zhonghua12 testa. S4, Shanhua15 R4 pod period; Z4, Zhonghua12 R4 pod period; S7, Shanhua15 R7 pod period; Z7, Zhonghua12 R7 pod period. Supplementary Table 1: primers for qRT-PCR verification. Supplementary Table 2: significantly changed metabolites between Shanhua15 and Zhonghua12 testa. Supplementary Table 3: summary of the sequencing and de novo assembly. Supplementary Table 4: statistics of GO Enrichment in the testa of R4 and R7 stages of Shanhua15 and Zhonghua12 peanut. Supplementary Table 5: DEGs of flavonoid and anthocyanin biosynthesis in the testa of R4 and R7 stages of Shanhua15 and Zhonghua12 peanut. Supplementary Table 6: differentially expressed transcription factors in the testa of R4 and R7 stages of Shanhua15 and Zhonghua12 peanut. [file Table S1.pdf › 5883901.f1/Table S1.pdf]

Table S1 Primers for qRT-PCR verification		
Gene ID	Gene Name	Primer Sequenc
		Forward
	ACT11	TAAGAACAATGTTGCCATACAGA
arahy.AYA1A5	C4H	TTTGGTGTGGGGAGAAGGAG
arahy.6JHV2K	CHI	GGTTTCAGAAAATTGTGTGGCA
arahy.8F7PE4	F3H	TTTGGTGCTGGGAGAAGGAT
arahy.LDV9QN	DFR	AAGCCCACAACAAACGGATT
arahy.AQ6B1J	ANS	CCTATTTGTCCCCAGCCAGA
arahy.IK60LM	ANR	TGGCAGAGAAGGAATCAGCT
arahy.4WXU8P	FLS	CCATTCCAGCCATGTTCGTT
arahy.26781N	bHLH	CGTTCGAGGACACCATCAAC
arahy.SJGE27	MYB	GTCTCTGATAGCTGGGAGGC

ce（5'-3'）
Reverse
GTTGCCTTGGATTATGAGC
CCCTCCTTTCTCAGTGGTGT
CTGGTGGGAAGTTTTGATCCT
ACCCTCAAGCTCCCAATCAA
AACGTCACTCCACCATGTCT
ATGGAATCCGGGACACACTT
ACTTTGTTTTGGAGGGGCAA
GGCCTCATAAACCTCCCTGA
ACCTTCCTCTTCTCATGCCC
GAAGTTGATGGTTCCCCTGC
